# Population genomics demystifies the defoliation phenotype in the plant pathogen *Verticillium dahliae*

**DOI:** 10.1111/nph.15672

**Published:** 2019-02-25

**Authors:** Dan‐Dan Zhang, Jie Wang, Dan Wang, Zhi‐Qiang Kong, Lei Zhou, Geng‐Yun Zhang, Yue‐Jing Gui, Jun‐Jiao Li, Jin‐Qun Huang, Bao‐Li Wang, Chun Liu, Chun‐Mei Yin, Rui‐Xing Li, Ting‐Gang Li, Jin‐Long Wang, Dylan P. G. Short, Steven J. Klosterman, Richard M. Bostock, Krishna V. Subbarao, Jie‐Yin Chen, Xiao‐Feng Dai

**Affiliations:** ^1^ Laboratory of Crop Verticillium Wilt Institute of Food Science and Technology Chinese Academy of Agricultural Sciences Beijing 100193 China; ^2^ Genomics BGI‐Shenzhen Shenzhen 518083 China; ^3^ Department of Biology Duke University Durham NC 27708 USA; ^4^ Department of Plant Pathology University of California, Davis c/o US Agricultural Research Station Salinas CA 93905 USA; ^5^ United States Department of Agriculture Agricultural Research Service Crop Improvement and Protection Research Unit Salinas CA 93905 USA; ^6^ Department of Plant Pathology University of California Davis CA 93905 USA

**Keywords:** defoliating phenotype, lineage‐specific genes, *N*‐acylethanolamines (NAEs), secondary metabolites, *Verticillium dahliae*

## Abstract

- *Verticillium dahliae* is a broad host‐range pathogen that causes vascular wilts in plants. Interactions between three hosts and specific *V. dahliae* genotypes result in severe defoliation. The underlying mechanisms of defoliation are unresolved.
- Genome resequencing, gene deletion and complementation, gene expression analysis, sequence divergence, defoliating phenotype identification, virulence analysis, and quantification of *V. dahliae* secondary metabolites were performed.
- Population genomics previously revealed that G‐LSR2 was horizontally transferred from the fungus *Fusarium oxysporum* f. sp. *vasinfectum* to *V. dahliae* and is exclusively found in the genomes of defoliating (D) strains. Deletion of seven genes within G‐LSR2, designated as *VdDf* genes, produced the nondefoliation phenotype on cotton, olive, and okra but complementation of two genes restored the defoliation phenotype. Genes *VdDf5* and *VdDf6* associated with defoliation shared homology with polyketide synthases involved in secondary metabolism, whereas *VdDf7* shared homology with proteins involved in the biosynthesis of *N*‐lauroylethanolamine (*N*‐acylethanolamine (NAE) 12:0), a compound that induces defoliation. NAE overbiosynthesis by D strains also appears to disrupt NAE metabolism in cotton by inducing overexpression of fatty acid amide hydrolase.
- The *VdDf*s modulate the synthesis and overproduction of secondary metabolites, such as NAE 12:0, that cause defoliation either by altering abscisic acid sensitivity, hormone disruption, or sensitivity to the pathogen.

## Introduction

*Verticillium dahliae* is a widely distributed soilborne pathogenic fungus that invades xylem vessels of susceptible plants, causing an intractable vascular wilt disease (Fradin & Thomma, 2006; Klosterman *et al*., 2009). Over 200 plant species, including many economically important agricultural crops, are infected by *V. dahliae* (Fradin & Thomma, 2006; Klosterman *et al*., 2009; Inderbitzin & Subbarao, 2014). *V. dahliae* produces numerous microsclerotia in infected plant tissues, which are released into the soil with the decomposition of crop residue and can survive in the soil for at least 15 yr (Fradin & Thomma, 2006; Inderbitzin & Subbarao, 2014). The melanized resting structures produced by the fungus germinate in the presence of root exudates, and the emerging hyphae penetrate roots through the tip or through sites on lateral roots (Bishop & Cooper, 1983). After crossing the root cortex, hyphae grow into the xylem vessels. The hyphae remain exclusively in these vessels and produce conidia, which are transported acropetally within the xylem throughout the plant (Fradin & Thomma, 2006). This leads to characteristic symptoms that include wilting, stunting, leaf chlorosis and necrosis, vein clearing, and vascular discoloration (Schnathorst & Mathre, 1966; Fradin & Thomma, 2006).

Historically, pathotypes among *V. dahliae* strains have been classified based on the types of plant symptoms observed, and population genetic analyses typically support this classification. The *V. dahliae* strains that infect some plant species (cotton (*Gossypium hirsutum*), okra, and olive) are classified as defoliating (D) or nondefoliating (ND) based on their ability to completely defoliate or cause leaf wilt with partial or no defoliation, respectively (Jiménez‐Díaz *et al*., 2012). In cotton, the D pathotype was first described as a genetically distinct strain in the early 1960s (Schnathorst & Mathre, 1966). Compared with the ND pathotype, the D pathotype causes mortality more rapidly, and infections caused by the D pathotype result in greater yield losses (Bejarano‐Alcázar *et al*., 1995).

*Verticillium dahliae* has a highly clonal population structure with little or no evidence of recombination (de Jonge *et al*., 2013; Milgroom *et al*., 2014; Short *et al*., 2015), and investigations of the population structure by heterokaryon incompatibility (het) loci or microsatellite loci showed that isolates of *V. dahliae* can be divided into several clonal lineages (Joaquim & Rowe, 1991; Milgroom *et al*., 2014; Short *et al*., 2015). In addition, isolates of *V. dahliae* are classified into race 1 and race 2 based on the response of differential tomato (*Solanum lycopersicum*) and lettuce cultivars, and the compatible or incompatible reactions are conditioned by an assortment of resistance genes (*R*‐genes) in the host (Diwan *et al*., 1999). Population analyses further revealed that the D pathotype isolates belonged to the vegetative compatibility group 1A (VCG 1A), and defoliation in cotton, okra, and olive is limited to isolates within VCG 1A (Schnathorst & Mathre, 1966; Jiménez‐Díaz *et al*., 2006; Korolev *et al*., 2008). This is in contrast to other VCG groups, which generally cause wilting without defoliation (Schnathorst & Mathre, 1966; Korolev *et al*., 2008; Jiménez‐Díaz *et al*., 2011); ND and D strains of *V. dahliae* from cotton sort as races 1 and 2, respectively (Hu *et al*., 2015).

The physiological and biochemical processes of leaf senescence and defoliation are complex and are linked to crop yield (Lewis *et al*., 2006). During leaf abscission that occurs naturally as part of the plant's developmental sequence of maturation and senescence, nutrients are recycled from source tissues to reproductive organs (Munné‐Bosch & Alegre, 2004). Phytohormones (e.g. abscisic acid (ABA), ethylene) coordinate leaf senescence and defoliation, and external factors can induce these processes as well. Phytotoxic compounds (atrazine, thidiazuron, etc.), abiotic stresses (drought, osmotic stress, low light, salinity, etc.), and biotic stresses (e.g. pathogens and pests) can also promote leaf senescence and defoliation (Lim *et al*., 2007; Sade *et al*., 2017).

Among the biotic stresses, pathogen infection commonly results in early leaf chlorosis and defoliation (Bertamini *et al*., 2002; Bertaccini *et al*., 2014). These symptoms are in part related to water blockage caused by the colonization and proliferation of pathogenic microbes in the xylem (Fradin & Thomma, 2006; Klosterman *et al*., 2009), but they can also be caused by secreted toxins from *V. dahliae* (Zhang D. D. *et al*., 2016; Zhang W. Q. *et al*., 2017). The deleterious effects of some *V. dahliae* secondary metabolites have been reported (Bhatnagar *et al*., 2003; Zhang *et al*., 2012). In general, fungal secondary metabolite synthesis, transport, and secretion require the activity of various oxidoreductases and transporters (Yu *et al*., 1995; Desjardins *et al*., 1996; Proctor *et al*., 1999; Brown *et al*., 2004).

Several studies have investigated molecular mechanisms that underpin the pathogenicity and virulence of *V. dahliae*, including the involvement of secreted proteins, a battery of cell‐wall‐degrading enzymes, transcription factors, and membrane receptors (Klimes *et al*., 2015). In addition, mechanisms of host adaptation in *V. dahliae* reveal that this asexual pathogen may evolve through chromosomal rearrangement or horizontal gene transfer from other pathogens that enables rapid development of novel effector genes contributing to virulence (de Jonge *et al*., 2012, 2013). Comparative genomic analyses suggested that genes for signaling/transcriptional regulation and iron/lipid metabolism encoded by lineage‐specific (LS) regions play important roles in niche and host adaptation (Klosterman *et al*., 2011), and some LS genes are clearly upregulated *in planta* (de Jonge *et al*., 2012, 2013). Despite these advances, the mechanistic bases for the defoliation phenotype in *V. dahliae* are unresolved.

The D and ND pathotypes are associated with specific genomic DNA sequences (Pérez‐Artéz *et al*., 2000). Seven virulence factors are encoded in an LS region in the D *V. dahliae* strain Vd991 from cotton (*G. hirsutum*), known as G‐LSR2. Some of the gene products encoded within the G‐LSR2 region share homology with proteins involved in redox reactions that contribute to this isolate's virulence and adaptation to cotton (Chen *et al*., 2018).

The objectives of this study were to: (1) confirm the association of the LS region of G‐LSR2 exclusively to D pathotypes by whole‐genome resequencing; (2) investigate the function of seven cotton‐specific virulence factors in G‐LSR2 in defoliation; (3) investigate the evolution of defoliation‐associated genes between *V. dahliae* and the putative origin of G‐LSR2; and (4) examine the role of secondary metabolites encoded by G‐LSR2 genes in defoliation.

## Materials and Methods

### Fungal strains and culture conditions

*V. dahliae* Vd991 and an additional 75 isolates were collected from infected cotton plants in nine provinces in China (Supporting Information Table S1). The *V. dahliae* reference strains JR2 and VdLs.17 are from tomato and lettuce (*Lactuca sativa*) (Bhat & Subbarao, 1999; de Jonge *et al*., 2013), respectively. Five strains of *Fusarium oxysporum* f. sp. *vasinfectum* (FOV05, FOV07, FOV16, FOV17, and FOV18) were isolated from cotton plants. Unless specified otherwise, cultures of these fungi were maintained on potato dextrose agar (PDA) or potato dextrose broth (PDB) at 25°C before use.

### Molecular validation of D and ND phenotypes

Genomic DNA was extracted from fresh mycelia using a cetyl trimethylammonium bromide method (Springer, 2010). The primers described by Pérez‐Artéz *et al*. (2000) were employed to identify D and ND strains of *V. dahliae*. All PCRs were performed separately in 25 μl reaction volumes containing 12.5 μl of 2 × FastPfu Fly PCR SuperMix (TransStart, Beijing, China), 1 μl of genomic DNA (*c*. 30 ng μl^−1^), and 1 μl of each primer at a concentration of 10 μM. The annealing temperature was 58°C for both D and ND primers. The D and ND primers and other primers used in this study are listed in Table S1.

The D/ND phenotypes were validated on cotton seedlings using root‐dip inoculation (Hu *et al.,* 2015). Briefly, susceptible cotton plants (*G. hirsutum* cv Junmian No. 1) were maintained in a glasshouse at 28°C under a 14 h : 10 h light : dark photoperiod for 4 wk. Seedlings were gently uprooted, washed, and dipped into 1 × 10^7^ conidia ml^−1^ suspension (5 ml per seedling) for 5 min. Three independent replicates consisting of 12 plants each were inoculated for each *V. dahliae* isolate. Seedlings treated with sterile distilled water were used as controls. Seedlings were maintained at 25°C under a 14 h : 10 h light : dark photoperiod. Defoliation was assessed 4 wk after inoculation. Fungal biomass in cotton roots was determined as described by Santhanam *et al*. (2013). Quantitative PCR (qPCR) was performed using the qPCR SYBR premix Ex Taq II kit (TaKaRa, Tokyo, Japan) with primers from the cotton, okra and olive*18S* gene (Table S1). Differences between inoculated and noninoculated treatment groups were considered significant if paired Student's *t*‐test probability was ≤0.05. Presence or absence of vascular discoloration in shoots was assessed visually at 4 wk after inoculation.

To identify the D/ND phenotypes on olive and okra, 3‐wk‐old susceptible okra plants (Qiukui 101) and 1‐yr old olive seedlings (*Olea europaea* ‘Leccino’) were also inoculated with 1 × 10^7^ conidia ml^−1^. Roots were dipped in *V. dahliae* isolate suspensions (5 ml per seedling) for 5 min and then maintained at 25°C under a 14 h : 10 h light : dark photoperiod for 3 wk and defoliation was assessed.

### Genome resequencing and mapping

For *V. dahliae* genome resequencing, *c*. 5 μg of genomic DNA was used for each isolate to construct paired‐end sequencing libraries with insert sizes of 500 bp according to the manufacturer's instructions (Illumina, San Diego, CA, USA). Cluster generation, template hybridization, isothermal amplification, linearization, blocking, denaturation, and hybridization of the sequencing primers were performed according to the manufacturer's instructions (Illumina). The solexapipeline‐0.3 was used to call bases for 90 bp reads from raw fluorescent images. The insert size distribution of each library was determined by eland in solexapipeline. Reads were aligned to the Vd991 reference genome (Chen *et al*., 2018) using the SOApaligner (Li *et al*., 2009) with the following parameters: soap2.20 –p 4 –a 1.fq –b 2.fq –D IRGSP_chromosomes_build04.fa.index –o sample.soap ‐2 sample.single –u unmapped.fa –m 435*^1^ –x 501*^1^ –s 35 –l 24 –v 7 (*^1^: the insert size was estimated by eland and served as input for each library). To identify the gene absences in the G‐LSR2 region within the genomes of *V. dahliae* isolates, the coverage depth and breadth (mapping length/gene length) of each gene (including 20 flanking genes) were calculated. Reference genome G‐LSR2 regions with corresponding coverage depth <2× and breadth <25% in resequenced genomes were considered absent.

### Sequence divergence of *VdDf* homologues from *F. oxysporum* f. sp. *vasinfectum*

Putative homologs of the seven genes within G‐LSR2 (*VEDA_05193*–*VEDA_05199*; Chen *et al*. (2018)) hereinafter referred to as *Verticillium dahliae Defoliation‐associated* genes 1–7 (*VdDf1*–*VdDf7*) were amplified from the genomic DNA of five isolates of *F. oxysporum* f. sp. *vasinfectum* (FOVA_5, FOVA_7, FOVA_16, FOVA_17, and FOVA_18). The PCR was conducted by using the primers for cloning *VdDf1*–*VdDf7*, with an initial 94°C denaturation step for 10 min, followed by 36 cycles of 94°C for 30 s, 55°C for 1–2 min, and 72°C for 3 min. The protein sequences were deduced on the basis of *VdDf1*–*VdDf7* sequences. The sequence polymorphisms were identified by comparison with reference sequences.

### Fungal transformation

Gene deletion strains were produced using a homologous recombination following Mullins & Kang (2001). *VdDf1*–*VdDf7* were independently deleted, and *VdDf5* and *VdDf6* were deleted together (*VdDf5_6*). To generate the deletion constructs, the flanking sequences of corresponding genes/sequences were amplified from the Vd991 genomic DNA and integrated with the hygromycin cassette using fusion PCR (Liu *et al*., 2013). The amplified products were then cloned into the pGKO2‐gateway vector (Khang *et al*., 2005). For the complementation transformants, the sequence of *VdDf5* and *VdDf6* together accompanied by two flanking sequences (1765 bp upstream and 1186 bp downstream) was amplified from *V. dahliae* strain Vd991 genomic DNA and cloned into the binary vector pCOM that carries geneticin resistance (Zhou *et al*., 2013) and reintroduced to the *∆Dfs‐1* (constructed by the homologous recombination following Chen *et al*., 2018), VdLs.17 and VDG78 strains. For ectopic expression of *VdDH1*, the full‐length sequence was amplified from the Vd991 genomic DNA, cloned into the binary vector pCT‐HN with the *TrpC*, constitutive promoter, and integrated into the *∆Dfs‐1*,* ∆Df5_6‐1*, VdLs.17 and VDG78 strains. For ectopic expression of *F. oxysporum* f. sp*. vasinfectum* genes in *VdDf5* and *VdDf6* (*Df5_6* ^*FO*^), the homologous genes identified in *F. oxysporum f. sp. vasinfectum* FOV05, and two flanking sequences (1773 bp upstream and 1186 bp downstream) were amplified from genomic DNA and cloned into the binary vector pCOM that carries geneticin resistance (Zhou *et al*., 2013). These were reintroduced into the VDG78 and VdLs.17 strains. *Agrobacterium tumefaciens*‐mediated transformation of *V. dahliae* was conducted as described previously (Liu *et al*., 2013), and the transformants were selected on PDA (potato, 200 g l^−1^, glucose, 20 g l^−1^, agar, 15 g l^−1^) supplemented with antibiotics (60 μg ml^−1^ hygromycin or 50 μg ml^−1^ geneticin). Homologous recombination of the deletion mutants and ectopic transformants were verified by PCR with the corresponding primers (Table S1).

### Gene expression analysis

To analyze gene expression in *V. dahliae* in response to cotton, 4‐wk‐old cotton seedlings were root dip‐inoculated with 5 ml of 1 × 10^7^ conidia ml^−1^ from the wild‐type strains Vd991 and VDG78, and the gene deletion mutants *∆Df*s, *∆Df5‐1*,* ∆Df6‐1* and *∆Df5_6*. The roots were harvested at 12, 24, 48, 120, and 144 h post‐inoculation and flash frozen in liquid nitrogen (N_2_) for RNA extraction. *V. dahliae* strains cultured on PDA were used as controls. After grinding, 100 mg of ground material was used for total RNA extraction using the AxyPreP Multisource Total RNA Miniprep Kit (Axygen, New York, NY, USA) and first‐strand complementary DNA was synthesized using a RevertAid First cDNA Synthesis Kit (Thermo, Vilnius, (EU) Lithuania). The transcript levels of the different genes examined during different infection stages were determined using the qPCR SYBR premix Ex Taq II kit (TaKaRa) by reverse transcription (RT)‐ qPCR with the corresponding primers (Table S1), using the $2^{-\Delta \Delta C_{T}}$ method (Livak & Schmittgen, 2001). *V. dahliae* elongation factor 1‐α (*EF‐1*α) was used as an endogenous control, and reactions were performed in triplicate. PCR conditions consisted of an initial denaturation step at 94°C for 10 min, followed by 40 cycles of 94°C for 15 s and 60°C for 30 s.

Expression analysis of cotton genes encoding fatty acid amide hydrolases (FAAHs) was carried out using 4‐wk old seedlings of *G. hirsutum* cv Junmian No. 1. Plants were inoculated with a 1 × 10^7^ conidia ml^−1^ suspension of *V. dahliae* or fed with 20 mM *N*‐acylethanolamine (NAE 12:0). The seedling roots were harvested in three independent replicates at specific times after inoculation and then flash‐frozen in liquid N_2_ and stored at −80°C until use. The expression of eight cotton *FAAH* gene family members (*GhFAAH1*–*GhFAAH8*) were quantified by RT‐qPCR. The cotton *18S* gene was used as an internal control to normalize the variance among samples.

### D phenotype and virulence assays of *V. dahliae* secondary metabolites on cotton

Secondary metabolites were extracted from culture suspensions of strains as follows. Three independent transformants of each strain were cultured in 200 ml Czapek–Dox medium without antibiotics at 25°C in a shaking incubator at 180 rpm for 5 d. The liquid cultures were centrifuged at 21 000 ***g*** at 4°C for 20 min and the supernatants were filtered through a cellulose acetate filter (0.45 μm) and then cleaned by liquid–liquid extraction using 250 ml of chloroform : acetonitrile (1 : 1 v/v) twice. Next, the lower layer (organic solvent) was collected in a round‐bottom flask and concentrated to near dryness using a rotary evaporator at 35°C. The residues of each transformant were reconstituted in 2.0 ml water and briefly centrifuged. The supernatants were collected for application on cotton seedlings. Cotton seedlings were evaluated for defoliation, wilt‐like symptoms, and vascular discoloration in the presence of secondary metabolites from the supernatants as follows. Roots were trimmed from 4‐wk‐old cotton seedlings and transferred into 150 μl of secondary metabolite suspension. After absorption, the seedlings were transferred into water and maintained in a glasshouse at 25°C under a 14 h : 10 h light : dark with 75% humidity. The D phenotype and disease symptoms induced by secondary metabolites were observed 2 wk after treatment.

### NAE quantification and its role in defoliation

NAE 12:0 in culture suspensions was identified and quantified by ultrahigh performance liquid chromatography–tandem mass spectrometry. The strains were cultured in 200 ml Czapek–Dox medium without antibiotics at 25°C in a shaking incubator at 180 rpm for 5 d. Liquid cultures were centrifuged at 21 000 ***g*** at 4°C for 20 min to collect the supernatant. Suspensions were filtered through a cellulose acetate filter (0.45 μm) and lyophilized at −20°C. Residues were reconstituted in 30 ml of acetonitrile, followed by addition of 4 g anhydrous magnesium sulfate and 1 g sodium chloride. The solution was vortexed at full speed for 5 min and centrifuged for 5 min at 2500 ***g***. The upper layer (acetonitrile) was collected in a round‐bottom flask and concentrated almost to dryness using a rotary evaporator (Yarong Machiners, Shanghai, China) at 35°C. The residues were reconstituted in 2 ml of acetone and filtered through 0.22 μm syringe filters for ultrahigh performance liquid chromatography–tandem mass spectrometry analysis.

Chromatographic separation was carried out using an Agilent 1290 LC system (Agilent Technologies, Santa Clara, CA, USA) consisting of a four‐channel online degasser, a standard binary pump, and an Agilent Poroshell120 EC‐C18 column (2.1 mm × 50 mm, 2.7 μm particle size). The mobile phase consisted of ultrapure water containing 0.1% formic acid (eluent A) and methanol (eluent B). The gradient elution program was 30% B at injection time, with linear increase to 90% B in 5.0 min, where it was maintained for 2.0 min before returning to the initial conditions of 30% B (70% A) in 4.0 min. The flow rate was 0.4 ml min^−1^, and all compounds were eluted within 11.0 min. The temperature of the sample vial holder was set at 5°C, and the column temperature was maintained at 40°C to decrease viscosity. The injected volume was 1 μl.

An Agilent 6495 triple quadrupole mass spectrometer equipped with a conventional electrospray ionization source was used to quantify the three compounds of interest. N_2_ (99.95%) and argon (99.99%) were used as the nebulizer gas and the collision gas, respectively, and the pressure in the T‐wave cell was 3.2 × 10^−5^ MPa. The positive electrospray ionization mode and multiple reaction monitoring were used for the detection of the three compounds, and the tandem mass spectrometry conditions were optimized for the target compounds. Typical conditions were: source temperature, 200°C; capillary voltage, 3.0 kV; and desolvation temperature, 370°C. A cone gas flow of 50 l h^−1^ and a desolvation gas flow of 600 l h^−1^ were used. All other mass spectrometry parameters were optimized individually for each target compound, and the optimized parameters are listed later. masshunter software (Agilent) was used to collect and analyze the data.

### Defoliation function of NAE 12:0 on cotton plants

Four‐week‐old cotton plants were inoculated with a 1 × 10^7^ conidia ml^−1^ suspension of strain VDG78. One week later, 5 ml of 20 mM NAE 12:0 was fed as nutrition to the cotton seedlings. Plants fed with sterile distilled water or NAEs and plants inoculated only with Vd991 strain served as negative and positive controls, respectively. The D or ND phenotype of cotton plants was determined 10 d later.

## Results

### Population analyses reveal the presence of LS region G‐LSR2 only in the D pathotype strains of *V. dahliae*

Through comparative genomics of *V. dahliae* strains JR2 and VdLs.17, we previously showed that the lineage‐specific region G‐LSR2 encodes host‐specific adaptation factors, explaining the high virulence of Vd991 on cotton (Chen *et al*., 2018). Highly virulent strains generally cause defoliation in cotton, suggesting that G‐LSR2 itself may be associated with the defoliation function. Characterization of isolates from cotton with D and ND genetic markers indicated that the Vd991 genome contains the D marker but lacks the ND marker. The inverse is true for the genomes of JR2 and VdLs.17, which contain only the ND marker (Fig. S1a). Cotton plants (*G. hirsutum* cv Junmian No. 1) displayed the D phenotype following inoculation with Vd991, but not with JR2 or VdLs.17 (Fig. S1b). Fungal biomass *in planta* and vascular discoloration were significantly higher with Vd991, in contrast to either JR2 or VdLs.17 (Fig. S1c,d).

**Figure 1:**
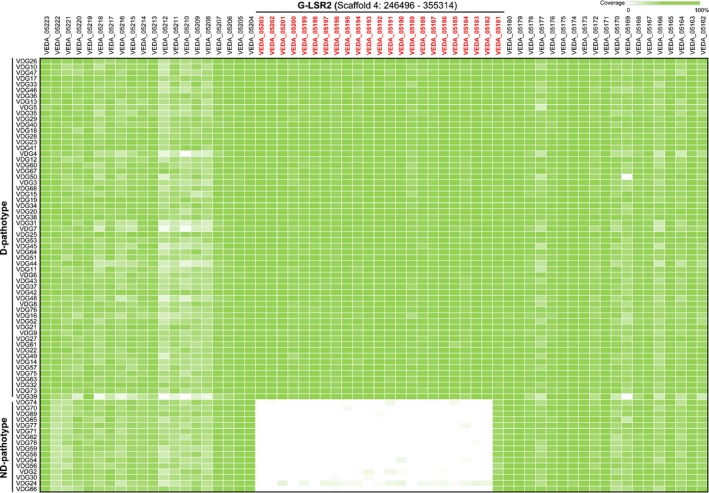
Sequenced read mapping comparisons between defoliating (D) and nondefoliating (ND) *Verticillium dahliae* isolates with the lineage‐specific genomic region, G‐LSR2. Whole‐genome resequencing isolates were collected from cotton in China. The D and ND pathotypes were determined by PCR amplification using D and ND markers (Pérez‐Artéz *et al*., 2000), revealing 59 and 16 isolates, respectively. Filtered reads were mapped to the *V. dahliae* Vd991G‐LSR2 reference sequence and two flanking genomic regions, each of which encodes an additional 20 genes. SOAP denovo (v.1.04) was used with the haploid model and default parameters for calculating the mapping coverage for protein coding genes. The gene accession numbers (red) represent individual genes in G‐LSR2.

To investigate the association between G‐LSR2 and the D pathotype, we performed whole‐genome resequencing of 75 isolates of *V. dahliae*. PCR data using D‐ and ND‐specific primer sets revealed 59 D isolates and 16 ND isolates (Table S2). PCR genotypes were validated by assessing the phenotypes of a subset of arbitrarily chosen three D and ND phenotypes (Fig. S2a,b). Additionally, *c*. 4 million paired‐end Illumina reads (PE = 90 bp) were generated for resequencing isolates for comparing G‐LSR2 between strains. Reads were mapped to the reference genes from G‐LSR2 and two flanking genomic regions that both harbor an additional 20 genes. As expected, with the exception of one gene (*VEDA_05181*) in G‐LSR2, sequencing reads from all 59 D isolates were mapped to 22 genes encoded by G‐LSR2, whereas sequence reads from the 16 ND isolates did not map with high significance (Fig. 1). The coverage depth and breadth of coverage of each gene in G‐LSR2 from the D isolates suggested the sequence data in this region were reliable (Table S3). Subsequent mapping of the total reads to G‐LSR2 flanking sequences revealed their presence in all the D and ND isolates (Fig. 1). These data suggest that G‐LSR2 is an LS region present only in the D isolates from cotton.

### Seven genes in G‐LSR2 confer the D function in *V. dahliae*

**Figure 2:**
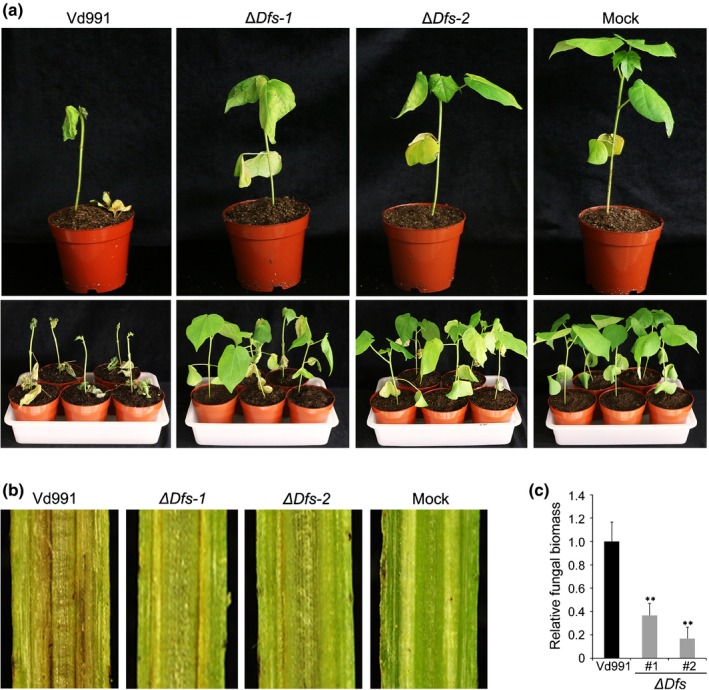
Seven genes encoded by lineage‐specific region of *VdDf*s in *Verticillum dahliae* are required for the defoliating phenotype on cotton. (a) Pathogenicity assay for investigating the role of the genomic region *VdDf*s in defoliation by *V. dahliae*. Four‐week‐old susceptible cotton plants (*Gossypium hirsutum* cv Junmian No. 1) were inoculated with a 1 × 10^7^ conidia ml^−1^ conidial suspension of wild‐type *V. dahliae* Vd991, knockout mutants of the entire *VdDf*s region (Δ*Dfs‐1* and Δ*Dfs‐2*, constructed by the homologous recombination that has already been described in Chen *et al.,* 2018), or sterile water using a standard root‐dip method. There were three independent replicates consisting of 12 plants. Plants were photographed 3 wk post‐inoculation. (b) Vascular discoloration in cotton after inoculation with gene deletion mutants at 4 wk post‐inoculation. Uninoculated plants were used as controls. (c) *In planta* fungal biomass development of the *VdDf*s knockout mutants (Δ*Dfs‐1* and Δ*Dfs‐2*) in inoculated cotton roots, and quantified by quantitative PCR. Error bars represent SE. Statistical significance has been represented (according to unpaired Student's *t*‐tests): **, *P *<* *0.01.

Seven genes within G‐LSR2 (*VdDf1* – *VdDf7*) contribute to the adaptation of *V. dahliae* Vd991 to cotton (Chen *et al*., 2018). Whether *VdDf*s are linked to the D phenotype in cotton was evaluated. Almost complete defoliation was observed 3–4 wk after inoculation with the wild‐type strain Vd991 (Figs 2a, S3). However, the D phenotype was not observed after inoculation with the *VdDf*s deletion mutant *∆Dfs* (as in Chen *et al*. (2018)) (Figs 2a, S3). The *∆Dfs* strains caused less vascular discoloration than the wild‐type strain Vd991 (Fig. 2b), and contained significantly less fungal biomass in cotton roots (< 40%) compared with the biomass in plants inoculated with the wild‐type strain Vd991 (Fig. 2c). These results suggested that the *VdDf*s contribute to the D phenotype.

### *VdDf5* and *VdDf6* confer the D phenotype in the LS region G‐LSR2

**Figure 3:**
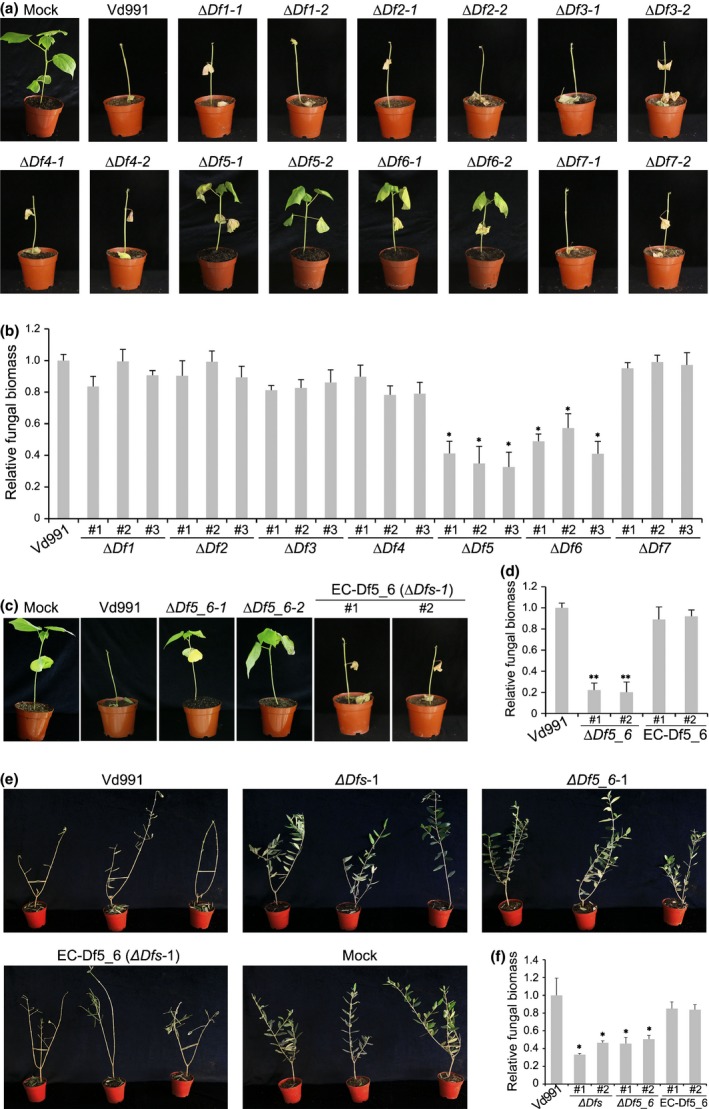
Identification of functional genes in *VdDf*s from *Verticillium dahliae* involved in the defoliating phenotype during infection on cotton. (a) Discovery of the genes involved in the defoliating phenotype using single‐gene deletion mutants. Plants of four‐week‐old cotton (*G. hirsutum* cv Junmian No.1) were root‐dip‐inoculated with single gene knockout mutants of each of the seven genes (Δ*Df1* to Δ*Df7*). A conidial suspension of 1 × 10^7^ conidia ml^−1^ for each mutant strain was prepared and inoculated. Three replicates consisting of 12 cotton plants each were included for each experiment. The wild‐type *V. dahliae* Vd991 and sterile water (Mock) treatments were used as positive and negative controls, respectively. Plants were maintained at 25°C in a glasshouse under a 14 h : 10 h, light : dark cycle. The defoliation phenotypes were investigated 4 wk after inoculation. (b) Quantitative PCR of *in planta* fungal biomass development of single gene knockout mutants. Error bars represent SE. (c) Assessment of the defoliating role of genes *VdDf5* and *VdDf6* together in *V. dahliae*. Four‐week‐old cotton plants were inoculated with a 1 × 10^7^ conidia ml^−1^ conidial suspension using a root‐dip method. Plants were inoculated with two independent targeted gene deletions of *VdDf5* and *VdDf6* (Δ*Df5_6‐1* and Δ*Df5_6‐2*), two transformants with reintroduced *VdDf5* and *VdDf6* genes into the *VdDf*s mutant background, or the wild‐type. Plants treated with sterile water were used as control (Mock). Three independent replicates were performed, each consisting of 12 plants. The defoliating phenotype was investigated 4 wk after inoculation. (d) Quantitative PCR analyses of fungal biomass development of *VdDf5* and *VdDf6* knockout mutants and corresponding ectopic transformants in cotton. Error bars represent SE. (e) The defoliating phenotype of strains (Vd991, *ΔDfs*‐1, *ΔDf5_6*‐1, EC‐Df5_6 (*ΔDfs*‐1)) on olive plants. The defoliating/nondefoliating phenotype of different strains was assessed using 4‐wk‐old olive plants 3 wk after root‐dip inoculation with a conidial suspension (1 × 10^7^ conidia ml^−1^). There were three independent replicates, each consisting of 12 plants. (f) Quantitative PCR analyses of *in planta* fungal biomass of different strains in olive. Error bars represent SE. Statistical significance has been represented (according to unpaired Student's *t*‐tests): *, *P *<* *0.05; **, *P *<* *0.01.

Single‐gene knockout mutants of genes in *VdDf*s were generated by homologous recombination (Fig. S4a–g). The virulence assays showed that deletion of *VdDf5* or *VdDf6* in the Vd991 background resulted in loss of the D phenotype in cotton at 4 wk after inoculation (Fig. 3a). Cotton plants inoculated with *∆Df5* and *∆Df6* strains displayed less vascular discoloration and fungal biomass, compared with other gene deletion strains and wild‐type (Figs 3b, S5a). Transcript abundances of the *VdDf*s genes were highly upregulated at 24 h post‐inoculation in cotton (Chen *et al*., 2018). Expression analysis of *VdDf1*–*VdDf7* revealed that the transcript levels, except for *VdDf3* and *VdDf4*, were significantly upregulated at 0.5–7 d after inoculation of cotton, especially with *VdDf5* and *VdDf6*, which were strongly upregulated (up to 20‐fold change) 2 d after inoculation (Fig. S6). These results suggested that *VdDf5* and *VdDf6* from *VdDf*s are the central genes involved in conferring the D phenotype. To confirm this, targeted replacement of *VdDf5* and *VdDf6* together was performed (Fig. S4h). Remarkably, the two independent *VdDf5* and *VdDf6* double deletion mutants (*∆Df5_6‐1* and *∆Df5_6‐2*) lost the ability to defoliate cotton (Figs 3c, S5b). Simultaneous complementation of *VdDf5* and *VdDf6* (*VdDf5_6*) in the *∆Dfs* strains resulted in the restoration of the defoliation phenotype (Figs 3c, S7a). Degrees of vascular discoloration and fungal biomass supported that *VdDf5* and *VdDf6* contribute to virulence on cotton (Figs 3d, S5b).

The D phenotype was evaluated on olive and okra, two other hosts that also exhibit defoliation of infected plants (Jiménez‐Díaz *et al*., 2006; Korolev *et al*., 2008). Both *∆VdDf*s and *∆VdDf5_6* lost the D phenotype on olive and okra, but this was recovered following the restoration of only *VdDf5* and *VdDf6* in the *VdDf*s deletion strains (Figs 3e, S8a). Fungal biomass levels also supported these observations (Figs 3f, S8b). Thus, only *VdDf5* and *VdDf6* confer the D phenotype in the three plant hosts.

### Sequence divergence of defoliation genes *VdDf5* and *VdDf6* following transfer from *F. oxysporum* f. sp. *vasinfectum*

**Figure 4:**
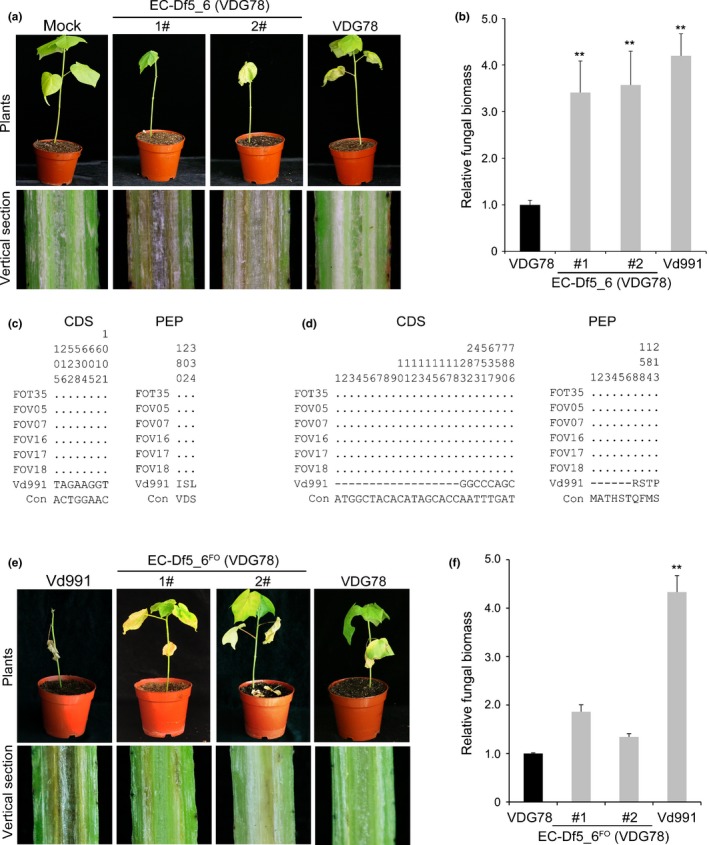
*VdDf*s genes *VdDf5* and *VdDf6* are required for defoliation and have diverged since horizontal transfer from *Fusarium oxysporum* f. sp. *vasinfectum* to *Verticillium dahliae*. (a) Cotton defoliation phenotype assays of *V. dahliae* after transfer of genes *VdDf5* and *VdDf6* together into the nondefoliating genotype background of isolate VDG78. Four‐week‐old cotton plants (*Gossypium hirsutum* cv Junmian No. 1) were root‐dip inoculated with a 1 × 10^7^ conidia ml^−1^ conidial suspension of the ectopic transformants of strain VDG78 that was the recipient of genes *VdDf5* and *VdDf6* together. There were three independent replicates, each consisting of 12 plants. Whole plants and shoot vertical sections were photographed 4 wk after inoculation. (b) Quantitative PCR analyses of *in planta* fungal biomass development of the wild‐type and ectopic transformants at 4 wk after inoculation. Partial sequence polymorphisms of (c) *VdDf5* and (d) *VdDf6* compared with homologous gene and peptide sequences from *F. oxysporum* f. sp. *vasinfectum* NRRL 25433. clustalX 1.83 was used for multiple sequence alignments. Only residues that deviate from the reference sequences are shown in the alignment; deletions are indicated by dashes; CDS, coding sequence; PEP, protein sequence. The polymorphic positions are written vertically; that is, the first polymorphism occurs at position 105 of the coding sequence. (e) Assessment of defoliation phenotypes after transfer of homologues of *VdDf5* and *VdDf6* from *F. oxysporum* f. sp. *vasinfectum* to the nondefoliating strain VDG78. Four‐week‐old cotton plants (*G. hirsutum* cv Junmian No. 1) were root‐dip inoculated with a 1 × 10^7^ conidia ml^−1^ conidial suspension of two independent ectopic transformants (EC‐Df5_6^FO^). Experiments comprised three replicates consisting of 12 cotton plants each. The wild‐type *V. dahliae* strain Vd991 and sterile water (Mock) were used as positive and negative controls, respectively. Plants were maintained in a glasshouse at 25°C under a 14 h : 10 h, light : dark cycle. The disease phenotypes of whole plants and shoot vertical sections were assessed at 4 wk after inoculation. (f) Quantitative PCR analyses of fungal biomass development of ectopic transformants co‐overexpressing the *F. oxysporum* f. sp. *vasinfectum VdDf5* and *VdDf6* homologues. Error bars represent SE. Statistical significance has been represented (according to unpaired Student's *t*‐tests): **, *P *<* *0.01.

To investigate whether *VdDf5* and *VdDf6* confer the D phenotype in different genetic backgrounds, ectopic mutants expressing *VdDf5* and *VdDf6* together were generated in the ND strains VDG78 (from cotton) and VdLs.17 (from lettuce) (Fig. S7b,c). Remarkably, the ND strain VDG78 gained the ability to cause the D phenotype following the simultaneous introduction of *VdDf5* and *VdDf6* (Fig. 4a), as well as to cause severe vascular discoloration in inoculated plants that also harbored higher fungal biomass than those inoculated with the wild‐type ND strain (Fig. 4a,b). The introduction of *VdDf5* and *VdDf6* together into VdLs.17 resulted in similar disease phenotypes (Fig. S9a,b). However, the defoliation symptoms caused by these ND transformants following the introduction of *VdDf5* and *VdDf6* were weaker than symptoms caused by the D strain Vd991 (Figs 4a,b, S9a,b).

Previous studies (Chen *et al*., 2018) indicated that G‐LSR2 was likely horizontally transferred from *F. oxysporum* f. sp. *vasinfectum* and encodes genes that may explain the dominant adaptation to cotton. To explore the molecular evolution of *VdDf*s following transfer from *F. oxysporum* f. sp*. vasinfectum*, the homologues of *VdDf*s in *F. oxysporum* f. sp. *vasinfectum* were aligned with *V. dahliae* sequences. This revealed several polymorphisms, including start codon changes, nonsynonymous single nucleotide polymorphisms, and truncation variations (Fig. S10). The *VdDf5* and its homologous gene in *F. oxysporum Df5* ^*Fo*^ alignments revealed three nonsynonymous mutations (Fig. 4c), and eight single nucleotide polymorphisms resulting in four nonsynonymous changes in *VdDf6* and its homologous gene in *F. oxysporum Df6* ^*Fo*^, and an 18 bp truncation resulting in a different start codon (Fig. 4d).

Next, we assessed the function of the *VdDf5* and *VdDf6* orthologues from *F. oxysporum* f. sp. *vasinfectum* (*Df5* ^*FO*^ and *Df6* ^*FO*^, *Df5_6* ^*FO*^) by ectopic expression in a cotton ND isolate. RT‐qPCR analysis showed that the *Df5* ^*FO*^ and *Df6* ^*FO*^ were successfully expressed in the ectopic transformants and the expression levels were similar to *VdDf5* and *VdDf6* from strain Vd991 during infection of cotton (Fig. S11). Unlike *VdDf5* and *VdDf6*, which conferred the D phenotype to *V. dahliae* (Figs 4a,b, S9a,b), *Df5_6* ^*FO*^ expression did not confer either the D phenotype to the cotton ND isolate (VDG78) (Fig. 4e) or higher virulence (Fig. 4f). These results suggested that amino acid substitutions in *VdDf5* and *VdDf6* resulting in higher virulence/defoliation were selected for in *V. dahliae* following the horizontal transfer from *F. oxysporum* f. sp. *vasinfectum*.

### G‐LSR2 is a secondary metabolite gene cluster controlling biosynthesis of a compound with D activity

**Figure 5:**
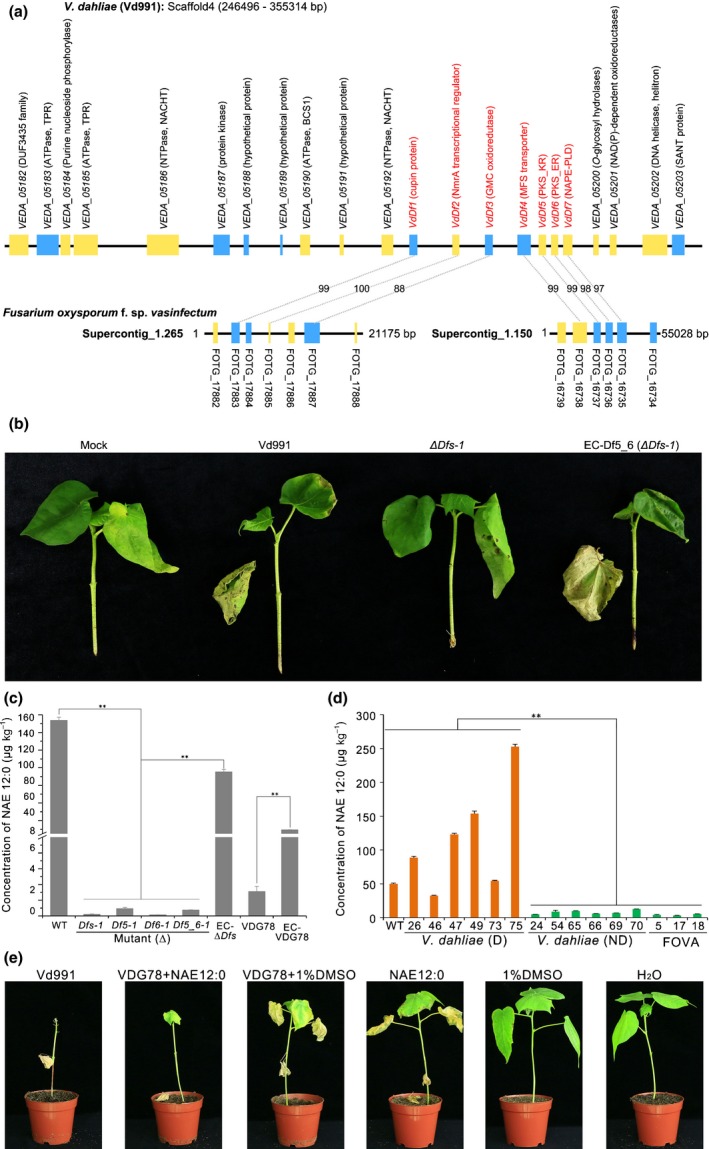
The genomic region G‐LSR2 of *Verticillium dahliae* strain Vd991 encodes a secondary metabolite gene cluster in which *VdDf5* and *VdDf6* play crucial roles in the defoliation phenotype. (a) Gene synteny analysis of the protein‐coding genes in G‐LSR2 between *V. dahliae* Vd991 and *Fusarium oxysporum* f. sp. *vasinfectum* NRRL 25433. Genes in G‐LSR2 and two assembly sequences (supercontig_1.265 and supercontig_1.150) were drawn in blocks according to start and stop codon positions. Blocks in blue and yellow colors represent genes encoded by sense and antisense strands, respectively. The gray dotted lines indicate seven syntenic genes between *VdDf*s in *V. dahliae* and assembly sequences in *Fusarium*. Numerical values are the identities of peptide sequences. Prediction of conserved domains of 22 protein‐coding genes in G‐LSR2. Conserved domains were predicted by the SMART procedure (http://smart.embl-heidelberg.de/), signal peptides and transmembrane domains were predicted by signalP4.1 and tmhmm2.0 procedures, respectively. TPR, tetratricopeptide repeat; NACHT, nucleoside triphosphatase; BSC1, mitochondrial chaperone BCS1; NmrA, the negative transcriptional regulator involved in the post‐translational modification of transcription factor AreA; GMC oxidoreductases, glucose–methanol–choline oxidoreductases; MFS transporter, major facilitator superfamily transporter; PKS_KR, polyketide biosynthase_ketoacylreductase; PKS_ER, polyketide biosynthase_enoylreductase; NAPE‐PLD:* N*‐acyl‐phosphatidylethanolamine‐hydrolyzing phospholipase D; SANT, switching‐defective protein 3 (Swi3), adaptor 2 (Ada2), nuclear receptor co‐repressor (N‐CoR), transcription factor (TF)IIIB. (b) Toxicity of secondary metabolites mediated by *VdDf*s. After cotton seedlings absorbed 150 μl of secondary metabolite supernatants from strains Vd991, Δ*Dfs‐1* and EC‐Df5_6 (Δ*Dfs‐1*), seedlings were transferred to water and maintained in a glasshouse at 25°C under a 14 h : 10 h, light : dark cycle with 75% humidity. Photographs were taken 2 wk after treatment. (c) Quantitative analysis of *N*‐acylethanolamine (NAE) 12:0 extracted from different strains (Vd991, Δ*Dfs‐1*, Δ*Df5‐1*, Δ*Df6‐1*, Δ*Df5_6‐1* and EC‐Df5_6 (Δ*Dfs‐1*); VDG78 and EC‐VDG78 (introduced VdDf5 and VdDf6 into ND strain VDG78)) by ultrahigh performance liquid chromatography–tandem mass spectrometry (UHPLC‐MS/MS). Error bars represent SE; statistical significance has been represented (according to unpaired Student's *t*‐tests): **, *P *<* *0.01. WT, wild‐type. (d) Quantitative analysis of NAE 12:0 in *V. dahliae* defoliating (D) and nondefoliating (ND) strains, and *F. oxysporum* f.sp. *vasinfectum* strains (labeled FOVA) extracts by UHPLC‐MS/MS. Error bars represent SE, statistical significance has been represented (according to unpaired Student's *t*‐tests): **, *P *<* *0.01. (e) Defoliation activity of NAE 12:0 on cotton plants. Four‐week‐old cotton plants were inoculated with the conidial of strain VDG78, using a root‐dip method. There were two independent replicates, each consisting of 12 plants. One week later, NAE 12:0 was fed on cotton roots at a concentration of 20 mM. Photographs were taken 5 d later. DMSO, dimethyl sulfoxide.

The annotation of 22 genes encoded by G‐LSR2 predicted a variety of functions. Twelve genes were homologous to *F. oxysporum* f. sp*. vasinfectum* stress response genes (Table S4). *VdDf5* and *VdDf6* share homology with polyketide synthases, whereas *VdDf1* and *VdDf3* are oxidoreductase homologues, *VdDf2* encodes an NmrA transcriptional regulator, and *VdDf4* and *VdDf7* share homology with a major facilitator superfamily gene and an *N*‐acylphosphatidylethanolamine‐hydrolyzing phospholipase D (NAPE‐PLD), respectively (Fig. 5a). Therefore, G‐LSR2 is likely a secondary metabolism gene cluster, which characteristically encodes polyketide synthases, oxidoreductases, transporters, transcriptional regulators, and hydrolases (Yu & Keller, 2005; Brakhage, 2013). Also, at least six protein‐coding genes in this cluster share a conserved NACHT and TPR domain or a P‐loop nucleoside triphosphate hydrolase (P‐loop NTPase) domain, such as the genes *VDEA_05186*,* VDEA_05185*, and *VDEA_05183* (Fig. 5a), which are often involved in host–pathogen interactions by transcriptional regulation (Leipe *et al*., 2004).

To investigate whether genes in G‐LSR2 function in secondary metabolite production and secretion that leads to defoliation, the toxicity of putative secondary metabolites from G‐LSR2 wild‐type and mutant *V. dahliae* strains were assayed on plants. Cotton seedlings were treated with secondary metabolites extracted from culture supernatants from the wild‐type and the Δ*Dfs* strain, and the Δ*Dfs* strain simultaneously complemented with *VdDf5* and *VdDf6*. As expected, cotton leaves displayed wilting, chlorosis, and began to defoliate 2 wk after treatment with the culture supernatant extracted from wild‐type strains *in vitro*, but showed no D phenotype following treatment with the *ΔDfs* culture supernatant (Fig. 5b). Treatment with supernatant collected from the *VdDf5‐6* complemented‐*ΔDfs* strain also resulted in defoliation (Fig. 5b).

Interestingly, *VdDf7* shares homology to a gene encoding NAPE‐PLD, which hydrolyzes *N*‐acylphosphatidylethanolamines (NAPEs) to produce NAEs. Intriguingly, NAEs are known to affect plants through ABA signaling (Blancaflor *et al*., 2014), impacting leaf senescence and stomatal closure (Murata *et al*., 2015). Quantification of three NAEs (NAE 12:0, NAE 14:0, and NAE 16:0) showed that the concentration of NAE 12:0 was significantly lower (*c*. 100‐fold less) in culture suspensions of the deletion strains (*ΔDfs*‐*1*,* ∆Df5‐1*,* ∆Df6‐1*, and *∆Df5_6‐1*) relative to the wild‐type, and NAE content was restored after the simultaneous re‐introduction of *VdDf5* and *VdDf6* into the *ΔDfs* mutant and ND strain VDG78 (Fig. 5c). Furthermore, the concentrations of NAE 12:0 were significantly lower in ND strains than in D strains in culture (Fig. 5d). However, the concentration of NAE 16:0 between D and ND strains was not statistically significant (Fig. S12). In contrast, the concentration of NAE 14:0 was significantly (*P < *0.05) higher in the ND strains than in D strains (Fig. S12). Correspondingly, cotton plants inoculated with the ND isolate VDG78 also displayed the D phenotype when combined with the NAE 12:0 treatment (Fig. 5e). Therefore, *VdDf5* and *VdDf6* within the G‐LSR2 cluster are crucial to the NAE 12:0 biosynthesis.

### NAEs are involved in defoliation by interfering with normal NAE metabolism in cotton plants

**Figure 6:**
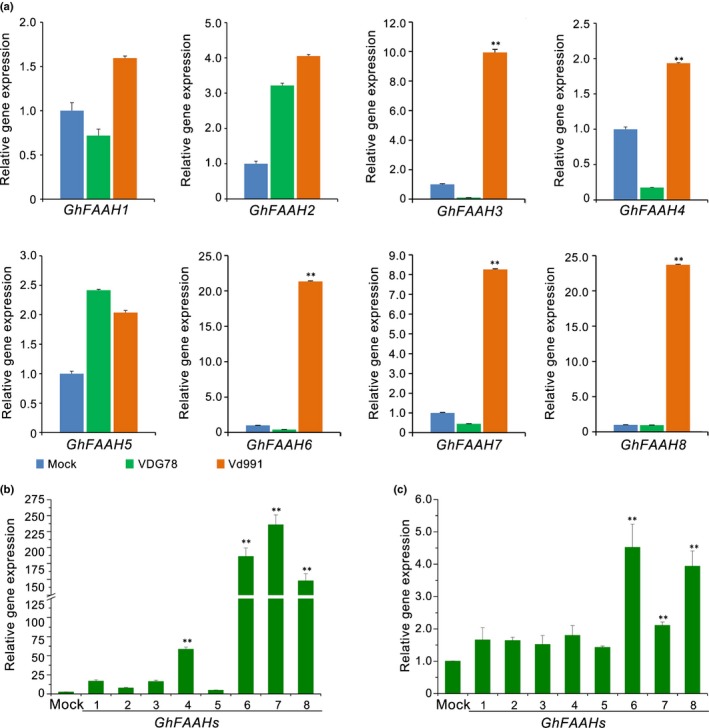
Detection of the cotton fatty acid amide hydrolase (FAAH) gene family members’ (*GhFAAH*s’) responses to defoliating (D). (a) Expression analysis of *GhFAAH*s during *Verticillium dahliae* infection on cotton roots. Four‐week‐old cotton plants (*Gossypium hirsutum* cv Junmian No. 1) were root‐dip inoculated with *V. dahliae* D strain Vd991 or nondefoliating (ND) strain VDG78 and harvested at 5 d post‐inoculation. Reverse transcription quantitative PCR was performed to determine the relative expression levels of *GhFAAH*s using the cotton *18S* gene as a reference (set as 1.0) and compared with expression levels of *GhFAAH*s observed in uninoculated cotton treated with distilled water. (b) Expression analysis of *GhFAAH*s by feeding *N*‐acylethanolamine (NAE) 12:0 on cotton roots. Four‐week‐old cotton plants were root‐dip fed with NAE 12:0 and the roots were harvested at 48 h post‐treatment. The relative expression levels of *GhFAAH*s’ responses to NAE 12:0 were compared with the treatment with distilled water (set as 1.0). (c) Expression analysis of *GhFAAH*s by treating with culture filtrate on cotton roots. Four‐week‐old cotton plants were treating with culture filtrate of ND strain VDG78 and D strain Vd991, the roots were harvested at 48 h post‐treatment. The relative expression levels of *GhFAAH*s’ responses to the culture filter of D strain Vd991 were compared with the treatment with the culture filter of ND strain VDG78 (set as 1.0). Error bars represent SE; statistical significance has been represented (according to unpaired Student's *t*‐tests): **, *P *<* *0.01.

NAEs are a class of bioactive lipids, and FAAH is one of the enzymes responsible for degrading NAEs to fatty acid amide and ethanolamine in plants (Chapman, 2004). The annotation of conserved domains by interproscan previously revealed that no FAAH (InterPro ID: IPR030560) enzymes were present in the set of protein coding genes in *V. dahliae* Vd991 (Chen *et al*., 2018). Since *V. dahliae* lacks FAAH, potentially resulting in the inability to catabolize NAEs, the function of NAEs in *V. dahliae* may be related strictly to pathogenicity. Interestingly, RT‐qPCR analysis of the eight cotton *FAAH* gene family members (*GhFAAH1*–*GhFAAH8*) revealed that the transcript levels of *GhFAAH3*,* GhFAAH6*,* GhFAAH7*, and *GhFAAH8* were significantly upregulated (*c*. 8‐ to 23‐fold) in susceptible cotton at 5 d after inoculation with the D strain Vd991 compared with inoculation with ND strain VDG78 (Figs 6a, S13), which supported the idea that plant expression of *FAAH* occurs in response to the high concentration of NAE 12:0 transported from *V. dahliae*. *In vitro* assays demonstrated that the expression of *GhFAAH6*,* GhFAAH7*, and *GhFAAH8* was significantly higher at 48 h in NAE‐12:0‐fed cotton roots (Fig. 6b). Furthermore, the expression levels of cotton *FAAH* genes were relatively higher in plants treated with the culture filtrate from D strain (Vd991) than that from ND strain (VDG78) (Fig. 6c). Therefore, the secondary metabolite gene cluster (*VdDf*s) from *F. oxysporum* enables efficient NAE biosynthesis and perhaps also the transport into the host plant cell; cotton plant FAAHs were significantly upregulated in response to the high level of NAEs transported from the *V. dahliae* D strains (Fig. 6).

## Discussion

Even though *V. dahliae* is a well‐known wilt pathogen with a very broad host range, it is able to cause defoliation in only three hosts; namely, cotton, olive, and okra (Schnathorst & Mathre, 1966; Jiménez‐Díaz *et al*., 2006; Korolev *et al*., 2008). The reasons for this symptom dichotomy among the more than 200 hosts on which *V. dahliae* causes wilt has largely remained unresolved. In this study, we examined the molecular and genetic underpinnings of defoliation based on insights gleaned from our previous work (Chen *et al*., 2018). Earlier, we showed that a seven‐gene sequence within the flexible genomic DNA region G‐LSR2 of *V. dahliae* Vd991, acquired through horizontal transfer from *F. oxysporum* f. sp*. vasinfectum*, conferred higher virulence and adaptation towards cotton (Chen *et al*., 2018). Of the seven genes (*VdDf1*–*VdDf7*) within G‐LSR2, we have demonstrated that *VdDf5* and *VdDf6* are critical for the D phenotype. Interestingly, these two genes appear to have undergone sequence evolution following the transfer from *F. oxysporum* f. sp*. vasinfectum*. G‐LSR2 has the characteristics of a secondary metabolism gene cluster. *VdDf5* and *VdDf6* encode polyketide synthase homologues that appear to function in conjunction with oxidoreductases, transporters, and a transcriptional regulator in the biosynthesis, metabolism, and transport of NAE 12:0, a compound that can induce the D phenotype.

Although molecular markers for identifying the D and ND pathotypes have been available for some time (Pérez‐Artéz *et al*., 2000), the functional genes associated with or linked to the D marker have not been understood. Interestingly, blast searches indicated that the sequence of the D marker displayed high identity to the G‐LSR2 border sequence (Scaffold 4: 354531–355476), specifically in the coding sequence of *VEDA_05203* (Fig. S14). In addition, population genomics of *V. dahliae* from cotton showed that the presence/absence of G‐LSR2 correlated precisely with the D and ND PCR markers (Pérez‐Artéz *et al*., 2000). Deletion of G‐LSR2 in D strains results in the ND phenotype on cotton, olive, and okra, providing strong genetic evidence that the LS region G‐LSR2 underlies the D phenotype in *V. dahliae*.

Acquisition of genes through horizontal gene transfer allows microbes to rapidly gain new capabilities and adapt to new and changing environments (Bonham *et al*., 2017). Various mechanisms have been described that result in the domestication of exogenous genetic material, including compensatory evolution, positive selection, and changes in gene expression (Wiedenbeck & Cohan, 2011). Comparative analyses of *VdDf1*–*VdDf7* nucleotide and encoded amino acid sequences relative to the donor sequences from *F. oxysporum* f. sp. *vasinfectum* (also a cotton wilt pathogen) revealed divergence, including nonsynonymous/synonymous mutations, and alterations in start or stop codon usage (Figs 4c,d, S10). Expression analysis showed that *Df6* ^*Fo*^ was significantly upregulated, but *Df5* ^*Fo*^ was not expressed (or at lower levels) during *F. oxysporum* f. sp*. vasinfectum* infection of cotton plants (Fig. S15). This suggested that *F. oxysporum* f. sp*. vasinfectum* was unable to cause defoliation owing to the ineffective expression of both *Df5* ^*Fo*^ and *Df6* ^*Fo*^ genes. Since the key genes *VdDf5* and *VdDf6* were transcribed and expressed stably and are critical to the defoliation phenotype, the *F. oxysporum* f. sp*. vasinfectum* homologues were insufficient to induce defoliation, even when *Df5* ^*Fo*^ and *Df6* ^*Fo*^ were successfully expressed in *V. dahliae* (Fig. S11). Furthermore, even though NAE 12:0 was also detected in *F. oxysporum* f. sp*. vasinfectum*, its concentration was representative of that displayed by ND strains and significantly lower than in D strains (Fig. 5d). These results suggested that *Df5* ^*Fo*^ and *Df6* ^*Fo*^ genes are functionally defective in *F. oxysporum* f. sp*. vasinfectum*. It therefore appears that genes in *VdDf*s underwent positive selection for virulence in *V. dahliae* following the horizontal transfer from *F. oxysporum* f. sp*. vasinfectum*.

Fungal secondary metabolites are extremely diverse and perform a range of functions, including iron acquisition, stress defense, and toxic assaults on living hosts, and so on (Macheleidt *et al*., 2016). Secondary metabolism genes are usually organized in clusters and typically encode polyketide and nonribosomal peptide synthases involved in the synthesis of a diverse array of compounds, such as terpenes and indole alkaloids (Brown *et al*., 1996; Kennedy *et al*., 1999; Yu & Keller, 2005; Brakhage, 2013) and melanin, as in *V. dahliae* (Wang *et al*., 2018). During infection, *V. dahliae* secretes a diverse range of effectors (de Jonge *et al*., 2012; Gui *et al*., 2017; Zhang *et al*., 2017) and secondary metabolites (Xiong *et al*., 2016; Zhang *et al*., 2016). Interestingly, secondary metabolite production mediated by genes in *VdDf*s can induce defoliation in the absence of the pathogen (Fig. 5b). The arrangement and functional annotation of genes in G‐LSR2 are characteristic of a fungal secondary metabolism cluster, which typically contains polyketide synthases, oxidoredutases, transporters, and transcriptional regulators (Brown *et al*., 1996; Kennedy *et al*., 1999). The proteins encoded by secondary metabolism gene clusters often contain the conserved NACHT, TPR, or P‐loop NTPase domains (Weijn *et al*., 2013) and share homology with protein domains encoded in G‐LSR2 (Fig. 5a). Furthermore, *VdDf1*–*VdDf7* were co‐expressed during infection of cotton (Fig. S6), as predicted for coordinately regulated genes that are located in the secondary metabolite clusters (Collemare *et al*., 2008) known to be vital for virulence.

Multiple secreted secondary metabolites are known to function in pathogenesis (Macheleidt *et al*., 2016). NAEs are bioactive lipids derived from the hydrolysis of the membrane phospholipid NAPE and are known to regulate a variety of physiological processes in animals and plants (Chapman *et al*., 1999; Chapman, 2004; Kilaru *et al*., 2012). In plants, the NAEs occur in low concentrations (nanograms per gram FW range in vegetative tissues of plants) and are involved in cytoskeleton modification, chloroplast degradation, root development, defense response, and interaction with ABA (Chapman, 2004). NAEs may also participate in signal transduction pathways that induce ABA‐responsive genes, block stomatal closure, and affect seed germination and seedling growth (Austin‐Brown & Chapman, 2002; Teaster *et al*., 2007; Kang *et al*., 2008; Keereetaweep *et al*., 2013; Blancaflor *et al*., 2014). Many of the physiological processes regulated by NAEs are consistent with the disease phenotypes induced by *V. dahliae*, including the inhibition of seedling growth (Veronese *et al*., 2003; Fradin & Thomma, 2006), alterations to the cytoskeleton in plants treated with Vd‐toxin extracted from the culture filtrate of strain Vd991 (Hu *et al*., 2014). Moreover, ABA is a key plant hormone mediating plant responses to environmental stresses, and promotes leaf abscission and senescence (Lim *et al*., 2007). Therefore, interactions between NAEs and ABA signaling could also contribute to leaf abscission and the defoliation phenotype. In our study, *VdDf*s played a critical role in NAE 12:0 production associated with *V. dahliae* D pathotype. NAE 12:0 also caused defoliation in plants treated a week after inoculation with the ND strain. Although NAE 14:0 had little role in defoliation, the metabolic equilibrium of NAEs (Chapman, 2004) resulted in NAE 14:0 being higher in ND strains than in D strains (Fig. S12a). This further confirmed that the production of NAE 12:0 by *V. dahliae* contributes to leaf abscission and the defoliation phenotype.

This study also demonstrated that *VdDf5* and *VdDf6* encode putative polyketide synthases likely involved in NAE 12:0 biosynthesis. Polyketide synthases play a critical role in the lipid metabolism, and NAPEs and NAEs are N‐containing lipids (Mohanty *et al*., 2011; Quadri, 2014; Miyazawa *et al*., 2015). Furthermore, *VdDf7* was predicted to encode NAPE‐PLD that hydrolyzes NAPEs to produce NAEs. High levels of NAE 12:0 observed in D strains caused leaf abscission, and cotton inoculated with the ND strain also displayed leaf abscission following treatment with the exogenous NAE 12:0, indicating that the high level of NAE 12:0 in cotton secreted by D *V. dahliae* strain causes leaf abscission. Furthermore, NAE 12:0, known to inhibit phospholipase Dα activity and block ABA‐induced stomatal closure (Austin‐Brown & Chapman, 2002), could also significantly facilitate pathogen infection of cotton. Functional annotation of the *V. dahliae* genome showed that a duplicate orthologue encoding NAPE‐PLD exists in the *V. dahliae* strain Vd991 (*VEDA_04083*), which was highly homologous to VdLs.17 (*VDAG_10085*) and JR2 (evm.model.contig44686.491). The apparent redundant function of two NAPE‐PLD genes in Vd991 may explain why the defoliation phenotype was not abolished after deletion of *VdDf7* (Fig. 3). Furthermore, the functional homologues of *VdDf*s (NmrA transcriptional regulator, major facilitator superfamily, etc.) are ubiquitous in *V. dahliae*, which likely provide the complementary functionality for NAE 12:0 biosynthesis by *VdDf5* and *VdDf6* in the ND strain (Fig. 4a). The production of NAE was restored following the simultaneous reintroduction of *VdDf5* and *VdDf6* into the *ΔDfs* mutant EC‐Df5_6(ΔDfs) or ND strains, suggesting that *VdDf5* and *VdDf6* are vital for NAE biosynthesis under the premise that NAPE‐PLD is involved in the hydrolysis of NAPE to NAE; but the exact function needs to be elucidated further.

**Figure 7:**
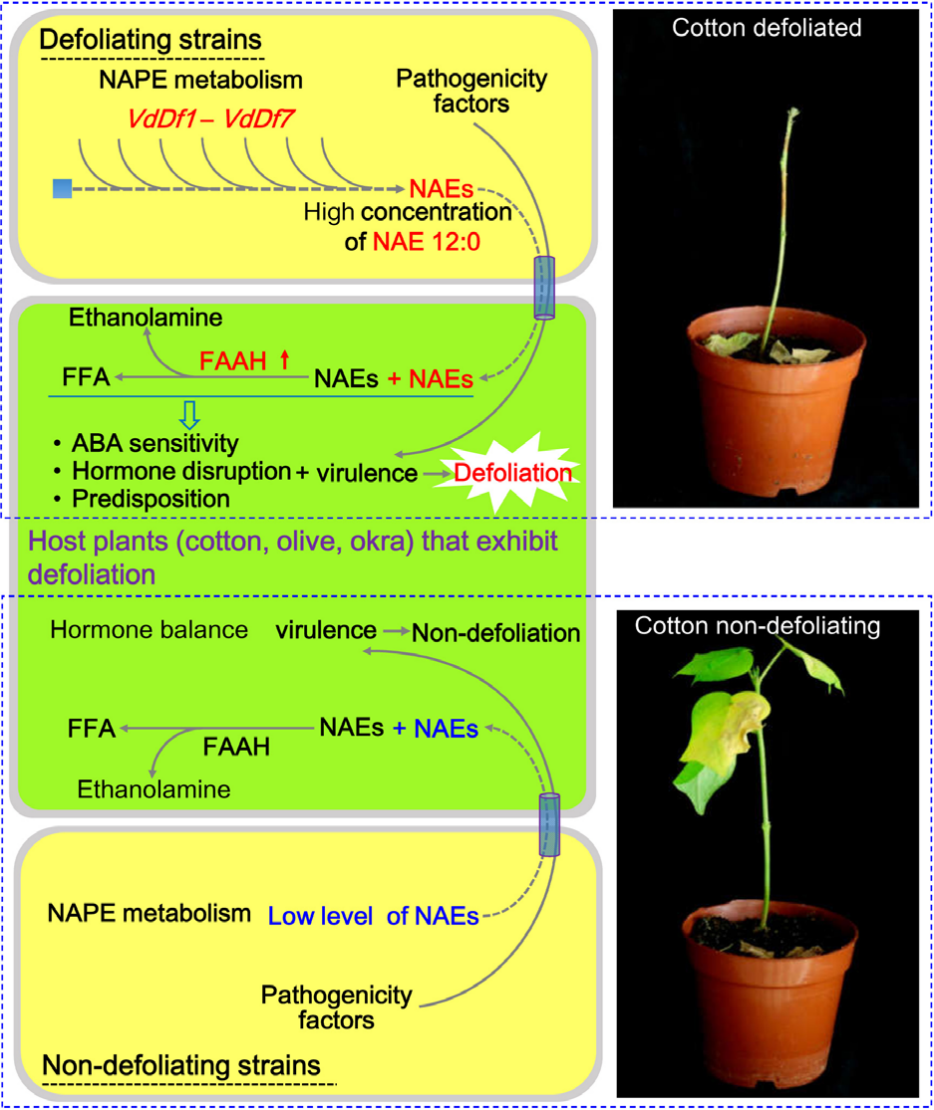
A hypothetical model of *N*‐acylethanolamines (NAEs) in the defoliation phenotype observed in certain *Verticillium dahliae*–cotton interactions. ABA, abscisic acid; FAAH, fatty acid amide hydrolase; FAA, fatty acid amide; FFA, free fatty acid; NAPE,* N*‐acylphosphatidylethanolamine. The red arrow on the side of FAAH indicates significantly upregulated expression; red, blue, and black fonts indicate the origin of NAE, either from a defoliating strain, a nondefoliating strain, or the host plant, respectively.

NAEs, comprising a family of functionally diverse signaling lipids in plants and animals, are transformed into free fatty acids and ethanolamine by FAAHs (Blancaflor *et al*., 2014). In plants, overexpression of FAAHs also disturbs the hormone balance, enhances ABA sensitivity, and compromises innate immunity (Blancaflor *et al*., 2014). Although the NAEs could be synthesized in microbes (Ellingson, 1980; Merkel *et al*., 2005), the transformation of NAEs by FAAH has not been reported in microbes. Also, in *V. dahliae* no conserved domains of FAAH were found among any of the encoded proteins in the genome (Chen *et al*., 2018). In Arabidopsis, the enzymes encoded by *At5g64440* (*AtFAAH*) and *At1g08980* (*AMI1*) have been shown to exhibit NAE hydrolytic activity (Shrestha *et al*., 2003). Interestingly, direct inoculation of cotton plants with the D strain or treatment with NAE 12:0 or treatment of the plants with the culture filtrate from the D strain uniformly resulted in the upregulation of *FAAH* genes (Fig. 6), suggesting that cotton increases the expression level of *FAAH* genes in response to high‐level NAEs that were putatively released into the plant from the D *V. dahliae* strains (Fig. 5c,d). The D strains displayed a high efficiency of NAE biosynthesis (Fig. 5d), which appeared to induce the overexpression of *FAAH* gene family members (Fig. 6) and likely disrupt the host plant native NAE metabolism. Previous research also found that the overexpression of *AtFAAH* in Arabidopsis affected salicylic acid and jasmonic acid production, rendering the host more sensitive to ABA and pathogen infection (Kang *et al*., 2008). Therefore, upregulation of *FAAH* genes may also increase cotton plant sensitivity to the ABA, disrupt hormone sensitivity, and predispose plants to pathogen infection accompanied by defoliation (Fig. 7).

In conclusion, our study provides strong evidence that the defoliation and high virulence of D pathotype is the result of secondary metabolite NAE 12:0, the biosynthesis of which is controlled by genes in the lineage‐specific region G‐LSR2 in *V. dahliae*.

## Author contributions

XFD, JYC and KVS conceived the study and designed all experiments, while JYC, DDZ, ZQK, LZ, DW, JQH and CL performed the data analysis and secondary metabolites identification, and RXL performed the additional secondary metabolites data of *F. oxysporum* in the revision stage. JW, DDZ, YJG, JJL, BLW, CMY and TGL performed the targeted gene deletion, ectopic expression analysis, and pathogenicity and D/ND phenotype identification. JYC, DPGS, RMB, SJK, DDZ, JLW and GYZ contributed to the writing of the manuscript. KVS conceptualized the study, reviewed the data, and edited the manuscript. All authors have read, commented, and approved the manuscript. D‐DZ, JW, DW, Z‐QK and LZ contributed equally to this work.

## Supporting information

Please note: Wiley Blackwell are not responsible for the content or functionality of any Supporting Information supplied by the authors. Any queries (other than missing material) should be directed to the *New Phytologist* Central Office.

**Fig. S1** Cotton defoliation and non‐defoliation phenotypes of *Verticillium dahliae* strains Vd991, JR2 and VdLs.17.
**Fig. S2** Defoliation/non‐defoliating phenotypes and PCR genotypes of different *Verticillium dahliae* isolates.
**Fig. S3** Cotton defoliation phenotype dynamics from one to four weeks following inoculation with genomic region G‐LSR2 deletion mutant *ΔDfs*.
**Fig. S4** Screening of gene deletion mutants.
**Fig. S5** Identification of functional genes encoded by *VdDfs* involved in vascular discoloration during infection of cotton by *Verticillium dahliae*.
**Fig. S6** Expression analysis of *VdDf1*–*VdDf7*, the seven G‐LSR2 genes during infection of cotton by *Verticillium dahliae*.
**Fig. S7** PCR verification of the transfer of two genes D*f5‐6* from the wild‐type *Verticillium dahliae* strain Vd991 to strains VdLs.17 and VDG78, and mutant background *ΔDfs‐1*, respectively.
**Fig. S8** Defoliation phenotypes on okra caused by different *Verticillium dahliae* strains.
**Fig. S9** The collaborative role of genes *Df5* and *Df6* in conferring the defoliation phenotype in *Verticillium dahliae* strain VdLs.17.
**Fig. S10** Comparison of the sequence divergence of *VdDfs* between *Verticillium dahliae* Vd991 and homologous genes from six different *Fusarium oxysporum* f. sp. *vasinfectum* strains.
**Fig. S11** Expression analysis of the *VdDf5* and *VdDf6* homologs, *Df5* ^*FO*^ and *Df6* ^*FO*^, during infection of cotton by the ectopic transformant.
**Fig. S12** Quantification of NAE 14:0 and NAE 16:0 extracted from different strains by UHPLC‐MS/MS.
**Fig. S13** Relative expression level analysis of the cotton *GhFAAH* genes in response to D strain Vd991.
**Fig. S14** Nucleotide alignment of the published defoliating marker sequence to the genomic region G‐LSR2 of *Verticillium dahliae* Vd991.
**Fig. S15** Expression analysis of the *VdDf5* and *VdDf6* homologs genes (*Df5* ^*FO*^ and *Df6* ^*FO*^) during infection of cotton by *F. oxysporum* f. sp. *vasinfectum*.
**Table S1** Information on isolates for which re‐sequenced genomes were obtained for this study.
**Table S2** Primers used in this study.
**Table S3** The coverage breadth and depth of resequenced isolates mapped to encoding genes in G‐LSR2.
**Table S4** Information of lineage‐specific genes in Vd991.

## Data Availability

The raw data of 75 *V. dahliae* strains have been deposited in GenBank under PRJNA171348 and the CNGB Nucleotide Sequence Archive (CNSA: https://db.cngb.org/cnsa; accession number CNP0000156). The homologous gene IDs of *GhFAAH1*–*GhFAAH8* in *Gossypium raimondii* was Gorai.004G133000.1, Gorai.005G091000.1, Gorai.007G067000.1, Gorai.008G018500.1, Gorai.010G111500.1, Gorai.011G117700.1, Gorai.012G03720 0.1, and Gorai.013G053300.1, respectively.
