# Supplementary material for: Population genomics demystifies the defoliation phenotype in the plant pathogen Verticillium dahliae
Source: New Phytol. 2019 Feb 25;222(2):1012–29. doi: 10.1111/nph.15672 (PMC6594092; doi:10.1111/nph.15672)
Supplement: Supplementary file 1 — Fig. S1 Cotton defoliation and non‐defoliation phenotypes of Verticillium dahliae strains Vd991, JR2 and VdLs.17. Fig. S2 Defoliation/non‐defoliating phenotypes and PCR genotypes of different Verticillium dahliae isolates. Fig. S3 Cotton defoliation phenotype dynamics from one to four weeks following inoculation with genomic region G‐LSR2 deletion mutant ΔDfs. Fig. S4 Screening of gene deletion mutants. Fig. S5 Identification of functional genes encoded by VdDfs involved in vascular discoloration during infection of cotton by Verticillium dahliae. Fig. S6 Expression analysis of VdDf1–VdDf7, the seven G‐LSR2 genes during infection of cotton by Verticillium dahliae. Fig. S7 PCR verification of the transfer of two genes Df5‐6 from the wild‐type Verticillium dahliae strain Vd991 to strains VdLs.17 and VDG78, and mutant background ΔDfs‐1, respectively. Fig. S8 Defoliation phenotypes on okra caused by different Verticillium dahliae strains. Fig. S9 The collaborative role of genes Df5 and Df6 in conferring the defoliation phenotype in Verticillium dahliae strain VdLs.17. Fig. S10 Comparison of the sequence divergence of VdDfs between Verticillium dahliae Vd991 and homologous genes from six different Fusarium oxysporum f. sp. vasinfectum strains. Fig. S11 Expression analysis of the VdDf5 and VdDf6 homologs, Df5 FO and Df6 FO, during infection of cotton by the ectopic transformant. Fig. S12 Quantification of NAE 14:0 and NAE 16:0 extracted from different strains by UHPLC‐MS/MS. Fig. S13 Relative expression level analysis of the cotton GhFAAH genes in response to D strain Vd991. Fig. S14 Nucleotide alignment of the published defoliating marker sequence to the genomic region G‐LSR2 of Verticillium dahliae Vd991. Fig. S15 Expression analysis of the VdDf5 and VdDf6 homologs genes (Df5 FO and Df6 FO) during infection of cotton by F. oxysporum f. sp. vasinfectum. Table S1 Information on isolates for which re‐sequenced genomes were obtained for this study. Table S2 Primers used in [file NPH-222-1012-s001.pdf]

New Phytologist **Supporting Information Figs S1–S15, Tables S1-S4**

Article title: **Population Genomics Demystifies the Defoliation Phenotype in the Plant Pathogen *Verticillium dahliae***

Authors: Dan-Dan Zhang[1#], Jie Wang[1#], Dan Wang[1#], Zhi-Qiang Kong[1#], Lei Zhou[1#], Geng-Yun Zhang[2], Yue-Jing Gui[1], Jun-Jiao Li[1], Jin-Qun Huang[2], Bao-Li Wang[1], Chun Liu[2], Chun-Mei Yin[1], Rui-Xing Li[1], Ting-Gang Li[1], Jin-Long Wang[3], Dylan P. G. Short[4], Steven J. Klosterman[5], Richard M. Bostock[6], Krishna V. Subbarao[4*], Jie-Yin Chen[1*], Xiao-Feng Dai[1*]

Article acceptance date: 18 December 2018

The following Supporting Information is available for this article:

Fig. S1. Cotton defoliation and non-defoliation phenotypes of *Verticillium dahliae* strains Vd991, JR2 and VdLs.17.

Fig. S2. Defoliation/non-defoliating phenotypes and PCR genotypes of different *Verticillium dahliae* isolates.

Fig. S3. Cotton defoliation phenotype dynamics from one to four weeks following inoculation with genomic region G-LSR2 deletion mutant *ΔDfs.*

Fig. S4. Screening of **gene deletion mutants.**

Fig. S5. Identification of functional genes encoded by *VdDfs* involved in vascular discoloration during infection of cotton by *Verticillium dahliae*.

Fig. S6. Expression analysis of *VdDf1* - *VdDf7*, the seven G-LSR2 genes during infection of cotton by *Verticillium dahliae*.

Fig. S7. PCR verification of the **transfer of two genes D*f5-6* from the wild-type *Verticillium dahliae* strain Vd991 to strains VdLs.17 and VDG78, and mutant background *ΔDfs-1*, respectively.**

Fig. S8. Defoliation phenotypes on okra caused by different *Verticillium dahliae* strains.

Fig. S9. The collaborative role of genes *Df5* and *Df6* in conferring the defoliation phenotype in *Verticillium dahliae* strain VdLs.17.

Fig. S10. Comparison of the sequence divergence of *VdDfs* between *Verticillium dahliae* Vd991 and homologous genes from six different *Fusarium oxysporum* f. sp. *vasinfectum* strains.

Fig. S11. Expression analysis of the *VdDf5* and *VdDf6* homologs, $Df5^{FO}$ and $Df6^{FO}$, during infection of cotton by the ectopic transformant.

Fig. S12. Quantification of NAE 14:0 and NAE 16:0 extracted from different strains by UHPLC-MS/MS.

Fig. S13. Relative expression level analysis of the cotton *GhFAAH* genes in response to D strain Vd991.

Fig. S14. Nucleotide alignment of the published defoliating marker sequence to the genomic region G-LSR2 of *Verticillium dahliae* Vd991.

Fig. S15. Expression analysis of the *VdDf5* and *VdDf6* homologs genes ($Df5^{FO}$ and $Df6^{FO}$) during infection of cotton by *F. oxysporum* f. sp. *vasinfectum.*

Table S1. Information on isolates for which re-sequenced genomes were obtained for this study.

Table S2. Primers used in this study.

Table S3. The coverage breadth and depth of resequenced isolates mapped to encoding genes in G-LSR2.

Table S4. Information of lineage-specific genes in Vd991.

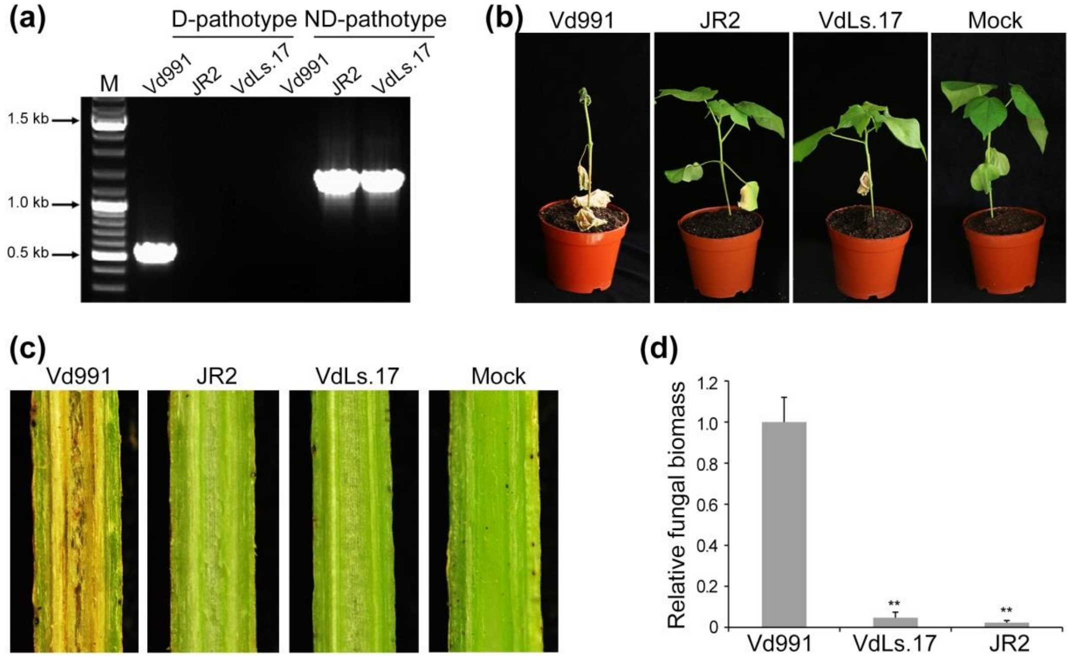

Fig. S1. Cotton defoliation and non-defoliation phenotypes of *Verticillium dahliae* strains Vd991, JR2 and VdLs.17. (**a**) Defoliation (D) and non-defoliation (ND) PCR-based genotypes amplified from genomic DNA of Vd991, JR2 and VdLs.17. M, 2,000 bp DNA ladder. (**b**) Representative defoliating and non-defoliating phenotypes of *V. dahliae* Vd991, JR2 and VdLs.17 on cotton. Four-week-old cotton plants (*G. hirsutum* cv. Junmian No.1) were inoculated with a 1×10^7 conidia/mL suspension using a standard root-dip method, and maintained in a greenhouse at the 25 °C under a 14 h light/10 h dark cycle. Three replicates of 12 seedlings each were included for each treatment. Plants treated with sterile water were used as controls (Mock). Disease symptoms were photographed four weeks after inoculation. (**c**) Vascular discoloration in cotton after inoculation with Vd991, JR2 and VdLs.17. Vertical sections of cotton shoots were photographed 28 days after inoculation. (**d**) Fungal biomass development of the Vd991, JR2 and VdLs.17 strains on cotton as quantified by qPCR. Translation elongation factor 1-α (*EF-1α*) was used as a reference gene to quantify fungal colonization, and the cotton *18S* gene served as an endogenous plant control. Error bars represent standard errors of the mean. **Indicates statistical significance at $P < 0.01$, according to unpaired Student's *t*-tests.

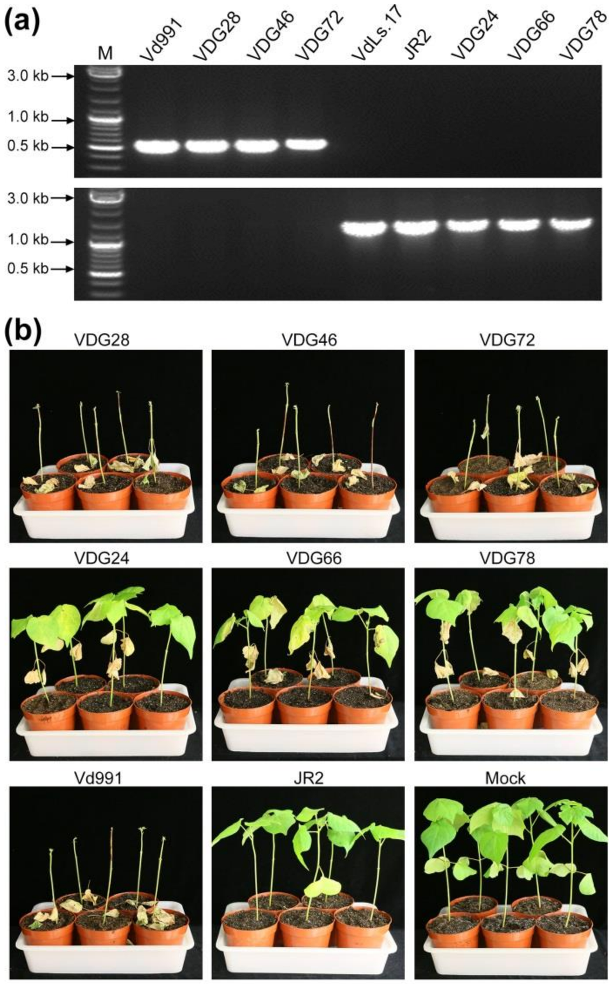

Fig. S2. Defoliation/non-defoliating phenotypes and PCR genotypes of different *Verticillium dahliae* isolates. (**a**) PCR analysis of isolates VDG28, VDG46, VDG72, VDG24, VDG66, VDG78, Vd991, JR2 and VdLs.17 using defoliating and non-defoliating primer pairs. Vd991, VDG28, VDG46 and VDG72 genomic DNA contained the defoliating marker, while JR2, VdLs.17, VDG24, VDG66 and VDG78 contained the non-defoliating markers. M=5,000 bp DNA ladder used as a size marker. (**b**) Defoliating/non-defoliating phenotypes of *V. dahliae* strains VDG28, VDG46, VDG72, VDG24, VDG66, VDG78, Vd991 and JR2 on cotton four weeks after root-dip inoculation with a 1×10^7 conidia/mL suspension. Concordant with PCR results, typical defoliation symptoms were apparent after inoculation with Vd991, VDG28, VDG46 and VDG72, typical non-defoliating symptoms were apparent after inoculation with JR2, VdLs.17, VDG24, VDG66 and VDG78. Mock is control treated with sterile water.

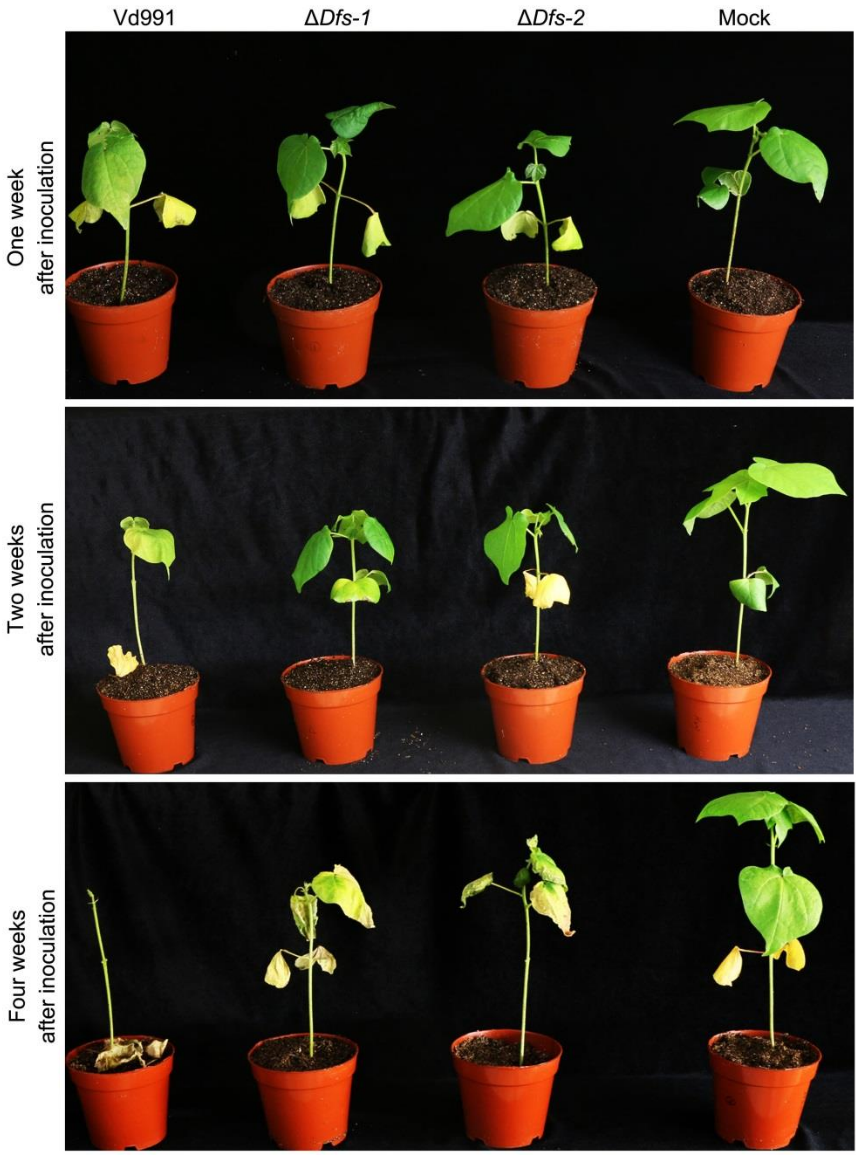

Fig. S3. Cotton defoliation phenotype dynamics from one to four weeks after inoculation with genomic region G-LSR2 deletion mutant *ΔDfs.* Verticillium wilt symptoms observed one, two, and four weeks after root-dip inoculation with conidial suspensions (1×10^7 conidia/mL) from wild-type Vd991 or the Δ*Dfs* strains (constructed by homologous recombination as described in (Chen JY, Liu C, Gui YJ, Si KW, Zhang DD, Wang J, Short DPG, Huang JQ, Li NY, Liang Y, Zhang WQ, Yang L, Ma XF, Li TG, Zhou L, Wang BL, Bao YM, Subbarao KV, Zhang G. Comparative genomics reveals cotton-specific virulence factors in flexible genomic regions in Verticillium dahliae and evidence of horizontal gene transfer from Fusarium. New Phytol. 2018, 217(2):756-770.). Two weeks after inoculation, plants inoculated with Vd991

display early stages of defoliation whereas plants inoculated with Δ*Dfs* display less severe symptoms. Four weeks after inoculation, all leaves of cotton plants inoculated with Vd991 are defoliated whereas cotton plants inoculated with Δ*Dfs* mutants display chlorosis and defoliation of cotyledons only.

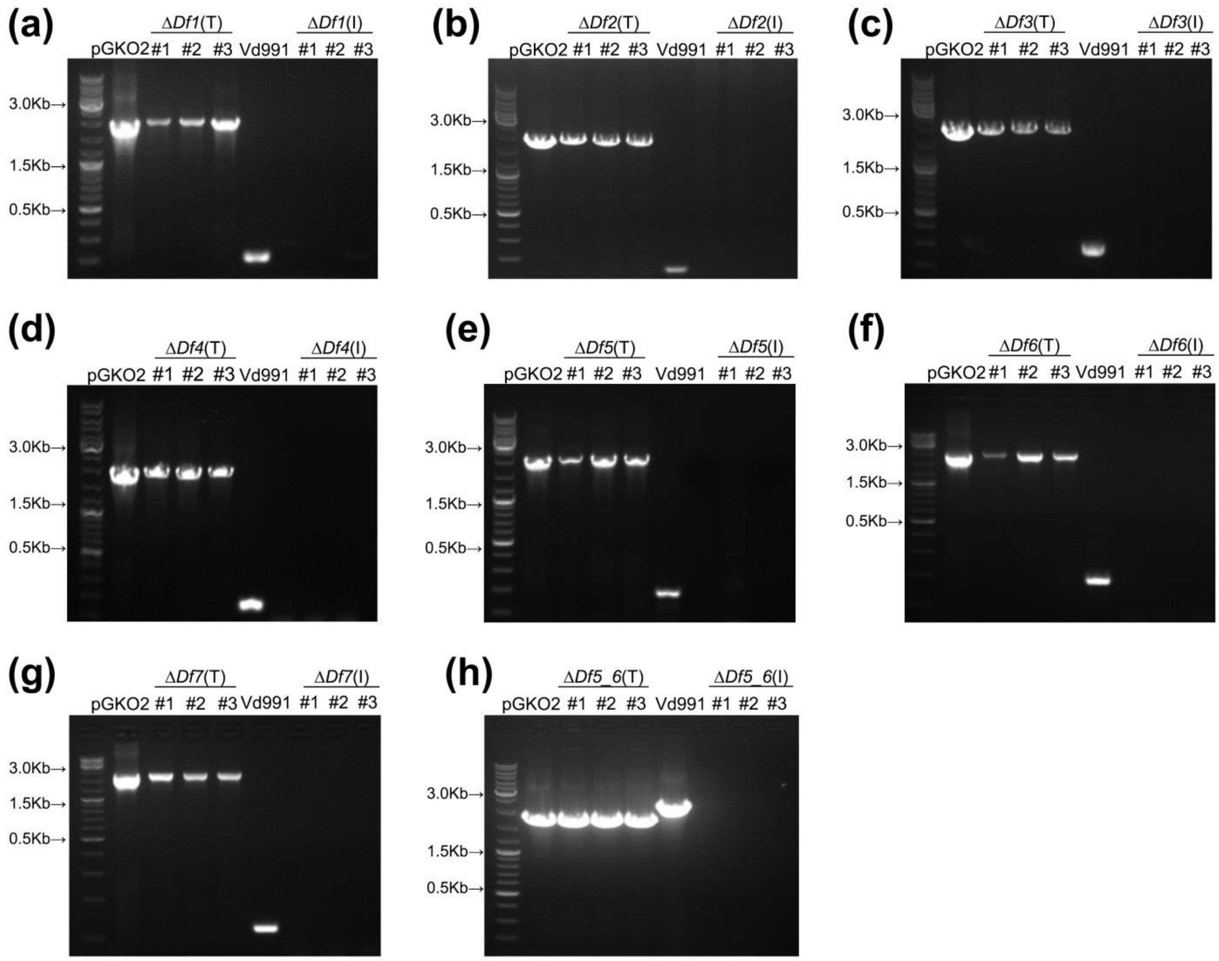

Fig. S4. Screening of gene deletion mutants. (**a-g**) Identification of lineage-specific genomic region single gene deletion mutants, *ΔDf1*-*ΔDf7*. (**h**) Identification of double gene (D*f5*- D*f6*) deletion mutant, *ΔDf5_6*. Three transformants (#1, #2, #3) of each of the deletion mutant strains are shown. *ΔDf1*(T)-*ΔDf7*(T) and *ΔDf5_6*(T): PCR amplification of the positive selection marker hygromycin phosphotransferase; *ΔDf1*-*ΔDf 7*(I) and *ΔDf5_6*(I): PCR amplification of markers specific to the internal gene sequence of *Df1*-*Df7* and *Df5_6*, respectively; Vector pGKO2 and wild-type strain Vd991 were used as the positive controls for hygromycin phosphotransferase and internal gene sequence markers, respectively. M=5,000 bp DNA ladder used as a size marker.

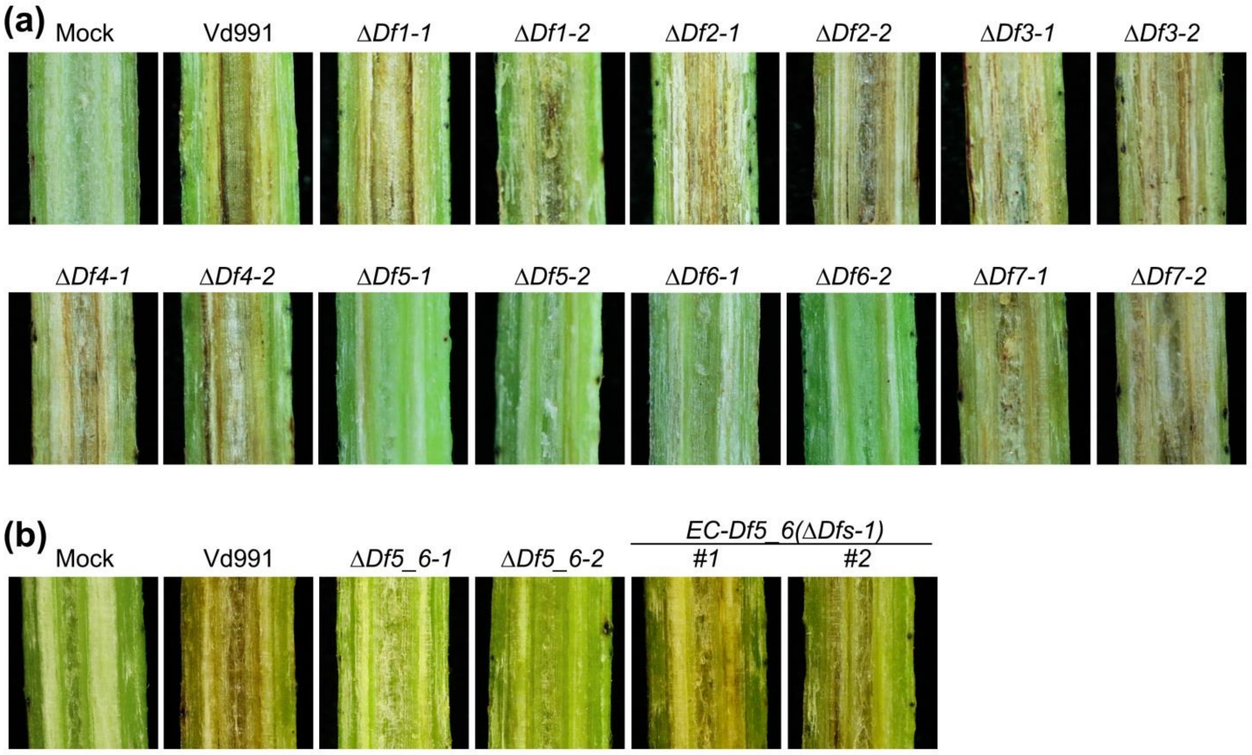

Fig. S5. Identification of functional genes encoded by *VdDfs* involved in vascular discoloration during infection of cotton by *Verticillium dahliae*. (**a**) Vascular discoloration of cotton following inoculations with the targeted gene deletion mutants (*ΔDf1* to *ΔDf7*). Two independent transformants were generated for each gene. (**b**) Vascular discoloration function of *VdDf5* and *VdDf6* double gene deletion mutants. Two independent transformants within which *Df5* and *Df6* were co-deleted (*ΔDf5_6-1* and *ΔDf5_6-2*), and the corresponding ectopic transformants, in which *VdDf5* and *VdDf6* were reintroduced together into *ΔDfs*-1 mutant EC-Df5_6(*ΔDfs-1*). These are shown alongside plants inoculated with the wild-type strain Vd991. Plants treated with sterile water were used as a negative control (Mock). Vascular discoloration was photographed four weeks after inoculation.

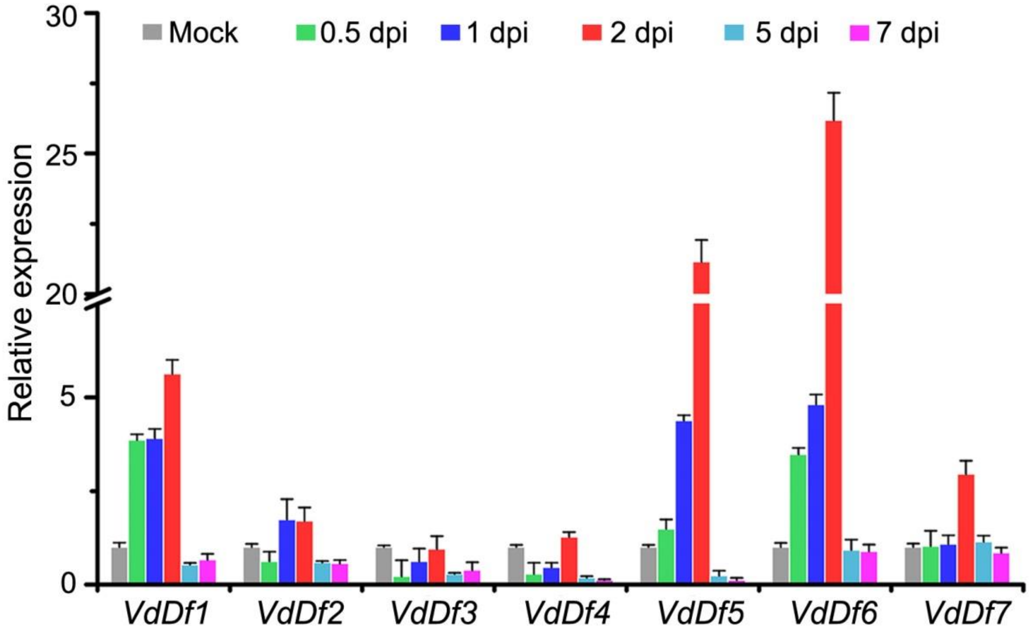

Fig. S6. Expression analysis of the seven G-LSR2 genes, *VdDf1* - *VdDf7*, during infection of cotton by *Verticillium dahliae*. Four-week-old cotton plants (*G. hirsutum* cv. Junmian No.1) were root-dip inoculated with *V. dahliae* Vd991 and harvested at regular intervals from 0.5 to 7 days post inoculation (dpi). After RNA isolation and cDNA synthesis, quantitative reverse transcription polymerase chain reaction was performed to determine the relative expression levels of the seven genes *VdDf1* to *VdDf7* using *V. dahliae* translation elongation factor 1-alpha (*EF-1α)* as a reference, and compared with expression of genes during growth on potato dextrose agar. Error bars represent standard errors.

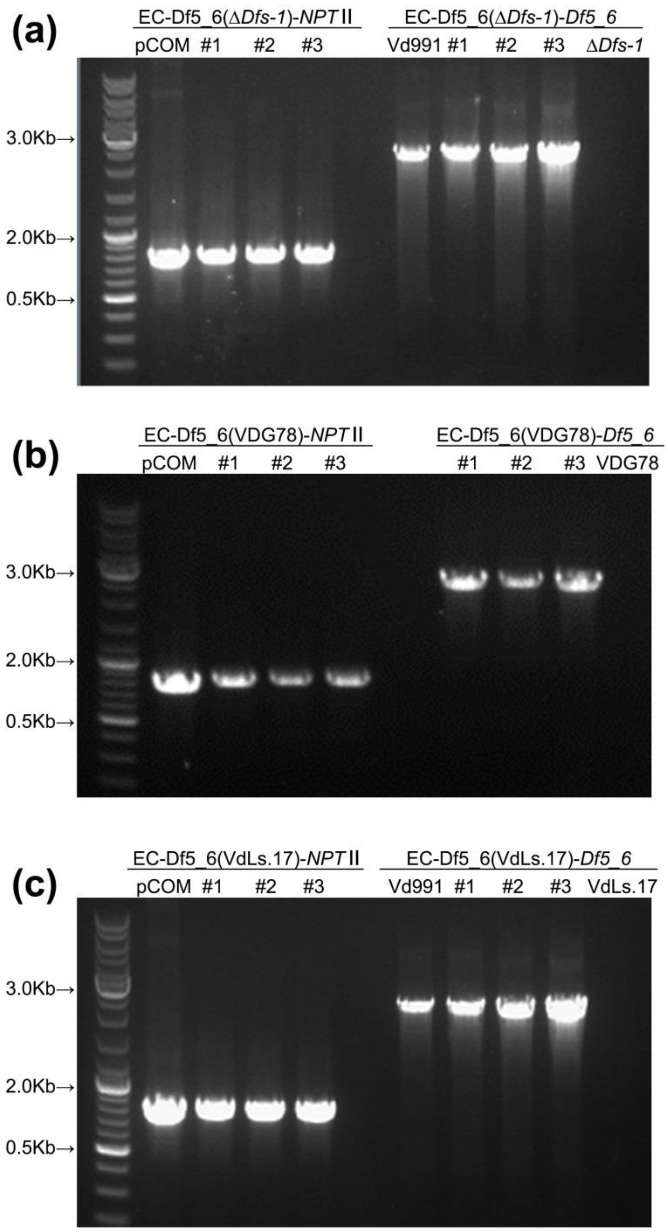

Fig. S7. PCR verification of the **transfer of two genes D*f5-6* from the wild-type *Verticillium dahliae* strain Vd991 to strains VdLs.17 and VDG78, and mutant background *ΔDfs-1*, respectively.** (**a, b, c**) Identification of three positive transformants (#1, 2#, #3) of a double gene transfer (*VdDf5-VdDf6*) to the G-LSR2 deletion mutant *ΔDfs*-1, and strains VDG78 and VdLs.17, respectively. *NPTII* = neomycin phosphotransferase II gene. Genomic DNA from VdLs.17, VDG78 and *ΔDfs*-1 were used as the negative controls for internal gene sequence-amplification of *VdDf5-VdDf6*.

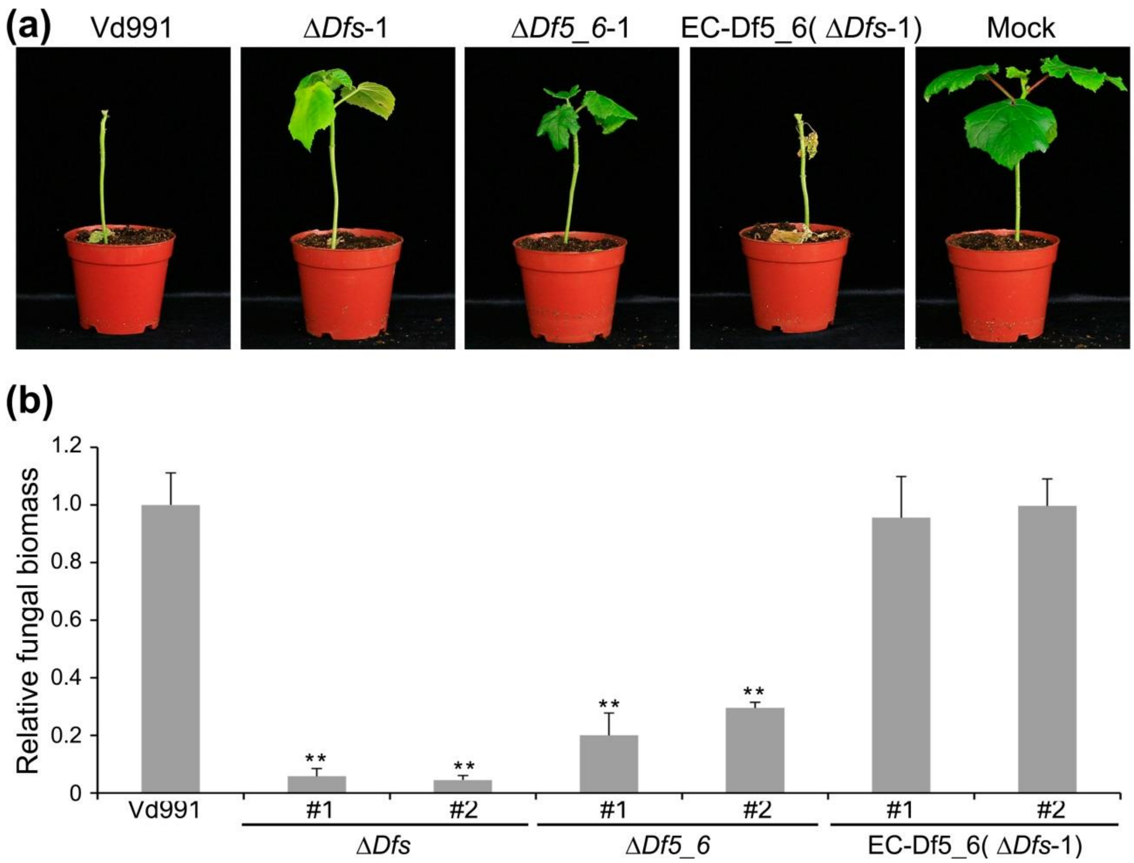

Fig. S8. Defoliation phenotypes on okra caused by different *Verticillium dahliae* strains. (**a**) The defoliating phenotype strains Vd991, *ΔDfs*-1, *ΔDf5_6*-1, EC-Df5_6 (*ΔDfs*-1) on okra. Four-week-old plants were inoculated using a standard root-dip method with 1×10^7 conidia/mL suspensions and photographed three weeks later. (**b**) Quantification of fungal biomass of different *V. dahliae* strains in okra by qPCR. Error bars represent standard errors of the mean. ** represents statistical significance at $P < 0.01$, according to unpaired Student's *t*-tests.

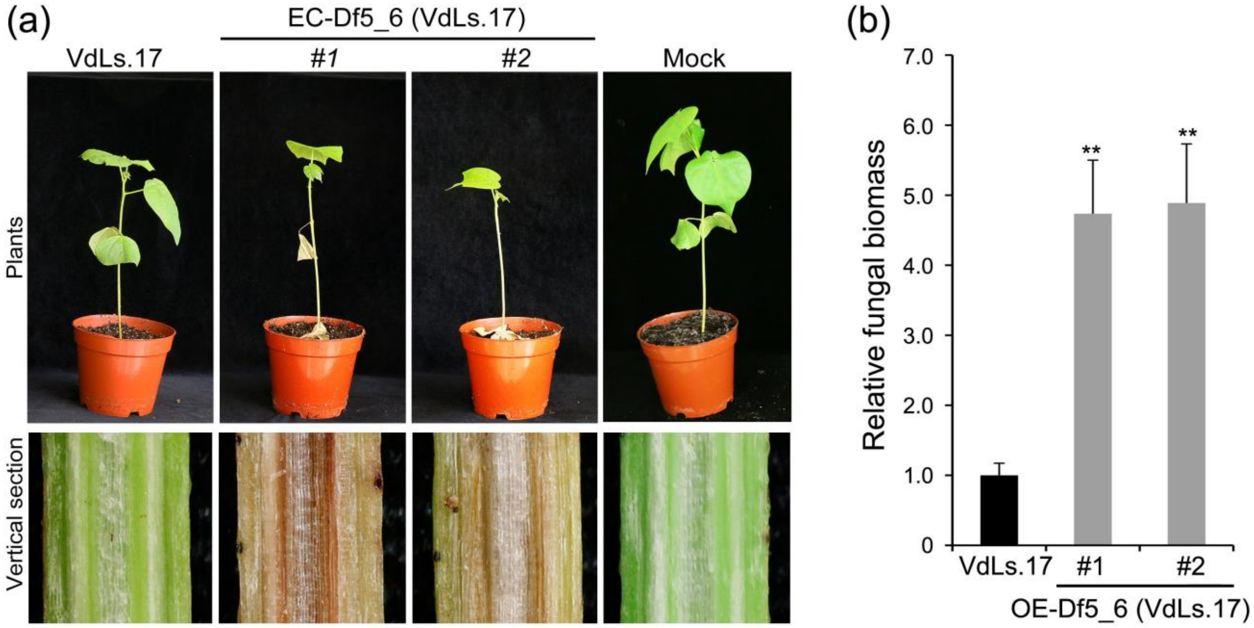

Fig. S9. The collaborative role of genes *Df5* and *Df6* in conferring the defoliation phenotype in *Verticillium dahliae* VdLs.17. (**a**) Virulence and defoliation phenotypes of independent mutants generated by transferring *VdDf5* and *VdDf6* together into *V. dahliae* VdLs.17, originally isolated from lettuce. Four-week-old cotton plants (*G. hirsutum* cv. Junmian No.1) were root-dip inoculated with a 1×10^7 conidia/mL suspension of the ectopic transformants of VdLs.17 that received *VdDf5* and *VdDf6* together. Whole plants and shoot vertical sections were photographed four weeks after inoculation. (**b**) Fungal biomass in cotton plants after inoculation with the ectopic transformants was quantified using qPCR four weeks after inoculation. Error bars represent standard errors, ** Indicates statistical significance at $P < 0.01$, according to unpaired Student's *t*-tests.

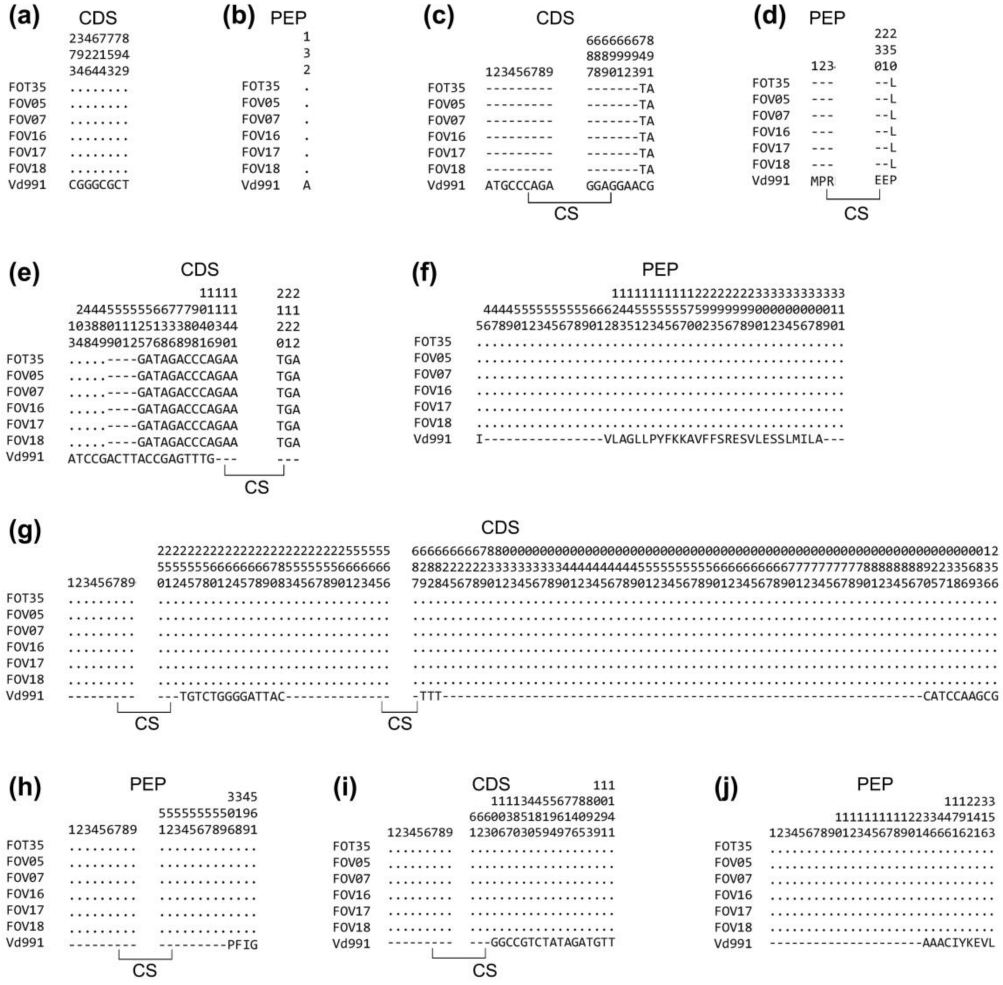

Fig. S10. Comparison of the sequence divergence of *VdDfs* between *Verticillium dahliae* Vd991 and homologous genes from six different *F. oxysporum* f. sp. *vasinfectum* strains. Partial sequence polymorphism comparisons of *VdDf1* (**a and b**), *VdDf2* (**c and d**), *VdDf3* (**e and f**), *VdDf4* (**g and h**), and *VdDf7* (**i and j**) aligned with corresponding homologous genes from six *F. oxysporum* f. sp. *vasinfectum* strains. Five *Fusarium oxysporum* f. sp. *vasinfectum* isolates FOV05, FOV07, FOV16, FOV17 and FOV18 were obtained from cotton. Strain FOT35 is the sequenced strain, *Fusarium oxysporum* f. sp. *vasinfectum* NRRL 25433. ClustalX 1.83 was used for multiple sequence alignments. Only residues that deviated from the reference sequences are shown in the alignment; deletions are indicated by dashes (-); CS = continuous sequences and were omitted. Positions of polymorphisms are written vertically, e.g. the first polymorphism occurs at position 273 of the coding sequence.

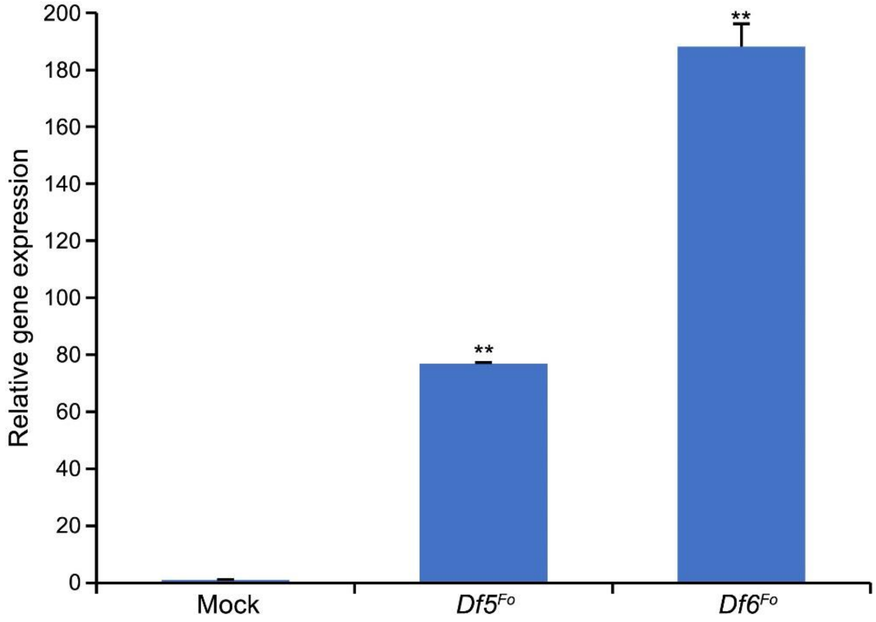

Fig. S11. Expression analysis of the *VdDf5* and *VdDf6* homologs genes, *Df5^{FO}* and *Df6^{FO}*, during infection of cotton by ectopic transformant. Four-week-old cotton plants (*G. hirsutum* cv. Junmian No.1) were root-dip inoculated with ectopic transformant (EC-Df5_6^{FO}, ectopic expression of the *Df5^{FO}* and *Df6^{FO}* from *F. oxysporum* f. sp. *Vasinfectum* in ND strain VDG78) and harvested at five days post inoculation (dpi). After RNA isolation and cDNA synthesis, quantitative reverse transcription polymerase chain reaction was performed to determine the relative expression levels of the *Df5^{FO}* and *Df6^{FO}* using *V. dahliae* translation elongation factor 1-alpha (*EF-1α)* as a reference, and compared with expression of genes during growth on potato dextrose agar. Error bars represent standard errors, ** represents statistical significance $P < 0.01$, according to unpaired Student's *t*-tests.

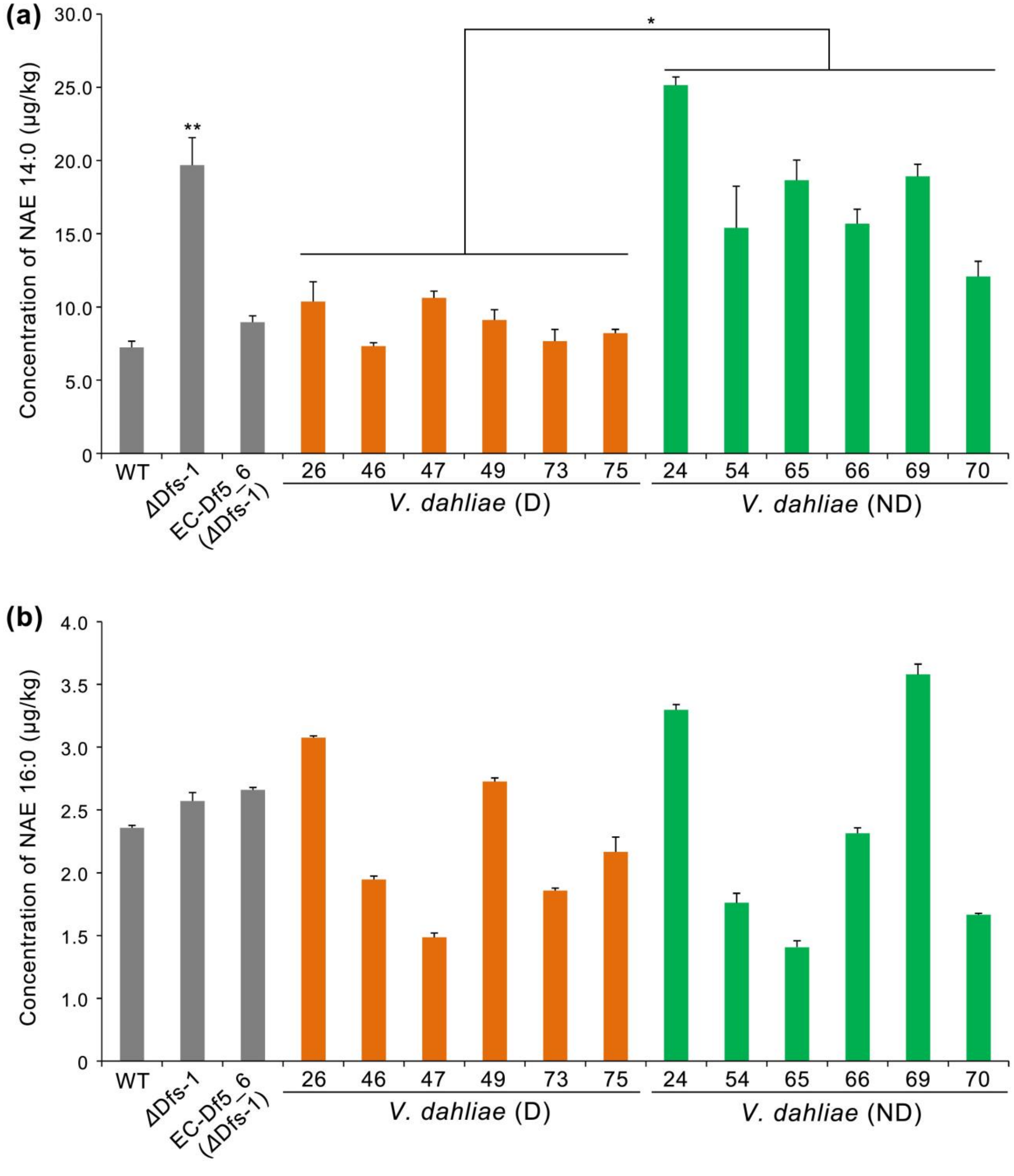

Fig. S12. Quantification of NAE 14:0 and NAE 16:0 extracted from different strains by UHPLC-MS/MS. The strains including wild-type strain Vd991, *VdDfs* deletion strains (Δ*Dfs-1*), complementary transformant of VdDf5 and VdDf6 onΔ*Dfs-1* (EC-Df5_6 (Δ*Dfs-1*)), six D strains and ND strains. Error bars represent standard errors, * and ** represent statistical significance at $P < 0.05$ and $P < 0.01$, respectively, according to an unpaired Student's *t*-tests.

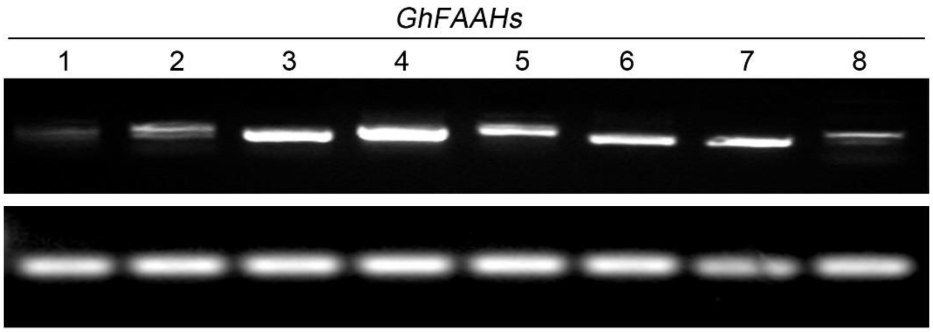

Fig. S13. Relative expression level analysis of the cotton *GhFAAH* genes in response to D strain Vd991. Four-week-old cotton plants (*G. hirsutum* cv. Junmian No.1) were root-dip inoculated with D strain Vd991 and roots were harvested at five days post inoculation (dpi). After RNA isolation and cDNA synthesis, semi-reverse transcription polymerase chain reaction was performed to determine the relative expression levels of the *GhFAAHs* by using the same cDNA concentration and PCR cycle number, cotton *18S* was set as a reference.

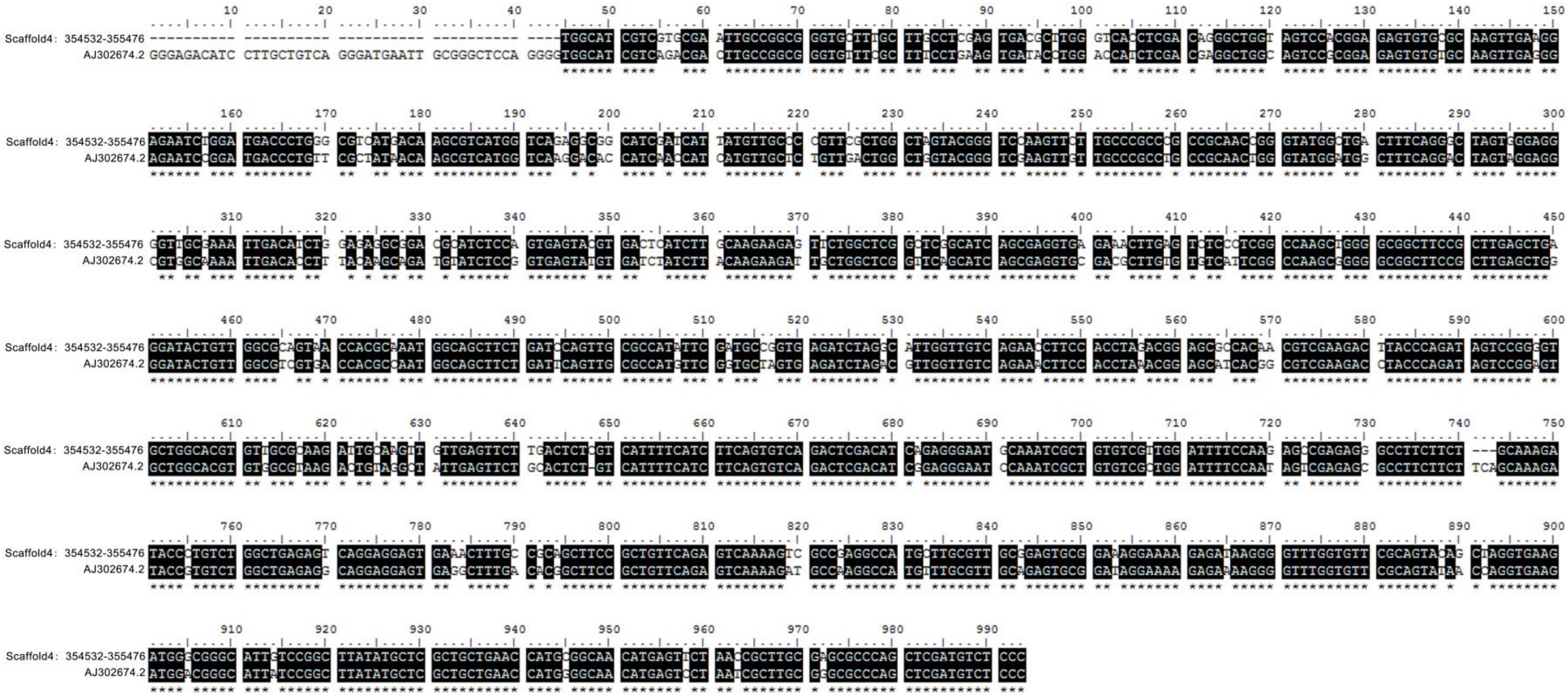

Fig. S14. Nucleotide alignment of the published defoliating marker sequence to the genomic region G-LSR2 of *Verticillium dahliae* Vd991. The defoliating PCR marker sequence AJ302674.2 (992 bp) from *V. dahliae* isolated form cotton reported by Mercado-Blanco et al (Mercado-Blanco J, Rodriguez-Jurado D, Perez-Artes E and Jimenez-Diaz RM. Detection of the defoliating pathotype of *Verticillium dahliae* in infected olive plants by nested PCR. Eur. J. Plant Pathol. 2002, 108:1-13.). shared 85% identity with the sequence of scaffold 4: 354531-355476 (946 bp) of the Vd991 genome, which was located within the G-LSR2 regions (Scaffold4:246496-355314). The conserved nucleotides with black colour background were labeled in asterisk (*).

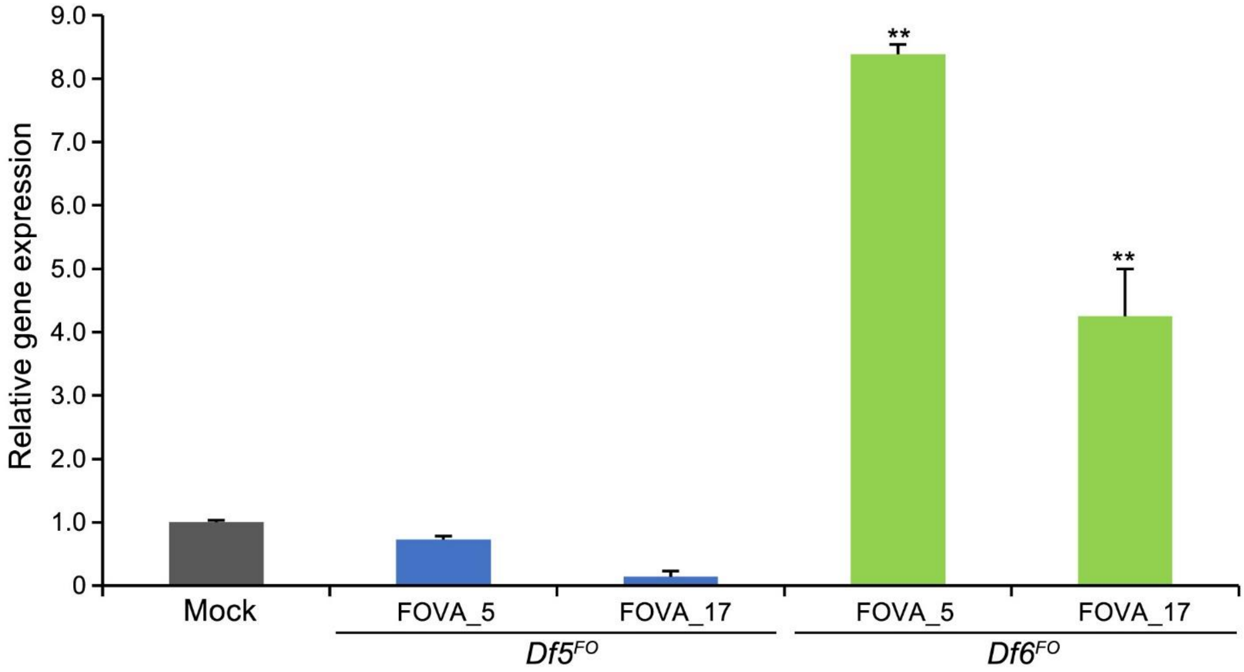

Fig. S15. Expression analysis of the *VdDf5* and *VdDf6* homologs genes (*Df5*^{FO} and *Df6*^{FO}) during infection of cotton by *F. oxysporum* f. sp. *vasinfectum*. Four-week-old cotton plants (*G. hirsutum* cv. Junmian No.1) were root-dip inoculated with two *F. oxysporum* f. sp. *vasinfectum* strains (FOVA_5 and FOVA_17) and roots were harvested at five days post inoculation. After RNA isolation and cDNA synthesis, quantitative reverse transcription polymerase chain reaction was performed to determine the relative expression levels of the *Df5*^{FO} and *Df6*^{FO} using *F. oxysporum* *β-tubulin* (GeneBank ID: KT323834.1) as a reference, and compared with expression of genes during growth on potato dextrose agar. Error bars represent standard errors, ** represents statistical significance $P < 0.01$, according to unpaired Student's *t*-tests.

Table S1. Information on isolates for which genome re-sequenced were obtained for this study

Isolate name	Host of origin	Geographical (Province)	PCR detection
VDG3	*Gossypium hirsutum*	Hubei	D
VDG4	*Gossypium hirsutum*	Hubei	D
VDG5	*Gossypium hirsutum*	Hubei	D
VDG6	*Gossypium hirsutum*	Hubei	D
VDG7	*Gossypium hirsutum*	Hubei	D
VDG8	*Gossypium hirsutum*	Hubei	D
VDG9	*Gossypium hirsutum*	Hubei	D
VDG10	*Gossypium hirsutum*	Hubei	D
VDG11	*Gossypium hirsutum*	Hubei	D
VDG12	*Gossypium hirsutum*	Hubei	D
VDG13	*Gossypium hirsutum*	Hubei	D
VDG14	*Gossypium hirsutum*	Hubei	D
VDG15	*Gossypium hirsutum*	Hubei	D
VDG16	*Gossypium hirsutum*	Hubei	D
VDG17	*Gossypium hirsutum*	Shandong	D
VDG18	*Gossypium hirsutum*	Henan	D
VDG19	*Gossypium hirsutum*	Hebei	D
VDG20	*Gossypium hirsutum*	Hebei	D
VDG21	*Gossypium hirsutum*	Hebei	D
VDG22	*Gossypium hirsutum*	Hebei	D
VDG23	*Gossypium hirsutum*	Hebei	D
VDG25	*Gossypium hirsutum*	Shanxi	D
VDG26	*Gossypium hirsutum*	Henan	D
VDG27	*Gossypium hirsutum*	Henan	D
VDG28	*Gossypium hirsutum*	Shanxi	D
VDG29	*Gossypium hirsutum*	Shanxi	D
VDG31	*Gossypium hirsutum*	Hubei	D
VDG32	*Gossypium hirsutum*	Hubei	D
VDG33	*Gossypium hirsutum*	Hubei	D
VDG34	*Gossypium hirsutum*	Anhui	D
VDG35	*Gossypium hirsutum*	Anhui	D
VDG36	*Gossypium hirsutum*	Anhui	D
VDG37	*Gossypium hirsutum*	Jiangxi	D
VDG38	*Gossypium hirsutum*	Jiangxi	D
VDG39	*Gossypium hirsutum*	Jiangxi	D
VDG40	*Gossypium hirsutum*	Hubei	D
VDG41	*Gossypium hirsutum*	Jiangxi	D
VDG42	*Gossypium hirsutum*	Jiangxi	D
VDG43	*Gossypium hirsutum*	Hubei	D
VDG44	*Gossypium hirsutum*	Hubei	D
VDG45	*Gossypium hirsutum*	Hunan	D
VDG46	*Gossypium hirsutum*	Hunan	D
VDG47	*Gossypium hirsutum*	Hunan	D
VDG48	*Gossypium hirsutum*	Hubei	D
VDG49	*Gossypium hirsutum*	Hunan	D
VDG50	*Gossypium hirsutum*	Hubei	D
VDG51	*Gossypium hirsutum*	Hubei	D
VDG52	*Gossypium hirsutum*	Hunan	D
VDG53	*Gossypium hirsutum*	Hubei	D
VDG57	*Gossypium hirsutum*	Xinjiang	D

Isolate name	Host of origin	Geographical (Province)	PCR detection
VDG60	*Gossypium hirsutum*	Xinjiang	D
VDG61	*Gossypium hirsutum*	Xinjiang	D
VDG63	*Gossypium hirsutum*	Xinjiang	D
VDG64	*Gossypium hirsutum*	Xinjiang	D
VDG67	*Gossypium hirsutum*	Xinjiang	D
VDG68	*Gossypium hirsutum*	Xinjiang	D
VDG73	*Gossypium hirsutum*	Xinjiang	D
VDG75	*Gossypium hirsutum*	Xinjiang	D
VDG76	*Gossypium hirsutum*	Xinjiang	D
VDG2	*Gossypium hirsutum*	Shandong	ND
VDG24	*Gossypium hirsutum*	Hebei	ND
VDG30	*Gossypium hirsutum*	Shanxi	ND
VDG54	*Gossypium hirsutum*	Xinjiang	ND
VDG56	*Gossypium hirsutum*	Xinjiang	ND
VDG58	*Gossypium hirsutum*	Xinjiang	ND
VDG59	*Gossypium hirsutum*	Xinjiang	ND
VDG62	*Gossypium hirsutum*	Xinjiang	ND
VDG65	*Gossypium hirsutum*	Xinjiang	ND
VDG66	*Gossypium hirsutum*	Xinjiang	ND
VDG69	*Gossypium hirsutum*	Xinjiang	ND
VDG70	*Gossypium hirsutum*	Xinjiang	ND
VDG71	*Gossypium hirsutum*	Xinjiang	ND
VDG74	*Gossypium hirsutum*	Xinjiang	ND
VDG77	*Gossypium hirsutum*	Xinjiang	ND
VDG78	*Gossypium hirsutum*	Xinjiang	ND

Note: the defoliating (D) and non-defoliating (ND) were identified using PCR methods (Pérez-Artéz E, Garcia-Pedrajas MD, Bejarano-Alcazar J, Jiménez-Díaz RM. 2000. Differentiation of cotton defoliating and non defoliating pathotypes of Verticillium dahliae by RAPD and specific analysis. European Journal of Plant Pathology 106, 507–517.).

Table S2 Primers used in this study

Primer name		Primer sequence (5′-3′)	Purpose
Defoliating and non-defoliating strain detectoin			
D-F		CATGTTGCTCTGTTGACTGG	primers used for Defoliating Strains from Cotton of *Verticillium dahliae* detecting
D-R		GACACGGTATCTTTGCTGAA	primers used for Defoliating Strains from Cotton of *Verticillium dahliae* detecting
ND-F		ATCAGGGGATACTGGTACGAGA	primers used for Nondefoliating Strains from Cotton of *Verticillium dahliae* detecting
ND-R		GAGTATTGCCGATAAGAACATG	primers used for Nondefoliating Strains from Cotton of *Verticillium dahliae* detecting
Gene expression analysis			
qPCR-VdDf1-F		CCTCCAGACCGAAACCACTAC	RT-qPCR for validation of *VdDf1* expression patterns
qPCR-VdDf1-R		TCACATCCCCATCAATCAACT	RT-qPCR for validation of *VdDf1* expression patterns
qPCR-VdDf2-F		CAGCCAACAGTACTCAGTCCG	RT-qPCR for validation of *VdDf2* expression patterns
qPCR-VdDf2-R		AGTGCCGTTAAGAGCATCCAT	RT-qPCR for validation of *VdDf2* expression patterns
qPCR-VdDf3-F		TGCGGCTGGCTTGTCAGGGGT	RT-qPCR for validation of VdDf3 expression patterns
qPCR-VdDf3-R		CGTCTCACCGAAAATCCGA	RT-qPCR for validation of *VdDf3* expression patterns
qPCR-VdDf4-F		CTCCGTTTCATTCATTTTCG	RT-qPCR for validation of *VdDf4* expression patterns
qPCR-VdDf4-R		CAAGTCATCAGGGTCCAGTA	RT-qPCR for validation of *VdDf4* expression patterns
qPCR-VdDf5-F		TGCCCGTCTTTCCTCGTATG	RT-qPCR for validation of *VdDf5* expression patterns
qPCR-VdDf5-R		GGTTGGGGTTCCCGTTTCTT	RT-qPCR for validation of *VdDf5* expression patterns
qPCR-VdDf6-F		GGAGTACACGAAAGCCGAAG	RT-qPCR for validation of *VdDf6* expression patterns
qPCR-VdDf6-R		TGCAAATTGAATGGCAAAGG	RT-qPCR for validation of *VdDf6* expression patterns
qPCR-VdDf7-F		TACCCGTACCAAATCGAACAA	RT-qPCR for validation of *VdDf7* expression patterns
qPCR-VdDf7-R		GGTGAAAGGAGAGCAGCAGTC	RT-qPCR for validation of *VdDf7* expression patterns
Gene deletion			
VdDf1	P1	GCAGAAGCAGAAGCAGTTGTTG	Upstream amplication
	P3	GCCCAAAAATGCTCCTTCAATAGAGGGCTGTTAGTTGTCGCA	Upstream amplication
	P4	CCCTGGGTTCGCAAAGATAACACTCATTACGAATCACAACGG	downstream amplication
	P6	CAACGACAAACACTCTCCTCTC	downstream amplication
	P2	GGGGACAAGTTTGTACAAAAAAGCAGGCTAATTCGAGGAATGGGA[illegible]	nested PCR reaction for final amplification
	P5	GGGGACCACTTTGTACAAGAAAGCTGGGTCCGACGGTGTATGGAT[illegible]	nested PCR reaction for final amplification
	T-F	CCTCCAGACCGAAACCACTAC	mutant detection, amplication of gene *VdDf1*
	T-R	TCACATCCCCATCAATCAACT	mutant detection, amplication of gene *VdDf1*
VdDf2	P1	ATGTGACAGGGGTAATGTGGGG	Upstream amplication
	P3	GCCCAAAAATGCTCCTTCAATGGGGTGGGTGATGACTTGGAG	Upstream amplication
	P4	CCCTGGGTTCGCAAAGATAATTATATCCCATGCGATTCCA	downstream amplication
	P6	CAACTATTCCAGAGCCAGCT	downstream amplication
	P2	GGGGACAAGTTTGTACAAAAAAGCAGGCTATGCTACAGTGGAGGG[illegible]	nested PCR reaction for final amplification

Primer name		Primer sequence (5′-3′)	Purpose
	P5	GGGGACCACTTTGTACAAGAAAGCTGGGTCAGCTTGGCTTTCATAA	nested PCR reaction for final amplification
	T-F	CAGCCAACAGTACTCAGTCCG	mutant detection, amplication of gene *VdDf2*
	T-R	AGTGCCGTTAAGAGCATCCAT	mutant detection, amplication of gene *VdDf2*
VdDf3	P1	GGTAGGCAAATAAAGGACTAAA	Upstream amplication
	P3	GCCCAAAAATGCTCCTTCAACAAAGAAAGATGGGATACAAGT	Upstream amplication
	P4	CCCTGGGTTCGCAAAGATAACTCCCTGGCGTCGGTGCTAATT	downstream amplication
	P6	AGCTGTTGCATCGCATGGTCTT	downstream amplication
	P2	GGGGACAAGTTTGTACAAAAAAGCAGGCTGTATCCTCATCTATGCC	nested PCR reaction for final amplification
	P5	GGGGACCACTTTGTACAAGAAAGCTGGGTTATGTGACCAGTCAGTT	nested PCR reaction for final amplification
	T-F	TGCGGCTGGCTTGTCAGGGGT	mutant detection, amplication of gene *VdDf3*
	T-R	CGTCTCACCGAAAATCCGA	mutant detection, amplication of gene *VdDf3*
VdDf4	P1	CTTGGGTAGGGAGAGGCTTC	Upstream amplication
	P3	GCCCAAAAATGCTCCTTCAATACGTTCTGCGTTTTCGGGT	Upstream amplication
	P4	CCCTGGGTTCGCAAAGATAAAATCGCTGCCGCTCTACTTACT	downstream amplication
	P6	TACCTTTACATGCCGCTCCTTG	downstream amplication
	P2	GGGGACAAGTTTGTACAAAAAAGCAGGCTAAAAACAGTCGATTGTC	nested PCR reaction for final amplification
	P5	GGGGACCACTTTGTACAAGAAAGCTGGGTAGAACATTGAATGAGTA	nested PCR reaction for final amplification
	T1-F	CTCCGTTTCATTCATTTTCG	mutant detection, amplication of gene *VdDf4*
	T1-R	CAAGTCATCAGGGTCCAGTA	mutant detection, amplication of gene *VdDf4*
VdDf5	P1	GGCTGTGGAGATGACAAGACGA	Upstream amplication
	P3	GCCCAAAAATGCTCCTTCAACTCACTGCCGATGCCAAGAATG	Upstream amplication
	P4	CCCTGGGTTCGCAAAGATAAATACCCTTGCGTCTTGCTCA	downstream amplication
	P6	GGTTCCGACTCCTCCTGTGC	downstream amplication
	P2	GGGGACAAGTTTGTACAAAAAAGCAGGCTGGCTTAGGTGTCAGCT GGTTGG	nested PCR reaction for final amplification
	P5	GGGGACCACTTTGTACAAGAAAGCTGGGTCCTGGTAGGCCCTTTT GTGTGT	nested PCR reaction for final amplification
	T-F	TGCCCGTCTTTCCTCGTATG	mutant detection, amplication of gene *VdDf5*
	T-R	GGTTGGGGTTCCCGTTTCTT	mutant detection, amplication of gene *VdDf5*
VdDf6	P1	CAGCCCGACGGGATACGAGAGA	Upstream amplication
	P3	GCCCAAAAATGCTCCTTCAAGGGGAACCAATGACGAATGAAA	Upstream amplication
	P4	CCCTGGGTTCGCAAAGATAATAACCTTGCCTGCTCCACCACC	downstream amplication
	P6	CTCAGCTCCGCATCCTCCCACC	downstream amplication
	P2	GGGGACAAGTTTGTACAAAAAAGCAGGCTGGAACGGGTCTAGGAA GAAACG	nested PCR reaction for final amplification
	P5	GGGGACCACTTTGTACAAGAAAGCTGGGTGAAGGGTGAAAGGAG AGCAGCA	nested PCR reaction for final amplification
	T-F	GGAGTACACGAAAGCCGAAG	mutant detection, amplication of gene *VdDf6*
	T-R	TGCAAATTGAATGGCAAAGG	mutant detection, amplication of gene *VdDf6*
	P1	CCACGGGTGCAAGGTGACGGTA	Upstream amplication

Primer name		Primer sequence (5′-3′)	Purpose
	P3	GCCCAAAAATGCTCCTTCAAGGGAGAGGGGAGGGGAAATAGG	Upstream amplication
	P4	CCCTGGGTTCGCAAAGATAAAAGGGAGAGAAACAAAGGGCTG	downstream amplication
	P6	GGAGTGGTACTAGAGTGAGGCA	downstream amplication
VdDf7	P2	GGGGACAAGTTTGTACAAAAAAGCAGGCTACTATCGAGCGGAAGA CCTGGT	nested PCR reaction for final amplification
	P5	GGGGACCACTTTGTACAAGAAAGCTGGGTGGCCTTATTGGAAGA ACTAAG	nested PCR reaction for final amplification
	T-F	TACCCGTACCAAATCGAACAA	mutant detection, amplication of gene *VdDf7*
	T-R	GGTGAAAGGAGAGCAGCAGTC	mutant detection, amplication of gene *VdDf7*
	P1	GGCTGTGGAGATGACAAGACGA	To amplify upstream DNA fragment of *VdDf5-6*
	P3	GCCCAAAAATGCTCCTTCAACTCACTGCCGATGCCAAGAATG	To amplify upstream DNA fragment of *VdDf5-6*
	P4	CCCTGGGTTCGCAAAGATAATAACCTTGCCTGCTCCACCACC	To amplify downstream DNA fragment of *VdDf5-6*
	P6	CTCAGCTCCGCATCCTCCCACC	To amplify downstream DNA fragment of *VdDf5-6*
VdDf5-6	P2	GGGGACAAGTTTGTACAAAAAAGCAGGCTGGCTTAGGTGTCAGCT GGTTGG	nested PCR reaction for final amplification
	P5	GGGGACCACTTTGTACAAGAAAGCTGGGTCCTGGTAGGCCCTTTT GTGTGT	nested PCR reaction for final amplification
	T-F	GGGTTGGTCTGCTTCCTGTTT	primers used for *VdDf5-6* transgenic lines detecting
	T-R	GCTCGCATGGTGCTATGTGTA	primers used for *VdDf5-6* transgenic lines detecting
Hpt gene	Hpt-F	TTGAAGGAGCATTTTTGGGC	*Hpt* gene amplication and mutant detection
	Hpt-R	TTATCTTTGCGAACCCAGGG	*Hpt* gene amplication and mutant detection
VdDf5-6 complementation			
EC-Df5_6-F		CGCGGATCCCCGAACGGCAAGTTGATGTAAAA	To amplify the DNA fragment for *Df5_6* complementary vector
EC-Df5_6-R		CGCGGATCCGGCCTAGAAGGGTGAAAGGAGAG	To amplify the DNA fragment for *Df5_6* complementary vector
Df5-6 -F		GGGTTGGTCTGCTTCCTGTTT	primers used for *Df5_6* complementary transgenic lines detecting
Df5-6 -R		GCTCGCATGGTGCTATGTGTA	primers used for *Df5_6* complementary transgenic lines detecting
FoDf5-6 complementation			
EC-Df5_6-F		CGCGGATCCCCGAACGGCAAGTTGATGTAAAA	To amplify the DNA fragment for *VdDf5-6* homogous gene from *F. oxysporum* f. sp. *vasinfectum*
EC-Df5_6-R		CGCGGATCCGGCCTAGAAGGGTGAAAGGAGAG	To amplify the DNA fragment for *VdDf5-6* homogous gene from *F. oxysporum* f. sp. *vasinfectum*
Df5-6 -F		CTTTTCTGGTTGCGAGGGTT	primers used for EC-DF5_6 ^{FO} complementary transgenic lines detecting
Df5-6 -R		CTTGCGAATGGGGTGTATGA	primers used for EC-DF5_6 ^{FO} complementary transgenic lines detecting

Primer name	Primer sequence (5′-3′)	Purpose
Housekeeping genes		
EF-1α-F	TGAGTTCGAGGCTGGTATCT	*V.dahliae EF-1α* gene used for fungal biomass detection
EF-1α-R	CACTTGGTGGTGTCCATCTT	*V.dahliae EF-1α* gene used for fungal biomass detection
18S-F	CGGCTACCACATCCAAGGAA	Cotton 1*8S* gene used for fungal biomass detection
18S-R	TGTCACTACCTCCCCGTGTCA	Cotton 1*8S* gene used for fungal biomass detection
Fotu-F	CATCGGAAACTCCACCTCTATC	*F. oxysporum β-tubulin* gene used for gene expression analysis
Fotu-R	TACTCCTCTTCCTCCTCATCAA	*F. oxysporum β-tubulin* gene used for gene expression analysis
Homologous Genes amplication from different Fusarium Oxysporum strains		
FOTG_17883-F	CTAGCCCCGTGGTTCTCTAC	To amplify the homologous gene sequence of FOTG_17883
FOTG_17883-R	TACCTGGGTCCGACATTTTT	To amplify the homologous gene sequence of FOTG_17883
FOTG_17885-F	CTGGTGTGTGTGAACTTTAGGC	To amplify the homologous gene sequence of FOTG_17885
FOTG_17885-R	CACGAATGGAATGATTGGCTAT	To amplify the homologous gene sequence of FOTG_17885
FOTG_17887-F	CCCTTGAACTTGTATCCCATC	To amplify the homologous gene sequence of FOTG_17887
FOTG_17887-R	TCCAACGTTATCCAGCTCTCT	To amplify the homologous gene sequence of FOTG_17887
FOTG_16738-F	GCTGCCCACCCGTTACATACA	To amplify the homologous gene sequence of FOTG_16738
FOTG_16738-R	CAAGCGACAAGTCCACCACAT	To amplify the homologous gene sequence of FOTG_16738
FOTG_16737-F	CTTTTCTGGTTGCGAGGGTT	To amplify the homologous gene sequence of FOTG_16737
FOTG_16737-R	CTTGCGAATGGGGTGTATGA	To amplify the homologous gene sequence of FOTG_16737
FOTG_16736-F	GGCGTCGGAGTATGGTATTGAG	To amplify the homologous gene sequence of FOTG_16736
FOTG_16736-R	GTGGGAAAGGTTGTGGTGGTAG	To amplify the homologous gene sequence of FOTG_16736
FOTG_16735-F	GCGTCATCAGGGGTAGAGGC	To amplify the homologous gene sequence of FOTG_16735
FOTG_16735-R	GGCAAAGAAGGGGCAAAGTG	To amplify the homologous gene sequence of FOTG_16735

Table S3. The coverage breadth and depth of resequenced isolates mapped to encoding genes in G-LSR2

Isolates \ Gene ID		VEDA_05181	VEDA_05182	VEDA_05183	VEDA_05184	VEDA_05185	VEDA_05186	VEDA_05187	VEDA_05188	VEDA_05189	VEDA_05190	VEDA_05191	VEDA_05192	VEDA_05193	VEDA_05194	VEDA_05195	VEDA_05196	VEDA_05197	VEDA_05198	VEDA_05199	VEDA_05200	VEDA_05201	VEDA_05202	VEDA_05203
Coverage breadth (from white to red color represents the gene coverage breadth of mapping reads from 0% to 100%)																								
	VDG66	89.23	0.00	0.00	0.00	0.00	4.11	0.00	0.00	0.00	0.00	0.00	0.00	0.00	0.00	0.00	4.97	0.00	0.00	0.00	0.00	0.00	0.00	0.00
	VDG24	96.11	18.92	17.83	13.61	5.49	12.33	8.99	12.99	0.00	20.69	15.63	5.59	0.00	9.55	10.11	14.92	17.29	0.00	14.16	0.00	23.15	0.00	0.00
	VDG30	91.98	0.00	0.00	0.00	0.00	2.05	0.00	0.00	0.00	0.00	0.00	0.00	8.06	0.00	0.00	4.97	0.00	0.00	0.00	0.00	0.00	0.00	0.00
	VDG2	83.28	0.00	0.00	0.00	0.00	4.11	0.00	0.00	0.00	6.90	15.63	0.00	16.13	0.00	0.00	0.00	0.00	0.00	0.00	0.00	0.00	0.00	0.00
	VDG56	74.23	0.00	0.00	0.00	0.00	0.00	0.00	0.00	0.00	0.00	0.00	0.00	0.00	0.00	0.00	0.00	0.00	0.00	0.00	0.00	0.00	0.00	0.00
	VDG54	86.25	0.00	0.00	6.80	0.00	0.00	0.00	0.00	0.00	13.79	0.00	0.00	0.00	0.00	0.00	0.00	0.00	0.00	0.00	0.00	0.00	0.00	0.00
	VDG58	94.27	0.00	0.00	0.00	0.00	0.00	0.00	0.00	0.00	0.00	0.00	0.00	0.00	0.00	0.00	0.00	0.00	0.00	0.00	0.00	0.00	0.00	0.00
ND	VDG59	91.41	0.00	0.00	0.00	0.00	2.05	0.00	0.00	0.00	0.00	0.00	0.00	0.00	0.00	0.00	0.00	0.00	0.00	0.00	0.00	0.00	0.00	0.00
	VDG78	90.03	0.00	5.94	0.00	0.00	0.00	0.00	0.00	0.00	0.00	0.00	0.00	0.00	0.00	0.00	0.00	0.00	0.00	0.00	0.00	0.00	0.00	0.00
	VDG62	89.69	3.38	0.00	0.00	0.00	0.00	0.00	0.00	0.00	0.00	0.00	0.00	0.00	0.00	0.00	0.00	0.00	0.00	0.00	0.00	0.00	0.00	0.00
	VDG71	87.86	0.00	0.00	0.00	0.00	0.00	0.00	0.00	0.00	0.00	0.00	0.00	0.00	0.00	0.00	0.00	0.00	0.00	0.00	0.00	0.00	0.00	0.00
	VDG77	90.26	0.00	0.00	13.61	0.00	0.00	0.00	0.00	0.00	0.00	0.00	0.00	0.00	0.00	0.00	0.00	0.00	0.00	0.00	0.00	0.00	0.00	0.00
	VDG65	90.38	0.00	0.00	0.00	0.00	0.00	0.00	0.00	0.00	0.00	0.00	0.00	0.00	0.00	0.00	0.00	0.00	0.00	0.00	0.00	0.00	0.00	0.00
	VDG69	91.52	0.00	0.00	0.00	0.00	0.00	0.00	0.00	0.00	0.00	0.00	5.59	0.00	0.00	0.00	0.00	0.00	0.00	0.00	0.00	0.00	0.00	0.00
	VDG70	92.21	0.00	0.00	0.00	0.00	0.00	0.00	0.00	0.00	0.00	0.00	0.00	0.00	0.00	7.91	0.00	0.00	0.00	0.00	0.00	0.00	0.00	0.00
	VDG74	85.68	0.00	0.00	0.00	10.53	0.00	4.03	0.00	0.00	0.00	15.63	0.00	0.00	0.00	0.00	0.00	0.00	0.00	0.00	0.00	0.00	5.23	0.00
	VDG39	99.43	99.81	99.21	86.70	97.28	99.89	95.48	97.84	98.03	99.16	97.40	99.81	98.75	99.89	99.47	99.17	98.66	99.30	98.03	93.75	98.91	99.56	99.59
	VDG73	98.17	97.74	97.82	97.88	98.75	99.22	95.35	91.34	97.05	98.77	96.70	99.32	98.66	98.20	99.12	92.93	99.71	98.80	97.40	93.75	99.46	99.10	97.99
	VDG32	93.93	98.35	99.67	98.03	99.48	99.54	96.11	97.98	97.38	98.08	97.74	97.77	99.82	99.26	98.95	94.31	94.24	98.70	97.25	98.78	99.13	99.01	99.76
	VDG63	97.71	98.83	98.61	97.28	98.69	98.36	95.35	98.99	90.16	98.70	89.58	99.19	98.39	98.94	99.56	92.87	96.25	98.40	98.19	91.71	97.39	99.59	98.76
	VDG75	97.14	97.63	99.70	95.09	97.71	98.56	96.33	97.84	94.43	99.69	97.57	99.19	98.21	90.34	97.54	94.86	94.43	97.80	96.54	97.42	98.91	99.30	96.74
	VDG57	98.17	98.53	99.11	95.77	99.51	97.90	95.62	97.84	98.03	96.86	92.01	99.75	99.46	97.03	99.30	92.71	99.71	97.80	91.66	97.42	98.70	96.49	99.23
	VDG14	98.17	99.06	99.17	95.09	94.60	99.61	95.70	97.55	86.23	99.62	97.74	99.44	98.75	86.09	97.72	91.22	97.60	98.10	95.20	92.93	96.85	98.55	96.68
	VDG49	99.31	97.82	98.84	95.46	99.18	99.38	95.75	96.68	97.05	98.70	94.79	99.01	96.86	99.36	98.15	97.40	97.69	99.30	95.52	78.67	98.91	99.36	98.82
	VDG22	99.54	96.20	99.60	97.28	99.42	98.45	95.84	93.36	97.38	98.24	96.01	97.58	97.85	99.15	99.74	93.92	99.42	96.31	98.35	97.01	98.59	99.65	98.11
	VDG61	98.17	97.82	99.54	91.53	79.22	99.84	95.48	98.85	93.44	97.62	99.13	99.01	98.92	96.71	99.38	94.48	97.50	97.70	97.25	92.66	98.59	97.71	98.70
	VDG27	98.40	98.65	99.93	94.41	98.69	99.73	95.17	89.47	95.41	97.62	99.31	97.77	97.76	97.66	99.56	94.31	97.50	98.10	97.88	93.61	98.26	99.77	98.99
	VDG9	97.37	97.14	98.75	91.61	98.84	98.08	92.30	97.98	93.77	98.85	98.26	98.76	98.66	93.84	98.95	94.70	97.89	96.81	98.74	93.34	92.07	96.72	96.63
	VDG21	98.17	97.82	99.93	98.03	99.97	99.47	96.38	96.10	98.36	99.77	99.83	99.26	99.82	92.99	99.65	99.78	99.81	99.10	96.46	95.79	99.35	99.36	96.74
	VDG52	96.56	98.68	96.40	98.26	96.46	99.02	94.99	98.41	98.03	97.55	99.48	99.13	97.58	99.15	95.43	92.60	97.98	96.51	95.20	91.44	99.02	99.85	96.86
	VDG16	96.56	96.50	98.38	96.75	95.64	98.36	97.72	94.37	70.82	96.17	93.06	98.01	97.67	89.70	96.66	90.61	95.58	95.11	93.63	94.84	98.80	95.24	97.81
	VDG76	96.91	96.20	99.08	92.21	97.65	98.81	94.90	99.28	95.74	96.93	99.48	99.57	99.28	97.98	99.03	94.42	95.77	87.33	94.02	91.44	98.80	98.46	99.23
	VDG8	98.74	96.09	98.75	95.24	99.24	99.29	95.48	96.39	93.44	99.62	99.65	99.57	99.64	89.81	99.21	94.14	96.54	97.01	95.28	86.96	98.80	99.48	96.98
	VDG48	95.88	97.71	98.98	95.62	97.74	99.16	94.54	93.65	92.79	99.23	99.13	94.54	99.37	96.18	97.89	94.03	96.83	94.71	96.70	99.05	99.13	98.05	97.99
	VDG42	96.56	97.86	99.31	95.39	98.38	99.57	95.39	97.26	98.36	99.00	98.44	99.81	98.75	98.94	99.12	99.12	97.60	99.40	98.11	96.88	99.89	98.84	98.99
	VDG37	97.25	96.39	99.70	87.83	98.81	98.06	96.15	99.71	88.52	99.62	98.09	96.52	99.01	92.99	99.12	95.80	93.76	92.02	92.37	92.39	98.59	98.05	95.86
	VDG43	99.08	98.19	99.90	89.12	98.72	99.43	96.55	96.54	92.46	97.85	96.53	96.15	99.01	95.97	99.03	94.36	93.76	95.51	96.14	95.24	98.15	97.62	99.05
	VDG6	97.02	97.89	99.21	96.22	96.22	99.36	96.11	95.96	96.72	98.31	98.26	97.58	97.76	97.77	99.65	98.56	98.66	96.11	95.28	95.79	98.59	98.93	98.58
	VDG11	96.56	95.11	97.92	95.92	95.30	96.83	94.94	97.84	97.38	98.39	98.26	97.45	99.64	88.11	98.15	94.36	97.69	97.90	95.44	88.32	94.78	99.62	97.22
	VDG44	98.97	99.62	99.67	95.31	99.15	99.52	96.20	97.84	98.03	98.47	98.61	99.69	98.39	95.22	98.95	94.86	98.85	96.61	95.75	99.05	99.57	98.69	99.88
	VDG51	98.97	95.71	99.04	98.34	96.25	98.70	94.81	99.42	99.34	99.77	98.61	98.39	95.61	97.77	99.38	91.55	93.56	98.70	97.80	93.48	96.85	96.95	97.22
	VDG64	95.53	96.54	97.76	95.62	99.73	99.66	94.72	98.12	96.72	95.71	96.35	96.96	97.85	96.82	99.03	95.75	96.93	99.60	90.32	94.84	98.59	99.71	98.76
	VDG45	99.08	97.41	99.70	99.09	97.71	99.89	95.48	88.31	97.05	99.16	97.57	98.01	99.37	96.82	99.38	93.54	95.39	94.71	99.61	94.70	98.70	99.25	98.70
	VDG53	97.71	97.44	99.34	93.27	95.54	99.82	96.20	93.65	95.08	98.39	97.57	98.88	99.55	94.37	99.47	95.14	98.37	96.31	90.64	94.97	99.02	99.19	99.64
	VDG25	99.66	98.35	98.84	91.61	99.05	99.70	97.72	96.83	97.38	99.39	97.40	98.57	99.28	99.79	99.03	93.70	97.79	99.00	97.48	94.02	98.04	99.07	98.82
D	VDG7	97.71	98.57	99.37	94.03	93.93	98.58	95.62	97.11	91.48	99.92	94.97	97.58	99.46	95.01	99.21	97.79	97.79	92.22	92.68	92.12	97.50	98.34	95.38
	VDG31	99.54	98.53	99.93	99.32	98.84	99.45	95.70	97.55	97.05	98.24	99.13	99.44	99.82	98.41	99.65	94.86	97.98	98.40	97.40	97.96	99.67	99.45	99.11
	VDG38	99.31	97.63	99.41	98.11	99.91	99.79	94.32	97.55	93.11	99.46	98.44	98.39	99.46	99.89	99.30	99.45	98.17	98.50	98.74	98.23	99.57	99.83	98.46
	VDG20	98.51	97.29	99.64	99.92	97.22	99.95	97.63	98.99	98.03	97.93	98.96	99.32	98.92	98.20	96.75	93.98	97.69	97.60	97.48	94.29	99.57	98.81	95.09
	VDG34	99.31	99.02	98.88	96.30	99.76	99.84	96.38	99.42	90.16	99.16	96.88	99.26	97.31	97.56	97.98	93.92	97.98	98.70	97.48	95.11	95.65	99.68	97.45
	VDG19	97.82	97.78	99.01	96.22	98.60	99.41	94.81	97.55	97.05	98.77	96.70	99.19	99.64	97.13	99.38	94.92	99.23	99.40	97.25	96.88	99.78	99.36	98.28
	VDG15	97.25	94.77	98.88	95.84	96.77	98.29	95.75	96.97	78.69	99.54	99.48	97.83	99.73	90.98	96.84	92.49	99.33	94.51	96.77	96.47	98.80	99.71	97.45
	VDG68	91.98	97.41	99.74	95.16	98.11	98.42	94.68	96.83	90.16	98.85	96.53	99.57	96.77	97.56	97.45	99.45	95.97	96.41	90.24	96.06	98.37	99.39	97.75
	VDG3	97.71	97.14	98.65	87.15	99.48	99.25	94.81	94.81	84.26	98.31	98.09	96.71	98.48	95.97	98.86	94.81	97.21	96.31	94.18	85.87	97.50	99.39	98.46
	VDG50	98.28	97.86	99.24	96.75	98.72	99.27	94.81	95.53	89.18	99.16	97.05	97.95	99.82	97.24	99.65	94.70	95.68	94.91	97.88	88.99	99.13	95.44	98.40
	VDG67	97.14	99.47	99.64	94.03	97.01	99.41	95.21	97.98	84.26	99.16	95.14	99.75	98.92	98.09	97.36	94.14	96.73	97.50	98.43	90.76	99.24	97.88	98.82
	VDG60	96.68	98.42	99.24	92.29	95.33	97.24	93.24	97.40	79.02	99.69	95.66	94.85	99.64	99.26	95.96	98.95	97.79	94.31	90.87	98.37	99.13	98.64	97.39
	VDG12	99.20	96.39	99.08	93.50	96.43	99.77	96.60	96.97	90.82	99.46	94.62	99.01	99.10	93.31	99.82	93.48	97.69	92.61	92.92	92.26	99.02	97.47	96.68
	VDG4	98.05	97.71	99.64	96.52	96.73	99.75	94.50	97.69	83.93	97.85	98.44	97.08	98.39	97.45	97.80	94.03	99.90	99.40	97.09	91.30	98.48	97.27	99.59
	VDG41	99.66	98.12	99.80	97.88	99.82	99.34	94.94	99.28	91.80	98.39	94.10	99.32	97.85	94.59	98.42	95.97	97.41	97.60	97.17	98.91	98.80	99.27	98.58
	VDG23	98.74	98.65	99.50	97.13	99.15	99.68	95.75	99.28	95.74	98.47	99.48	98.63	97.76	96.71	99.65	94.09	99.23	97.90	97.95	95.79	99.67	99.16	98.52
	VDG28	98.63	99.02	99.47	96.67	97.53	99.66	95.17	97.84	92.46	97.62	98.96	98.70	97.31	99.47	99.03	90.55	99.52	98.00	96.77	98.64	98.70	98.95	99.17
	VDG18	97.14	98.42	99.67	95.84	99.27	99.98	95.84	94.66	94.75	99.54	97.57	99.07	98.12	99.89	99.65	93.81	95.49	98.20	95.83	95.38	97.83	99.56	98.16
	VDG40	97.82	94.55	98.78	97.43	97.80	99.38	94.90	99.42	95.74	98.77	98.09	98.32	98.30	97.98	96.57	93.98	97.79	96.91	97.80	98.78	93.70	97.44	96.63
	VDG29	99.77	99.25	99.37	99.47	99.05	99.93	95.35	99.42	95.41	99.00	98.09	98.39	99.37	95.33	98.86	93.81	96.16	94.31	96.62	88.59	99.13	99.01	97.16
	VDG35	97.94	94.02	97.79	98.79	96.43	97.74	94.85	89.18	89.84	99.23	96.88	99.19	97.85	90.66	97.28	93.54	97.69	97.50	93.00	93.48	99.02	96.98	99.11
	VDG5	98.51	98.65	98.88	97.05	98.60	98.77	95.30	99.71	86.23	99.16	97.92	98.88	99.55	96.71	98.51	92.60	95.49	92.42	93.31	98.51	98.80	98.37	99.23
	VDG13	96.22	98.35	99.47	97.58	96.77	99.00	94.99	91.92	96.72	98.39	98.09	98.01	98.66	93.95	98.51	91.77	98.75	92.81	98.58	88.32	98.70	98.29	96.21
	VDG36	98.97	96.95	99.77	97.73	97.31	99.54	95.17	97.69	95.74	98.62	98.96	98.20	98.66	98.09	98.42	93.70	94.33	97.11	97.01	95.65	99.57	99.39	98.99
	VDG46	98.28	92.59	98.22	96.67	99.12	98.56	96.38	96.10	95.74	99.08	97.40	99.32	99.10	85.88	99.65	87.51	97.79	94.51	93.94	97.28	98.70	96.25	97.99
	VDG33	99.08	97.67	99.80	95.84	98.69	99.79	96.55	98.41	76.72	97.47	88.72	98.51	99.37	93.95	98.95	92.82	98.85	99.60	93.31	97.15	98.48	98.08	97.69
	VDG17	98.63	98.16	98.78	99.77	98.96	99.84	96.11	96.97	96.07	99.62	99.31	99.01	99.91	97.98	99.65	94.59	98.85	99.00	97.56	98.78	97.28	99.36	99.41
	VDG47	98.17	98.01	99.64	94.26	97.56	99.63	94.77	88.74	89.18	98.77	87.85	99.32	99.64	87.26	95.69	90.50	95.87	93.31	95.52	91.71	99.02	97.65	96.57
	VDG10	98.40	94.32	98.61	93.65	95.79	99.13	95.70	96.39	88.52	98.85	96.88	99.13	95.52	97.24	98.51	94.48	92.41	97.90	90.95	91.98	99.02	95.56	97.45
	VDG26	98.74	98.68	99.34	98.49	99.48	98.81	95.08	96.39	98.03	99.00	99.13	99.63	99.10	92.25	99.21	94.53	99.62	96.11	97.72	93.61	97.93	99.54	99.53
Coverage depth (from white to red color represents the gene coverage depth of mapping reads from 0x to 22x)																								
	VDG66	6.4	0.0	0.0	0.0	0.0	1.0	0.0	0.0	0.0	0.0	0.0	0.0	0.0	0.0	0.0	1.0	0.0	0.0	0.0	0.0	0.0	0.0	0.0
	VDG24	6.5	1.1	1.0	1.0	1.0	1.0	1.8	1.0	0.0	1.0	1.0	1.0	0.0	1.0	1.6	1.0	1.0	0.0	1.0	0.0	1.3	0.0	0.0
	VDG30	9.2	0.0	0.0	0.0	0.0	1.0	0.0	0.0	0.0	0.0	0.0	0.0	1.0	0.0	0.0	1.0	0.0	0.0	0.0	0.0	0.0	0.0	0.0
	VDG2	7.4	0.0	0.0	0.0	0.0	1.0	0.0	0.0	0.0	1.0	1.0	0.0	1.0	0.0	0.0	0.0	0.0	0.0	0.0	0.0	0.0	0.0	0.0
	VDG56	6.5	0.0	0.0	0.0	0.0	0.0	0.0	0.0	0.0	0.0	0.0	0.0	0.0	0.0	0.0	0.0	0.0	0.0	0.0	0.0	0.0	0.0	0.0
	VDG54	6.5	0.0	0.0	1.0	0.0	0.0	0.0	0.0	0.0	1.0	0.0	0.0	0.0	0.0	0.0	0.0	0.0	0.0	0.0	0.0	0.0	0.0	0.0
	VDG58	6.7	0.0	0.0	0.0	0.0	0.0	0.0	0.0	0.0	0.0	0.0	0.0	0.0	0.0	0.0	0.0	0.0	0.0	0.0	0.0	0.0	0.0	0.0
ND	VDG59	6.2	0.0	0.0	0.0	0.0	1.0	0.0	0.0	0.0	0.0	0.0	0.0	0.0	0.0	0.0	0.0	0.0	0.0	0.0	0.0	0.0	0.0	0.0
	VDG78	6.9	0.0	1.0	0.0	0.0	0.0	0.0	0.0	0.0	0.0	0.0	0.0	0.0	0.0	0.0	0.0	0.0	0.0	0.0	0.0	0.0	0.0	0.0
	VDG62	6.2	1.0	0.0	0.0	0.0	0.0	0.0	0.0	0.0	0.0	0.0	0.0	0.0	0.0	0.0	0.0	0.0	0.0	0.0	0.0	0.0	0.0	0.0
	VDG71	6.5	0.0	0.0	0.0	0.0	0.0	0.0	0.0	0.0	0.0	0.0	0.0	0.0	0.0	0.0	0.0	0.0	0.0	0.0	0.0	0.0	0.0	0.0
	VDG77	7.4	0.0	0.0	1.0	0.0	0.0	0.0	0.0	0.0	0.0	0.0	0.0	0.0	0.0	0.0	0.0	0.0	0.0	0.0	0.0	0.0	0.0	0.0
	VDG65	3.7	0.0	0.0	0.0	0.0	0.0	0.0	0.0	0.0	0.0	0.0	0.0	0.0	0.0	0.0	0.0	0.0	0.0	0.0	0.0	0.0	0.0	0.0
	VDG69	8.7	0.0	0.0	0.0	0.0	0.0	0.0	0.0	0.0	0.0	0.0	1.0	0.0	0.0	0.0	0.0	0.0	0.0	0.0	0.0	0.0	0.0	0.0
	VDG70	7.8	0.0	0.0	0.0	0.0	0.0	0.0	0.0	0.0	0.0	0.0	0.0	0.0	0.0	1.0	0.0	0.0	0.0	0.0	0.0	0.0	0.0	0.0
	VDG74	6.4	0.0	0.0	0.0	1.0	0.0	1.0	0.0	0.0	0.0	1.0	0.0	0.0	0.0	0.0	0.0	0.0	0.0	0.0	0.0	0.0	1.0	0.0
	VDG39	16.0	11.0	18.0	7.3	16.0	15.0	15.0	15.0	16.0	22.0	20.0	15.0	19.0	16.0	16.0	13.0	18.0	11.0	13.0	12.0	17.0	15.0	12.0
	VDG73	8.9	6.5	9.7	4.7	7.1	8.1	8.4	8.2	8.8	13.0	13.0	8.7	9.9	7.0	9.3	5.7	9.6	8.1	7.8	5.6	11.0	7.1	7.4
	VDG32	12.0	9.3	15.0	7.2	11.0	13.0	13.0	9.7	12.0	14.0	15.0	14.0	14.0	11.0	13.0	9.5	15.0	11.0	10.0	9.8	16.0	12.0	9.4
	VDG63	9.2	8.0	10.0	5.5	8.8	8.7	11.0	7.1	6.9	15.0	13.0	11.0	11.0	8.3	9.0	8.4	8.5	9.5	7.0	9.3	12.0	7.2	8.0
	VDG75	9.0	6.6	11.0	4.5	8.6	8.3	8.5	6.6	8.8	11.0	9.3	9.5	13.0	7.7	8.5	9.5	9.6	7.9	6.0	7.8	13.0	8.5	8.3
	VDG57	9.7	6.2	9.2	5.1	6.2	7.8	9.3	7.8	9.3	12.0	11.0	8.5	12.0	8.6	7.5	5.9	8.9	8.3	6.1	6.9	12.0	7.3	6.3
	VDG14	9.5	6.6	9.8	4.7	7.7	8.2	8.2	5.9	8.2	13.0	11.0	8.6	10.0	7.8	9.3	7.3	8.3	5.9	5.8	5.8	12.0	7.5	6.3
	VDG49	11.0	7.0	11.0	5.3	8.9	8.0	9.9	7.0	8.5	13.0	11.0	11.0	12.0	7.3	8.0	9.4	7.8	6.5	8.3	8.5	14.0	8.2	7.3
	VDG22	11.0	9.0	12.0	4.5	8.5	9.5	10.0	8.3	7.9	13.0	13.0	12.0	10.0	9.1	10.0	8.1	12.0	8.8	7.1	10.0	13.0	9.1	7.0
	VDG61	8.0	7.3	11.0	4.2	5.9	8.5	8.1	8.4	8.2	14.0	8.2	8.1	11.0	7.6	9.6	7.1	9.9	4.8	6.6	7.8	11.0	7.8	7.3
	VDG27	10.0	8.6	15.0	7.3	12.0	11.0	11.0	12.0	9.0	17.0	17.0	13.0	14.0	10.0	12.0	9.0	14.0	9.2	8.4	11.0	16.0	10.0	8.8
	VDG9	9.7	7.1	8.7	3.8	6.9	6.6	6.2	8.0	7.6	11.0	9.7	7.5	8.7	7.7	7.3	5.9	7.8	6.5	5.4	7.1	11.0	6.1	5.7
	VDG21	15.0	9.8	15.0	8.5	12.0	11.0	13.0	11.0	12.0	18.0	16.0	14.0	12.0	11.0	12.0	10.0	13.0	11.0	8.9	11.0	17.0	12.0	8.8
	VDG52	11.0	6.2	9.9	3.8	6.9	7.3	7.3	7.8	7.5	10.0	7.7	8.5	12.0	8.4	6.9	8.1	11.0	6.7	6.8	7.2	12.0	8.5	7.3
	VDG16	9.4	7.3	10.0	4.4	7.2	6.6	7.8	7.6	7.1	11.0	9.1	7.5	9.9	5.2	7.2	5.2	7.4	6.8	5.6	8.0	9.7	6.1	5.7
	VDG76	9.5	6.1	9.9	5.2	6.6	7.8	9.1	7.1	8.6	13.0	11.0	9.2	9.3	7.3	6.0	5.9	9.6	5.8	5.0	6.7	11.0	6.4	7.3
	VDG8	11.0	5.9	9.5	4.6	6.3	8.1	8.3	6.2	6.6	12.0	9.9	8.6	8.8	6.0	8.4	6.4	7.9	4.7	6.9	9.6	11.0	8.3	7.3
	VDG48	5.6	6.2	7.0	4.9	6.2	7.3	8.2	6.4	7.0	9.0	7.6	7.0	8.1	7.1	7.6	5.9	8.5	5.6	6.5	5.9	9.4	7.0	5.9
	VDG42	11.0	9.9	15.0	10.0	11.0	12.0	12.0	7.9	12.0	16.0	14.0	15.0	17.0	11.0	12.0	10.0	14.0	11.0	10.0	9.2	14.0	10.0	11.0
	VDG37	9.2	7.2	10.0	4.7	7.3	8.6	9.0	6.6	12.0	13.0	11.0	12.0	9.3	8.6	8.2	7.2	8.5	7.7	7.4	7.9	11.0	6.9	6.6

Isolates		VEDA_05181	VEDA_05182	VEDA_05183	VEDA_05184	VEDA_05185	VEDA_05186	VEDA_05187	VEDA_05188	VEDA_05189	VEDA_05190	VEDA_05191	VEDA_05192	VEDA_05193	VEDA_05194	VEDA_05195	VEDA_05196	VEDA_05197	VEDA_05198	VEDA_05199	VEDA_05200	VEDA_05201	VEDA_05202	VEDA_05203
	VDG43	8.6	5.9	8.9	4.4	7.1	8.4	7.4	7.5	5.7	12.0	11.0	7.3	11.0	7.4	8.1	7.2	6.5	7.4	7.4	6.9	8.5	7.2	8.0
	VDG6	9.2	6.3	9.6	4.7	7.9	7.6	8.3	6.2	4.9	14.0	14.0	9.1	10.0	8.8	7.7	7.4	8.0	7.9	7.0	8.6	12.0	7.4	7.3
	VDG11	8.6	6.1	8.8	4.6	6.0	6.9	7.8	6.9	7.9	13.0	8.7	9.3	8.7	8.0	10.0	6.6	9.7	4.1	5.0	8.3	7.5	7.0	5.3
	VDG44	11.0	10.0	15.0	6.4	12.0	14.0	15.0	11.0	9.9	19.0	17.0	14.0	13.0	12.0	12.0	14.0	14.0	11.0	10.0	11.0	18.0	12.0	13.0
	VDG51	8.1	6.8	11.0	4.1	7.0	7.7	7.9	6.8	8.6	13.0	13.0	8.7	9.6	8.3	8.0	6.2	8.8	6.8	6.2	6.3	11.0	7.3	7.6
	VDG64	12.0	8.1	11.0	4.7	8.3	8.7	10.0	7.9	7.0	14.0	14.0	12.0	10.0	9.1	9.7	7.8	11.0	8.3	7.4	8.4	14.0	9.1	8.5
	VDG45	10.0	8.6	12.0	6.2	9.9	10.0	11.0	9.6	11.0	14.0	14.0	12.0	13.0	10.0	8.5	8.2	11.0	7.2	8.3	9.0	13.0	10.0	8.7
	VDG53	9.1	7.4	11.0	4.1	7.3	8.2	8.7	7.3	8.7	14.0	12.0	9.5	13.0	9.6	8.0	8.2	7.9	8.5	7.3	6.2	12.0	8.2	7.7
	VDG25	16.0	11.0	15.0	6.8	11.0	13.0	12.0	12.0	8.8	17.0	16.0	14.0	15.0	12.0	12.0	12.0	14.0	10.0	12.0	11.0	19.0	11.0	9.4
D	VDG7	7.2	6.4	8.1	3.9	7.2	8.1	7.6	5.2	4.5	9.4	9.7	6.6	8.8	8.1	7.7	6.7	6.5	7.2	7.1	6.0	8.7	6.6	6.2
	VDG31	16.0	10.0	17.0	10.0	14.0	13.0	14.0	12.0	11.0	19.0	22.0	14.0	16.0	12.0	12.0	10.0	14.0	13.0	9.9	9.0	13.0	11.0	13.0
	VDG38	17.0	10.0	13.0	7.7	11.0	13.0	13.0	11.0	10.0	21.0	16.0	13.0	15.0	13.0	11.0	11.0	13.0	12.0	9.4	11.0	15.0	11.0	9.2
	VDG20	10.0	6.8	10.0	6.8	8.5	9.3	10.0	9.6	6.9	15.0	13.0	11.0	13.0	9.2	8.6	8.0	9.7	10.0	7.4	8.8	13.0	8.8	8.3
	VDG34	14.0	9.4	14.0	8.7	10.0	11.0	11.0	9.4	10.0	17.0	15.0	13.0	15.0	9.8	10.0	10.0	13.0	10.0	7.7	9.1	13.0	11.0	8.6
	VDG19	14.0	9.2	15.0	6.2	12.0	12.0	13.0	13.0	12.0	17.0	14.0	16.0	12.0	11.0	13.0	12.0	12.0	11.0	8.7	13.0	15.0	13.0	9.9
	VDG15	9.2	6.5	8.4	5.2	7.9	7.7	7.3	9.4	6.0	12.0	13.0	9.2	8.7	7.7	9.9	8.4	8.4	7.1	7.0	7.6	13.0	9.2	8.5
	VDG68	8.2	7.9	8.9	5.9	7.6	7.9	9.2	7.0	9.5	11.0	12.0	8.7	10.0	7.9	8.0	7.6	10.0	5.4	8.3	7.9	11.0	7.2	8.0
	VDG3	8.4	6.8	10.0	5.5	7.2	7.0	9.1	6.4	8.1	12.0	13.0	7.7	10.0	7.2	6.6	6.8	9.5	8.5	5.7	6.7	11.0	7.2	6.9
	VDG50	14.0	6.9	9.7	5.1	8.9	8.9	8.6	9.2	9.6	17.0	13.0	9.4	11.0	9.4	13.0	7.8	11.0	5.4	6.4	9.8	16.0	10.0	6.7
	VDG67	8.7	9.1	11.0	5.4	8.3	10.0	9.9	7.6	7.0	15.0	14.0	8.7	8.5	9.8	7.9	9.5	12.0	7.9	6.4	8.1	10.0	9.4	6.7
	VDG60	7.0	5.8	8.4	4.0	6.0	7.1	6.3	6.7	6.0	10.0	6.5	7.2	7.4	6.3	6.5	5.3	6.7	5.3	4.0	7.5	9.7	6.5	6.1
	VDG12	11.0	6.5	10.0	4.4	6.9	7.4	9.4	8.3	7.8	12.0	8.9	11.0	11.0	8.1	7.0	6.7	6.5	6.6	5.4	8.7	11.0	7.9	6.6
	VDG4	9.6	6.0	8.8	4.1	7.6	7.3	6.1	7.2	4.6	10.0	7.8	7.6	7.5	8.1	6.6	7.1	7.9	6.6	5.3	6.0	9.7	6.5	5.8
	VDG41	9.4	8.0	11.0	6.4	8.5	9.2	11.0	7.7	5.8	12.0	11.0	11.0	11.0	11.0	9.5	8.9	10.0	9.0	8.0	7.9	13.0	8.7	8.0
	VDG23	13.0	11.0	15.0	8.5	13.0	13.0	14.0	12.0	12.0	19.0	18.0	13.0	14.0	14.0	14.0	9.1	15.0	10.0	10.0	11.0	17.0	12.0	9.1
	VDG28	7.8	6.1	9.2	4.4	6.5	8.0	9.1	5.4	7.0	12.0	11.0	9.1	8.2	7.8	7.7	7.0	9.2	6.7	6.0	6.3	10.0	7.4	6.6
	VDG18	11.0	8.2	12.0	5.0	9.4	10.0	8.9	7.4	8.4	14.0	9.6	11.0	9.6	10.0	9.8	8.2	9.8	8.4	7.3	8.5	12.0	8.6	6.9
	VDG40	7.2	5.5	10.0	5.7	7.0	6.8	8.8	6.1	6.5	11.0	11.0	8.9	9.3	7.1	6.7	7.1	7.1	5.8	5.4	5.4	11.0	6.8	6.1
	VDG29	11.0	11.0	14.0	7.3	9.8	10.0	11.0	8.4	11.0	17.0	12.0	14.0	12.0	10.0	14.0	11.0	12.0	11.0	8.7	9.4	14.0	11.0	10.0
	VDG35	12.0	6.1	9.9	4.9	6.2	6.5	7.0	7.4	6.9	15.0	11.0	7.5	9.4	7.5	8.1	6.4	9.1	7.7	5.4	7.3	12.0	8.0	6.0
	VDG5	12.0	6.6	8.7	4.9	6.3	7.4	7.5	6.5	6.2	9.9	11.0	7.6	7.6	8.4	6.9	6.5	7.3	7.0	6.3	6.1	10.0	6.2	7.2
	VDG13	10.0	6.8	8.8	5.6	7.2	7.6	8.6	6.9	7.3	11.0	9.4	8.4	10.0	8.3	8.2	7.3	9.9	8.5	6.5	6.5	13.0	7.4	7.0
	VDG36	13.0	10.0	15.0	7.5	12.0	12.0	12.0	8.9	6.2	15.0	14.0	13.0	14.0	8.9	11.0	10.0	15.0	10.0	7.7	13.0	16.0	9.8	9.6
	VDG46	7.9	5.7	8.1	4.4	6.8	6.4	6.4	6.1	8.9	11.0	10.0	8.3	9.1	9.0	8.3	6.0	8.1	6.3	4.6	6.2	11.0	6.2	4.7
	VDG33	11.0	8.2	11.0	4.2	7.8	8.5	9.5	7.0	12.0	15.0	9.5	9.3	11.0	8.4	7.2	7.4	10.0	6.3	7.3	8.3	13.0	8.9	8.1
	VDG17	12.0	9.5	15.0	6.0	11.0	11.0	12.0	8.7	13.0	14.0	17.0	14.0	13.0	11.0	11.0	11.0	12.0	11.0	8.8	12.0	16.0	11.0	9.1
	VDG47	8.7	7.2	9.2	4.4	8.3	7.5	8.2	6.0	7.9	10.0	11.0	9.1	7.9	8.5	7.0	7.0	7.0	5.7	7.0	7.2	12.0	6.6	6.9
	VDG10	8.1	5.7	8.9	4.4	6.8	7.3	8.4	6.7	8.7	11.0	11.0	8.7	9.3	7.0	7.9	6.2	8.1	8.7	5.0	7.8	12.0	8.1	7.6
	VDG26	15.0	8.5	12.0	6.4	9.2	9.7	9.9	8.4	8.7	14.0	15.0	9.8	13.0	8.8	8.9	8.6	13.0	7.5	8.7	9.8	12.0	8.3	8.0

Header cell: Gene ID / Isolates

Table S4 Information of lineage-specific genes in Vd991

Gene-ID-V991	Gene Length	Scaffold:position	GeneBank-ID	NR_define	Homolog functional of species	Interpro
VEDA_00818	1274	Scaffold12:670547-671821	XP_003006162.1	conserved hypothetical protein	Verticillium alfalfae VaMs.102	
VEDA_00819	3951	Scaffold12:672371-676322	EQB56593.1	hypothetical protein	Colletotrichum gloeosporioides Cg-14	IPR021840; Protein of unknown function DUF3433
VEDA_02653	1280	Scaffold33:262046-263326	EQB45048.1	hypothetical protein	Colletotrichum gloeosporioides Cg-14	IPR011050; Pectin lyase fold/virulence factor//IPR012334; Pectin lyase fold
VEDA_02654	1121	Scaffold33:264741-265862	EWZ01097.1	hypothetical protein	Fusarium oxysporum FOSC 3-a	IPR013830; Esterase, SGNH hydrolase-type//IPR013831; Esterase, SGNH hydrolase-type, subgroup
VEDA_03154	826	Scaffold22:56576-57402	NA			
VEDA_03264	578	Scaffold22:471344-471922	XP_003007566.1	ankyrin-1	Verticillium alfalfae VaMs.102	IPR002110; Ankyrin repeat//IPR020683; Ankyrin repeat-containing domain
VEDA_03298	566	Scaffold22:576180-576746	XP_009654226.1	hypothetical protein	Verticillium dahliae VdLs.17	
VEDA_03785	492	Scaffold30:146548-147040	XP_009654226.1	hypothetical protein	Verticillium dahliae VdLs.17	
VEDA_03860	992	Scaffold11:127507-128499	XP_009653772.1	hypothetical protein	Verticillium dahliae VdLs.17	
VEDA_03864	2409	Scaffold11:143861-146270	CCF45959.1	hypothetical protein	Colletotrichum higginsianum	
VEDA_05182	2659	Scaffold4:261421-264079	EXK78782.1	hypothetical protein	Fusarium oxysporum f. sp. raphani 54005	IPR021842: Protein of unknown function DUF3435
VEDA_05183	3029	Scaffold4:265251-268279	XP_002558475.1	Pc13g00220	Penicillium rubens Wisconsin 54-1255	IPR001440: Tetratricopeptide TPR-1; IPR002151: Kinesin light chain; IPR011990: Tetratricopeptide-like helical; IPR013026: Tetratricopeptide repeat-containing domain; IPR019734: Tetratricopeptide repeat
VEDA_05184	1323	Scaffold4:268562-269884	EHK48651.1	hypothetical protein	Trichoderma atroviride IMI 206040	IPR000845: Nucleoside phosphorylase domain
VEDA_05185	3277	Scaffold4:270400-273676	CEJ92447.1	hypothetical protein	Torrubiella hemipterigena	IPR001440: Tetratricopeptide TPR-1; IPR002151: Kinesin light chain; IPR011990: Tetratricopeptide-like helical; IPR013026: Tetratricopeptide repeat-containing domain; IPR019734: Tetratricopeptide repeat
VEDA_05186	4380	Scaffold4:280589-284968	KFA80388.1	hypothetical protein	Stachybotrys chartarum IBT 40288	IPR001680: WD40 repeat; IPR007111: NACHT nucleoside triphosphatase; IPR011047: Quinonprotein alcohol dehydrogenase-like; IPR015943: WD40/YVTN repeat-like-containing domain; IPR017986: WD40-repeat-containing domain; IPR020472: G-protein beta WD-40 repeat
VEDA_05187	2235	Scaffold4:289823-292057	EPE03243.1	serine threonine protein kinase	Ophiostoma piceae UAMH 11346	IPR000719: Protein kinase, catalytic domain; IPR011009: Protein kinase-like domain
VEDA_05188	693	Scaffold4:294043-294735	XP_006965840.1	predicted protein	Trichoderma reesei QM6a	
VEDA_05189	305	Scaffold4:299108-299412	EWZ78678.1	hypothetical protein	Fusarium oxysporum f. sp. lycopersici MN25	
VEDA_05190	1305	Scaffold4:301918-303222	EXL39785.1	hypothetical protein	Fusarium oxysporum f. sp. radicis-lycopersici 26381	IPR003593: AAA+ ATPase domain; IPR003959: ATPase, AAA-type, core; IPR003960: ATPase, AAA-type, conserved site; IPR014851: BCS1, N-terminal
VEDA_05191	576	Scaffold4:307357-307932	EXK77641.1	hypothetical protein	Fusarium oxysporum f. sp. raphani 54005	
VEDA_05192	1611	Scaffold4:313225-314835	EXL40444.1	hypothetical protein	Fusarium oxysporum f. sp. radicis-lycopersici 26381	IPR007111: NACHT nucleoside triphosphatase
VEDA_05193	1116	Scaffold4:317053-318168	EXM13674.1	hypothetical protein	Fusarium oxysporum f. sp. vasinfectum 25433	IPR011051: Cupin, RmlC-type; IPR013096: Cupin 2, conserved barrel; IPR014710: RmlC-like jelly roll fold
VEDA_05194	942	Scaffold4:323081-324022	KFY64054.1	hypothetical protein	Pseudogymnoascus pannorum VKM F-4515 (FW-2607)	IPR008030: NmrA-like; IPR016040: NAD(P)-binding domain
VEDA_05195	1138	Scaffold4:327568-328705	EXM13678.1	hypothetical protein	Fusarium oxysporum f. sp. vasinfectum 25433	IPR000172: Glucose-methanol-choline oxidoreductase, N-terminal
VEDA_05196	1810	Scaffold4:332126-333935	EXM14904.1	hypothetical protein	Fusarium oxysporum f. sp. vasinfectum 25433	IPR011701: Major facilitator superfamily; IPR016196: Major facilitator superfamily domain, general substrate transporter; IPR020846: Major facilitator superfamily domain
VEDA_05197	1041	Scaffold4:335027-336067	EXM14903.1	hypothetical protein	Fusarium oxysporum f. sp. vasinfectum 25433	IPR002198: Short-chain dehydrogenase/reductase SDR; IPR002347: Glucose/ribitol dehydrogenase; IPR016040: NAD(P)-binding domain; IPR013968: Polyketide synthase, ketoreductase domain
VEDA_05198	1002	Scaffold4:336752-337753	EXM14902.1	hypothetical protein	Fusarium oxysporum f. sp. vasinfectum 25433	IPR002085: Alcohol dehydrogenase superfamily, zinc-type; IPR011032: GroES-like; IPR013154: Alcohol dehydrogenase GroES-like; IPR016040: NAD(P)-binding domain; IPR020843: Polyketide synthase, enoylreductase
VEDA_05199	1271	Scaffold4:338456-339726	EXM14901.1	hypothetical protein	Fusarium oxysporum f. sp. vasinfectum 25433	IPR036866: Metallo-hydrolase/oxidoreductase superfamily; IPR024884: N-acyl-phosphatidylethanolamine-hydrolysing phospholipase D; IPR001279: Metallo-beta-lactamase
VEDA_05200	736	Scaffold4:342673-343408	XP_003050975.1	hypothetical protein	Nectria haematococca mpVI 77-13-4	IPR001137: Glycoside hydrolase, family 11; IPR008985: Concanavalin A-like lectin/glucanase; IPR013319: Glycoside hydrolase, family 11/12, catalytic domain; IPR018208: Glycoside hydrolase, family 11, active site
VEDA_05201	920	Scaffold4:344956-345875	KID86625.1	NAD(P)-binding domain protein	Metarhizium guizhouense ARSEF 977	IPR002198: Short-chain dehydrogenase/reductase SDR; IPR002347: Glucose/ribitol dehydrogenase; IPR016040: NAD(P)-binding domain
VEDA_05202	3444	Scaffold4:349504-352947	KID83784.1	ATP-dependent DNA helicase PIF1	Metarhizium guizhouense ARSEF 977	IPR010285: DNA helicase PIF1, ATP-dependent; IPR025476: Helitron helicase-like domain; IPR027417: P-loop containing nucleoside triphosphate hydrolase
VEDA_05203	1689	Scaffold4:353626-355314	EXK83419.1	hypothetical protein	Fusarium oxysporum f. sp. raphani 54005	IPR009057: Homeodomain-like; IPR017877: Myb-like domain; IPR001005: SANT/Myb domain
VEDA_05431	194	Scaffold4:1069467-1069661	XP_009650613.1	hypothetical protein	Verticillium dahliae VdLs.17	
VEDA_05599	732	Scaffold5:178324-179056				
VEDA_05600	780	Scaffold5:179492-180272	XP_009653600.1	hypothetical protein	Verticillium dahliae VdLs.17	
VEDA_06247	690	Scaffold10:835156-835846	XP_009651369.1	hypothetical protein	Verticillium dahliae VdLs.17	
VEDA_06248	1689	Scaffold10:839301-840990	XP_007751432.1	hypothetical protein	Cladophialophora psammophila CBS 110553	IPR013112; FAD-binding 8//IPR013121; Ferric reductase, NAD binding//IPR017927; Ferredoxin reductase-type FAD-binding domain
VEDA_06249	1357	Scaffold10:842338-843695	EFQ28084.1	protein kinase domain-containing prote	Colletotrichum graminicola M1.001	IPR000719; Protein kinase, catalytic domain//IPR002290; Serine/threonine- / dual-specificity protein kinase, catalytic domain//IPR011009; Protein kinase-like domain//IPR017441; Protein kinase, ATP binding site

Gene-ID-V991	Gene Length	Scaffold:position	GeneBank-ID	NR_define	Homolog functional of species	Interpro
VEDA_06250	1167	Scaffold10:848311-849478	XP_003001163.1	predicted protein	Verticillium alfalfae VaMs.102	IPR002112; Transcription factor Jun//IPR004827; Basic-leucine zipper domain//IPR011616; bZIP transcription factor, bZIP-1
VEDA_06251	482	Scaffold10:849968-850450	XP_009651370.1	hypothetical protein	Verticillium dahliae VdLs.17	
VEDA_06252	1150	Scaffold10:850478-851628	XP_009651369.1	hypothetical protein	Verticillium dahliae VdLs.17	
VEDA_06253	837	Scaffold10:856322-857159	EXK78667.1	hypothetical protein	Fusarium oxysporum f. sp. raphani 54005	IPR016035; Acyl transferase/acyl hydrolase/lysophospholipase
VEDA_06254	1082	Scaffold10:858294-859376	XP_007275581.1	lysine -specific demethylase 4c-like pro	Colletotrichum gloeosporioides Nara gc5	
VEDA_06334	1028	Scaffold13:66109-67137	XP_003003042.1	serine/threonine-protein kinase SRPK3	Verticillium alfalfae VaMs.102	IPR000719; Protein kinase, catalytic domain//IPR002290; Serine/threonine- / dual-specificity protein kinase, catalytic domain//IPR011009; Protein kinase-like domain
VEDA_06688	434	Scaffold17:185586-186020	NA			
VEDA_06794	558	Scaffold17:552240-552798	NA			
VEDA_06795	1529	Scaffold17:553579-555108	XP_003003132.1	carboxylesterase	Verticillium alfalfae VaMs.102	IPR002018; Carboxylesterase, type B//IPR019826; Carboxylesterase type B, active site
VEDA_06796	4211	Scaffold17:555393-559604	XP_003052741.1	hypothetical protein	Nectria haematococca mpVI 77-13-4	IPR001680; WD40 repeat//IPR015943; WD40/YVTN repeat-like-containing domain//IPR017986; WD40-repeat-containing domain
VEDA_06797	536	Scaffold17:560747-561283	EWY85490.1	hypothetical protein	Fusarium oxysporum FOSC 3-a	IPR002125; CMP/dCMP deaminase, zinc-binding//IPR004794; Riboflavin biosynthesis protein RibD//IPR016193; Cytidine deaminase-like
VEDA_06798	1175	Scaffold17:561769-562944	EXM26980.1	hypothetical protein	Fusarium oxysporum f. sp. vasinfectum 25433	IPR011009; Protein kinase-like domain//IPR016477; Fructosamine/Ketosamine-3-kinase
VEDA_07273	902	Scaffold3:419701-420603	XP_009655047.1	hypothetical protein	Verticillium dahliae VdLs.17	IPR012337; Ribonuclease H-like domain
VEDA_08924	1431	Scaffold28:263615-265046	XP_003003876.1	prostacyclin synthase	Verticillium alfalfae VaMs.102	IPR001128; Cytochrome P450//IPR002403; Cytochrome P450, E-class, group IV//IPR017972; Cytochrome P450, conserved site
VEDA_09258	3145	Scaffold9:757127-760272	XP_003003517.1	thiol-specific monooxygenase	Verticillium alfalfae VaMs.102	IPR001155; NADH:flavin oxidoreductase/NADH oxidase, N-terminal//IPR013785; Aldolase-type TIM barrel//IPR020946; Flavin monooxygenase-like
VEDA_09629	2636	Scaffold14:532800-535436	EXK78782.1	hypothetical protein	Fusarium oxysporum f. sp. raphani 54005	IPR021842; Protein of unknown function DUF3435
VEDA_09630	3350	Scaffold14:536319-539669	EWY97447.1	hypothetical protein	Fusarium oxysporum FOSC 3-a	IPR002151; Kinesin light chain//IPR011990; Tetratricopeptide-like helical
VEDA_09631	770	Scaffold14:543591-544361	GAA92066.1	transposase	Aspergillus kawachii IFO 4308	IPR012337; Ribonuclease H-like domain
VEDA_09632	1301	Scaffold14:544402-545703	EXK77146.1	hypothetical protein	Fusarium oxysporum f. sp. raphani 54005	
VEDA_09633	1010	Scaffold14:546543-547553	EXM14074.1	hypothetical protein	Fusarium oxysporum f. sp. vasinfectum 25433	IPR000845; Nucleoside phosphorylase domain
VEDA_09634	1877	Scaffold14:548118-549995	CDP31375.1	Putative Kinesin light chain	Podospora anserina S mat+	IPR011990; Tetratricopeptide-like helical//IPR013026; Tetratricopeptide repeat-containing domain//IPR019734; Tetratricopeptide repeat
VEDA_09635	452	Scaffold14:557825-558277	XP_003039520.1	predicted protein	Nectria haematococca mpVI 77-13-4	
VEDA_09636	2246	Scaffold14:560122-562368	EXL39778.1	hypothetical protein	Fusarium oxysporum f. sp. radicis-lycopersici 26381	IPR000719; Protein kinase, catalytic domain//IPR011009; Protein kinase-like domain
VEDA_09637	1496	Scaffold14:570300-571796	EXL39785.1	hypothetical protein	Fusarium oxysporum f. sp. radicis-lycopersici 26381	IPR003593; AAA+ ATPase domain//IPR003959; ATPase, AAA-type, core//IPR003960; ATPase, AAA-type, conserved site//IPR014851; BCS1, N-terminal
VEDA_09638	1823	Scaffold14:575037-576860	XP_007912226.1	putative het-s domain protein	Togninia minima UCRPA7	IPR000719; Protein kinase, catalytic domain//IPR011009; Protein kinase-like domain
VEDA_09639	5413	Scaffold14:577161-582574	KFY18250.1	hypothetical protein	Pseudogymnoascus pannorum VKM F-4246	IPR000719; Protein kinase, catalytic domain//IPR001680; WD40 repeat//IPR002290; Serine/threonine- / dual-specificity protein kinase, catalytic domain//IPR008271; Serine/threonine-protein kinase, active site//IPR011009; Protein kinase-like domain//IPR015943; WD40/YVTN repeat-like-containing domain//IPR017986; WD40-repeat-containing domain//IPR019775; WD40 repeat, conserved site//IPR020472; G-protein beta WD-40 repeat//IPR020635; Tyrosine-protein kinase, catalytic domain
VEDA_09640	1715	Scaffold14:583595-585310	EXL90226.1	hypothetical protein	Fusarium oxysporum f. sp. cubense tropical race 4 54006	
VEDA_09805	1775	Scaffold58:21955-23730	KFA69517.1	hypothetical protein	Stachybotrys chlorohalonata IBT 40285	IPR011701; Major facilitator superfamily//IPR016196; Major facilitator superfamily domain, general substrate transporter

Note: lines in yellow color represent G-LSR2, text in red color represent seven genes in this study.
